# Strategies for Boosting Performance of Perovskite Photodetectors: Synergistic Material and Device Optimization for Advanced Applications

**DOI:** 10.1007/s40820-026-02335-0

**Published:** 2026-10-01

**Authors:** Ying Li, Xinmiao Li, Yancheng Chen, Guozhen Shen

**Affiliations:** 1 School of Integrated Circuits and Electronics, Beijing Institute of Technology, Beijing, 100081 People’s Republic of China; 2 Key Laboratory of Optoelectronics Technology of Education, School of Information Science and Technology, Beijing University of Technology, Beijing, 100124 People’s Republic of China; 3 Tangshan Research Institute, Beijing Institute of Technology, Tangshan, 063000 People’s Republic of China

**Keywords:** Perovskite, Photodetector, Materials engineering, Performance optimization strategies, Optoelectronic application

## Abstract

**Highlights:**

- A systematic analysis of performance enhancement strategies for perovskite photodetectors, covering past decades of research from the initial exploration to the practical applications, has been provided.
- Performance optimization strategies centered on material preparation, optical management, interface engineering, and stability improvements have been elaborated from a synergistic perspective.
- Persistent challenges of perovskite photodetectors application demands have been addressed, highlighting a roadmap for the continued advancement and practical application of perovskite photodetectors.

**Abstract:**

Photodetectors (PDs), which are core components of modern optoelectronic systems, are widely used in medical diagnostics and biosensing. To meet the demands of next-generation optoelectronic technologies for high performance, low cost, and portability, perovskite materials have emerged as highly promising light-absorbing materials due to their excellent optoelectronic properties, solution processability, and compositional flexibility. Recently, significant performance improvements in perovskite-based PDs have been achieved through various strategies. This review systematically summarizes and analyzes performance enhancement strategies for perovskite PDs. First, the fundamental working mechanisms and key performance parameters of PDs are introduced. Then, four core strategies for performance improvement are discussed in detail. In material preparation, methods for regulating the growth of high-quality light absorption layers are reviewed. In light management, technical approaches such as plasmonic effects, optical microcavities, and nanostructures for enhancing light-harvesting efficiency are summarized. In interface engineering, the impacts of energy level alignment, interface passivation, and charge transport layer optimization on device performance are analyzed. Concerning stability enhancement, the impacts of crystal growth control, additive engineering, and encapsulation technology on device performance are summarized and analyzed. The review further examines the representative applications of perovskite PDs. Finally, key challenges such as stability and large-area fabrication are discussed, along with perspectives on future development. This review aims not only to summarize the performance optimization strategies for perovskite PDs but also to provide a roadmap for their future development and exploration of applications in industrial manufacturing, daily life, and healthcare.

**Figure:**
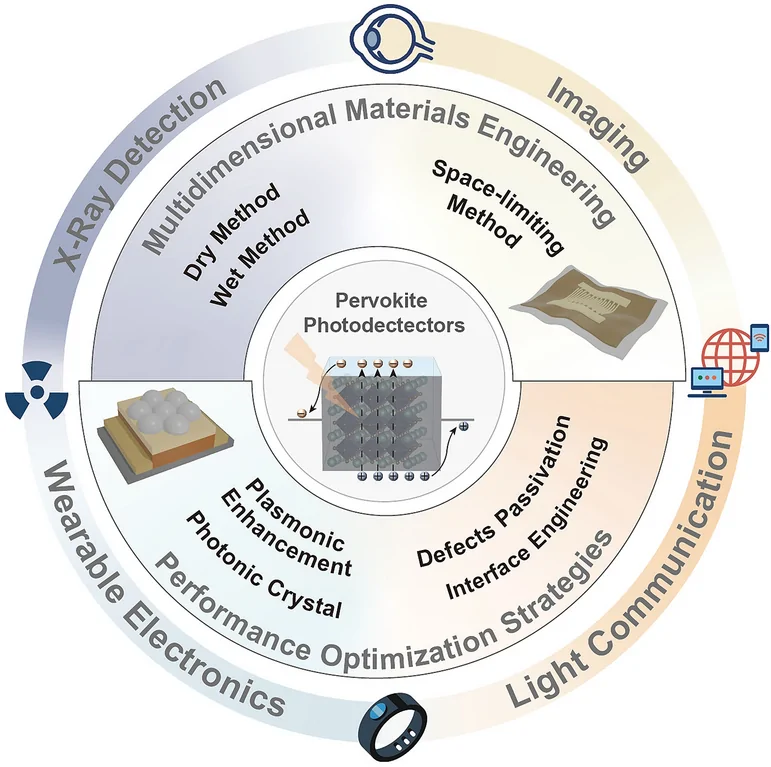

## Introduction

Photodetectors (PDs) represent a cornerstone technology in modern optoelectronics. As a key interface between optical and electronic systems, they have become indispensable components in a wide range of scientific, industrial, and consumer applications. Currently, various types of PDs based on traditional materials such as Si and InGaAs can detect the presence, intensity, wavelength, phase, and polarization of light, covering a broad spectrum from ultraviolet (UV) to infrared and even terahertz wavelengths [1]. However, the fabrication of these materials is constrained by relatively complex preparation processes and expensive growth equipment. Moreover, with the advancement of integrated circuit science and technology in the post-Moore era, innovations in device structures and integrated designs are driving PDs toward smaller sizes, higher performance, multifunctional integration, and intelligent development [2, 3]. Therefore, there is an urgent need to develop PDs capable of meeting the aforementioned requirements.

**Fig. 1:**
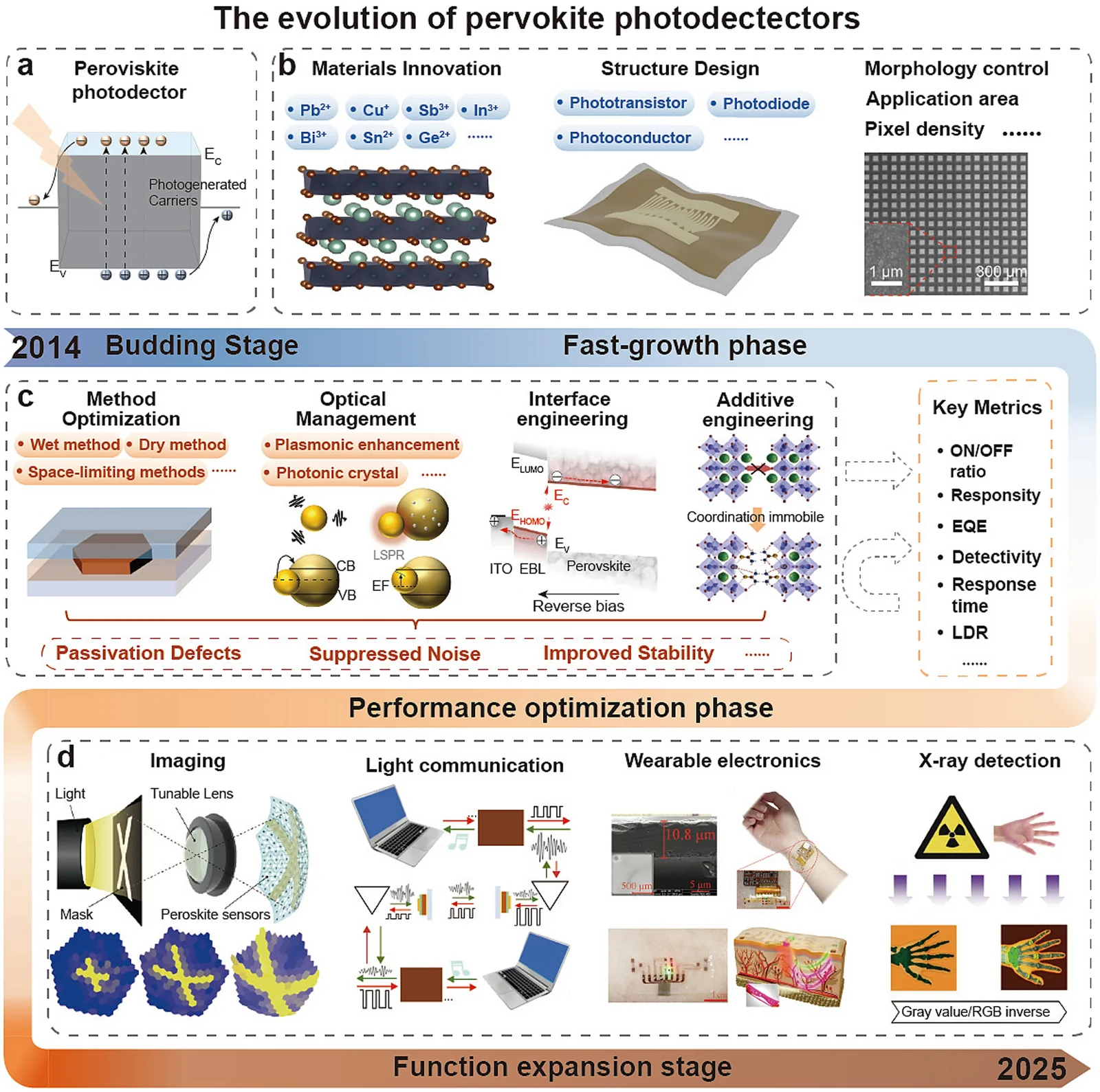
Development history of perovskite PDs. **a** Schematic of perovskite PDs. **b** Fast-growth phase of perovskite PDs, with emphasis on material innovation, structural design and crystalline morphology optimization. Reproduced with permission from Ref. [26] Copyright 2023, Wiley. **c** Device performance optimization phase of perovskite PDs by developing several strategies including method optimization, optical management, interface engineering, and additive engineering. Reproduced with permission from Ref. [27] Copyright 2021, Springer Nature. Reproduced with permission from Ref. [28] Copyright 2021, Springer Nature. **d** Function expansion stage encompassing applications such as imaging, optical communications, wearable electronics, and X-ray detection. Reproduced with permission from Ref. [29] Copyright 2025, AAAS. Reproduced with permission from Ref. [30] Copyright 2020, Springer Nature. Reproduced with permission from Ref. [31] Copyright 2020, Oxford University Press. Reproduced with permission from Ref. [32] Copyright 2023, AAAS

In the past few years, halide perovskites with the ABX_3_ structure (where A = CH_3_NH_3_ (MA), NH_2_CHNH_2_ (FA), or Cs; B = Pb; X = Cl, Br, I, or mixed halides) have attracted intensive research interest since their landmark introduction in dye-sensitized solar cells by Miyasaka and colleagues in 2009 [4–7]. Owing to their outstanding optoelectronic properties, tunable optical characteristics, and facile fabrication techniques, these materials have demonstrated significant potential as active layers for simple, low-cost, and large-area PDs [8, 9]. Since the initial investigation into the photodetection capabilities of metal halide perovskites in 2014, this field has undergone rapid development [10]. Early research efforts were primarily focused on validating the fundamental photodetection performance of perovskite materials. We have summarized the development trajectory of perovskite PDs, as illustrated in Fig. 1. Since 2015, perovskite PD research has experienced fast-growth phase, with efforts in performance optimization. In terms of device architecture, various configurations such as photoconductive, photovoltaic, and transistor-type structures have been successfully developed, substantially enhancing device performance [11, 12]. Regarding material systems, innovative advancements have been made in lead-free perovskite and its derivative materials, effectively addressing lead toxicity issues while significantly improving the application potential [13, 14]. In the domain of morphology control, controllable preparation of diverse forms has been achieved, including thin films, single crystals (SCs) films, SCs, quantum dots, and micro-/nanostructures, providing new pathways for optimizing device performance [15–17]. Furthermore, strategies such as interface modification and charge transport layer optimization have been developed to enhance device performance [18–20]. During this period, researchers have also employed material engineering approaches to enhance the stability of perovskite materials [21, 22]. Entering the 2020s, research focus on perovskite PDs has shifted toward functional diversification and practical application expansion. Significant developments include various narrowband and broadband detection architectures, multi-pixel visual sensor investigations, and extended applications in optical communications, imaging, and X-ray detection [23–25].

This review provides a systematic analysis of performance enhancement strategies for perovskite PDs, covering past decades of research from the initial exploration to the practical applications. It aims to offer insights for advanced perovskite PD designs for diverse applications from a synergistic perspective of material optimization and device design. It begins by outlining the fundamental working mechanisms and key performance parameters of PDs. Subsequently, the review elaborates on performance optimization strategies centered on material preparation, optical management, interface engineering, and stability improvements, analyzing the associated performance enhancements in perovskite detectors from both theoretical and experimental perspectives. The review further examines representative applications of high-performance perovskite PDs. Finally, it addresses persistent challenges, including device stability and large-area fabrication, and offers perspectives on future research directions for various application scenarios. By consolidating these insights, this work aims not only to summarize existing optimization strategies but also to serve as a roadmap for the continued advancement and practical application of perovskite PDs.

## Structure Classification and Performance Parameters of the PDs

### Classification of PDs

**Fig. 2:**
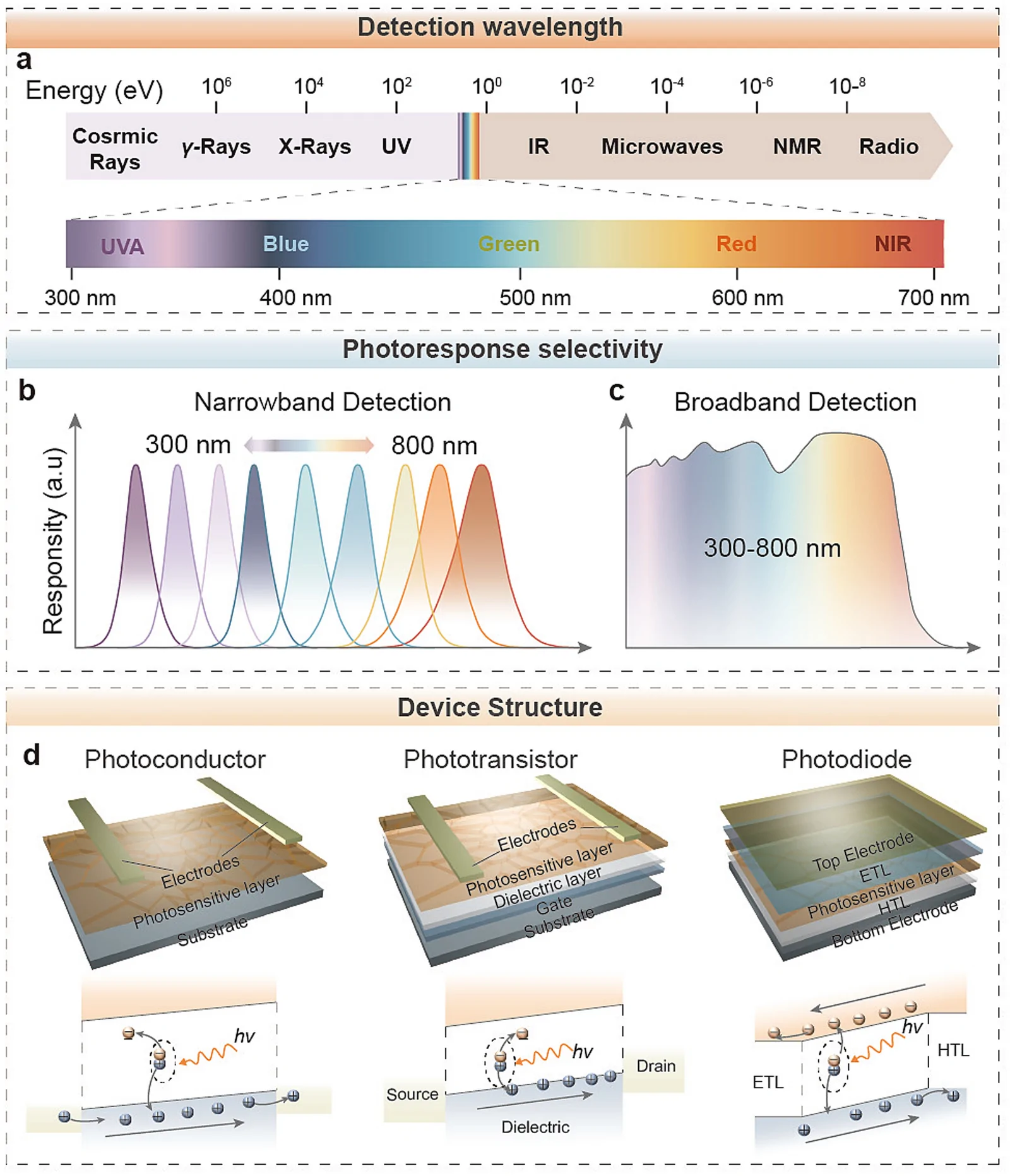
Classification of PDs. **a** Detection wavelength of PDs. **b** and **c** Narrowband and broadband detection of PDs. **d** Device structures of PDs

In general, PDs can be divided into X-ray detectors, UV detectors, visible light detectors, and infrared detectors based on the different detection wavelengths. Different detection bands of PDs meet the application needs of human in medical, industrial, aerospace, daily life, and other aspects (Fig. 2a). According to the spectral response range, the PDs can be divided into narrowband PDs and broadband PDs. As shown in Fig. 2b, narrowband PDs can only sense a small spectral range of light, which makes it have the ability of color recognition. The ability of spectral discrimination for narrowband PDs makes them attractive in many applications including machine vision, biomedical sensing, and imaging [33, 34]. The broadband ones can detect a broad spectral range of light from UV, visible to infrared light (Fig. 2c). A wider detection range is significant to some applications, such as imaging and light communication [35].

From the aspect of working principles and device structures, the PDs can be divided into photoconductors, phototransistors, and photodiodes. As shown in Fig. 2d, photoconductors and phototransistors are lateral devices, while the photodiodes are vertical configurations. In a typical photoconductor, the photosensitive layer acts as a channel between the two electrodes. Under illumination, photogenerated electron–hole pairs in the photosensitive layer can be separated and collected by the two symmetrical electrodes under a bias voltage to realize the conversion of the light signal to the electrical signal. In this process, the external quantum efficiency (EQE) in excess of 100% can be easily obtained. Since electrons and holes have different mobility, in order to keep the photoconductor in a neutral condition, electrons (N-type semiconductors) or holes (P-type semiconductors) can be cycled through the circuit many times, thus resulting in high gain [36]. This high gain mechanism results in high responsivity. However, due to the large electrode spacing of the structure and the long carrier transit time, the photoconductor is usually accompanied by slow response and high driving voltage. For the devices with asymmetrical electrodes, that is, metal–semiconductor–metal (MSM) devices, the carrier driving force can be generated according to the difference work function of metal electrodes.

Phototransistors are three-terminal devices with a lateral structure, composed of gate electrode, dielectric layer, photosensitive layer, and the source/drain electrode. Like photoconductor, the photocarrier generation mechanism is generally based on the photoconductive effect, and the incident light can be used as an additional gate to adjust optoelectronic parameters of the device. Compared with photoconductors, phototransistors can adjust the quantity of charge carriers in the conducting channel by applying the gate voltage under a given source drain voltage, so as to modulate the conducting channel conductivity and suppress the dark current and serve as the electrical signal amplification. Thus, this type of PDs possesses a high photoconductive gain and low noise, resulting in high sensitivity. Similarly, due to the long carrier transit time, the phototransistors suffer from low response speed.

Photodiodes are two-terminal devices in which the photosensitive layer is sandwiched between the top electrode, electron transport layer (ETL), and hole transport layer (HTL), bottom electrode. These vertical devices usually divided into p-n, p-i-n, and Schottky junction structure. The photogenerated electron–hole pairs can be separated by a lower driving voltage or zero bias because of a built­in electric field. At the same time, photodiodes can obtain a low dark current due to the junction barrier, resulting in high specific detectivity. In addition, photodiodes with smaller electrode spacing and shorter carrier transfer distance lead to fast response speed. However, since there is no charge injection at reverse bias voltages with rectified contacts, the photodiode is lack of an internal gain mechanism result in EQE below 100% and low responsivity.

Perovskite materials exhibit high compatibility with three types of PD structures, yet each configuration presents unique performance emphases and technical challenges. Among them, photoconductors feature a simple structure and high gain but suffer from large dark current and poor stability, requiring optimization through materials engineering and structural design. Phototransistors combine high gain with tunability, making them an important direction for future intelligent sensing. However, ion migration in perovskites can severely interfere with field-effect modulation, necessitating strategies such as heterogeneous integration and materials engineering. Notably, perovskite-based planar PDs exhibit greater tolerance for material configurations, allowing perovskites of different dimensionalities to be used for high-performance devices. For photodiodes, they enable self-powered operation, high sensitivity, and fast response, with performance heavily dependent on high-quality thin-film crystallization and interface engineering. In the case of vertical photodiodes, charge transport must occur along the film thickness direction, making two-dimensional (2D) perovskite materials with long-chain organic cations generally unsuitable for such structures due to their poor out-of-plane conductivity.

Furthermore, in terms of process compatibility, planar perovskite photodiodes can circumvent incompatibility issues with conventional CMOS processes through methods such as inverted electrode design and dry transfer, whereas the stacked structure of vertical photodiodes requires further exploration of CMOS-compatible processes.

In summary, perovskites offer broad possibilities for low-cost, high-performance photodetection, but different device structures demand distinct optimization strategies. Photoconductors require excellent thin-film crystallization quality to reduce defects and enhance carrier transport speed. Photodiodes need to balance optical management, interface engineering, and suitable fabrication processes to improve light utilization, achieve efficient carrier collection, and enable large-area manufacturing. Phototransistors, with their three-terminal design, can balance the advantages of the former two through gate voltage control, but their complex structure imposes more stringent requirements on bandgap tuning and heterojunction interface design.

### Key Parameters of PDs

To evaluate the performance PDs, several parameters for practical characterization are summarized, including on/off ratio, responsivity (*R*), specific detectivity (D^*^), *EQE*, response time, linear dynamic range (LDR), photoconductive gain.

1. *On/off ratio*: It refers to the ratio of photocurrent and dark current. The value is mainly dependent on the photoelectric conversion and charge transport ability of the active layer.
2. *R*: R is defined as the generated photocurrent per unit power of the incident light on the effective area, representing the optoelectronic conversion ability of a PD, which are usually expressed by the following equation:

1 $$R = \frac{{I_{p} - I_{d} }}{{P_{{{\mathrm{in}}}} }}$$

where* I*_p_ and *I*_d_ are the photocurrent and dark current, respectively, *P*_in_ is the incident light intensity of the effective illuminated area.

3. * EQE*: It is defined as the ratio between the number of photogenerated electron–hole pairs and the incident photons per unit time. EQE can be expressed as:

2 $${\mathrm{EQE}} = \frac{{I_{p} /e}}{{P_{{{\mathrm{in}}}} /h\nu }} = \frac{hc}{{e\lambda }} \cdot R$$

where *e* is the elementary charge, *h* and *c* are the Planck’s constant and light speed, *ν* is the frequency of incident light, and *λ* is the wavelength of incident light.

4. D^*^: It represents the weakest level of light that the device can detect. D^*^ is determined by the responsivity and noise of a PD, so it is defined as:

3 $$D^{*} = \frac{{(A\Delta f)^{1/2} \cdot R}}{{i_{n} }} = \frac{{(A\Delta f)^{1/2} }}{{{\mathrm{NEP}}}}$$

4 $${\mathrm{NEP}} = \frac{{i_{n} }}{R}$$

where *A* is the effective area of a detector, Δ*f* is the electrical bandwidth, and *i*_n_ is the noise current. NEP is the noise equivalent power. It is defined as the optical input power required to achieve a unity signal-to-noise ratio, representing the minimum detectable light intensity that the device can distinguish from the total noise.

5. * Response time*: The rise and fall time (*τ*_rise_ and *τ*_decay_) of current are defined as rising and decay time from 10 to 90% or 90% to 10% of the maximum current. The other method involves measuring the 3 dB bandwidth, which is defined as the frequency at which the *R* decreases to half of its maximum value under continuous illumination. This can be expressed by the following formula:

5 $$f _{{ - 3{\mathrm{dB}}}}^{ - 2} = \left( {\frac{3.5}{{2\pi t_{tr} }}} \right)^{ - 2} + \left( {\frac{1}{{2\pi {\mathrm{RC}}}}} \right)^{ - 2}$$

where ${t}_{tr}$ is the carrier transit time and RC is the resistance–capacitance time constant of the circuit.

6. * LDR*: It represents the range over which the *I*_p_ is linear to the incident light intensity. It was estimated by using the following equation:

6 $${\text{LDR = 20log}}\frac{{P_{{{\mathrm{max}}}} }}{{P_{{{\mathrm{min}}}} }}$$

where *P*_max_ and* P*_min_ are the maximum and minimum detectable light densities in the linear range, respectively.

## Basic Characteristics of Perovskite Materials

### Crystal Structure and Chemical Composition

Typical organic–inorganic and all-inorganic metal halide perovskites adopt the general chemical formula ABX_3_, where A denotes monovalent cation (CH_3_NH_3_^+^, CH(NH_2_)_2_^+^, Cs^+^), B is the Pb^2+^, and X is a halide anion (Cl^−^, Br^−^, I^−^) [43]. In general, the cation B (at the cube center) has six nearest anions X (at the cube face center), which can be expressed as [BX_6_]^4−^. Its most distinctive feature is that the [BX_6_]^4−^ octahedra are connected by sharing vertices, extending infinitely along the X, Y, and Z directions to form a stable three-dimensional rigid framework. Smaller A-site cations (such as MA⁺ and Cs⁺) fill the cavities within the octahedral framework, balancing the charge and stabilizing the structure. The A, B, and X sites exhibit diverse choices, and the ion size ratios that can maintain the perovskite crystal structure are described by the Goldschmidt tolerance factor (*τ*) and the octahedral factor *μ* [44]:

7 $$t = \frac{{r_{A} + r_{x} }}{{\sqrt 2 (r_{A} + r_{x} )}}$$

8 $$m = \frac{{r_{B} }}{{r_{x} }}$$

**Fig. 3:**
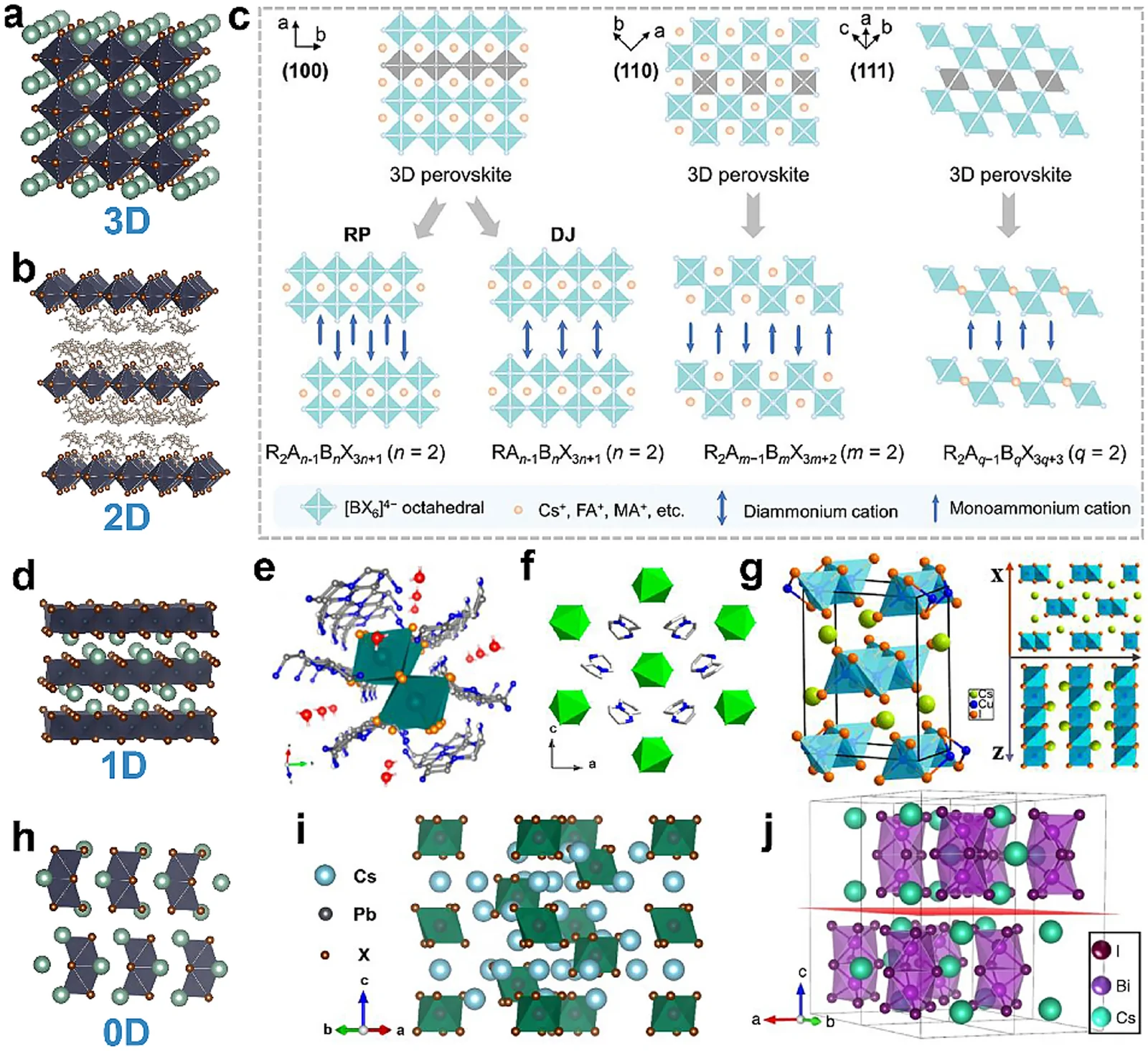
Structure of various low-dimensional Pb-based perovskites. **a** Crystal structure of 3D perovskites. **b** Crystal structure of 2D perovskites. **c** Schematic illustration of the transformation from various 3D perovskites to 2D perovskite crystal structures. Reproduced with permission from Ref. [37] Copyright 2025, Wiley. **d** Crystal structure of 1D perovskites. Representative crystal structures of 1D perovskites include **e** (H_2_O)(C_6_H_8_N_3_)_2_Pb_2_Br_10_, Reproduced with permission from Ref. [38] Copyright 2019, ACS. **f** CsCu_2_I_3_ Reproduced with permission from Ref. [39] Copyright 2020, RSC. and **g** [DBN]PbCl_3._ Reproduced with permission from Ref. [40] Copyright 2020, ACS. **h** Crystal structure of 0D perovskites. Representative crystal structures of 0D perovskites include **i** Cs_4_PbBr_6,_ Reproduced with permission from Ref. [41] Copyright 2024, Wiley. and **j** Cs_3_Bi_2_I_9_. Reproduced with permission from Ref. [42] Copyright 2020, Springer

In the formula, *r*_A_, *r*_*X*_, and *r*_*B*_ correspond to the ionic radii of the A-site, X-site, and B-site, respectively. Typically, when the *τ* value is between 0.813 and 1.107, and simultaneously the octahedral factor *μ* satisfies 0.44 < *μ* < 0.90, ABX_3_ can form a stable 3D perovskite phase (Fig. 3a).

The radius of the A-site cation has a decisive influence on the structural stability of perovskite materials. A radius that is too small (e.g., Na⁺) will induce octahedral distortion, while a radius that is too large (e.g., imidazole) will disrupt the three-dimensional network and drive the material to transform into a 2D layered structure [44]. 2D perovskites can be regarded as structures formed by the partial or complete substitution of A-site cations in 3D perovskites with large-sized spacer cations, as shown in Fig. 3b. Due to the A-site cations being too large to be accommodated in the perovskite lattice, the continuous perovskite octahedral network is cut apart, forming layered perovskites. The spatial motion of electrons is confined in the dimension orthogonal to the “cut surfaces” but unrestricted in the other two dimensions. Depending on the crystallographic orientation along which the octahedra are cut, two-dimensional layered perovskites can exhibit different structural characteristics (Fig. 3c): The < 100 > orientation gives rise to the R_2_A_n-1_B_n_X_3n+1_ perovskite structure, the < 110 > orientation leads to the R_2_A_m-1_B_m_X_3m+2_ perovskite structure, and the < 111 > orientation results in the R_2_A_q-1_B_q_X_3q+1_ perovskite structure [37]. Here, R refers to the spacer cation, and n, m, and q represent the number of layers of [BX_6_]^4−^ octahedral units. Layered perovskites oriented along the < 100 > direction mainly include the Ruddlesden–Popper (RP) phase and the Dion–Jacobson (DJ) phase, with the difference between the two lying in the type of spacer cation and the interconnection mode between the perovskite layers. In the RP phase, monoammonium cations (such as isopropylammonium IPA⁺, phenethylammonium PEA⁺, etc.) are used as organic spacer cations, and the perovskite octahedra are bonded together by van der Waals forces. Due to steric hindrance, the [BX_6_]^4−^octahedral layers form a staggered configuration with a (1/2, 1/2) displacement on the *ab* plane. In the DJ-phase perovskites, the organic spacer cations are diammonium cations, such as butyl diammonium (BDA⁺). The perovskite octahedra are directly connected through spacer cations, with the inorganic octahedral layers arranged neatly and without relative displacement.

1D perovskites consist of highly distorted single-layer parallel octahedral structures [BX_6_]^4−^and large organic cations (Fig. 3d). In one-dimensional chain-like perovskites, the connection modes between octahedra exhibit diversity, bringing more possibilities for the synthesis of perovskites or perovskite derivatives. An example is (H_2_O)(C_6_H_8_N_3_)_2_Pb_2_Br_10_, which adopts a corner-sharing connection mode [38], CsCu_2_I_3_ adopts a zigzag edge-sharing connection [39], [DBN]PbCl_3_ adopts a face-sharing connection [40], and the crystal structures are shown in Fig. 3e−g, respectively. Due to the unique structure of 1D perovskites, the octahedral chains are isolated by the surrounding rigid organic ammonium groups, which confines the motion of charge carriers to one dimension, resulting in higher exciton binding energies and significant quantum confinement.

0D-type perovskites are composed of isolated octahedral networks and cations, as shown in Fig. 3h. In the zero-dimensional structure, the [BX_6_]^4−^octahedra are completely isolated from each other, typically surrounded by organic or inorganic A-site cations, and exist as dispersed monomers or face-sharing dimers (finite small clusters). Examples include Cs_4_PbBr_6_ (Fig. 3i) [41] and Cs_3_Bi_2_I_9_ (Fig. 3j), which form finite dimeric clusters via face sharing [42]. Since there is no direct connectivity between these isolated octahedra, their structural stability depends more on the octahedral factor than on the traditional tolerance factor, thereby expanding the range of choices for B-site metal cations.

**Fig. 4:**
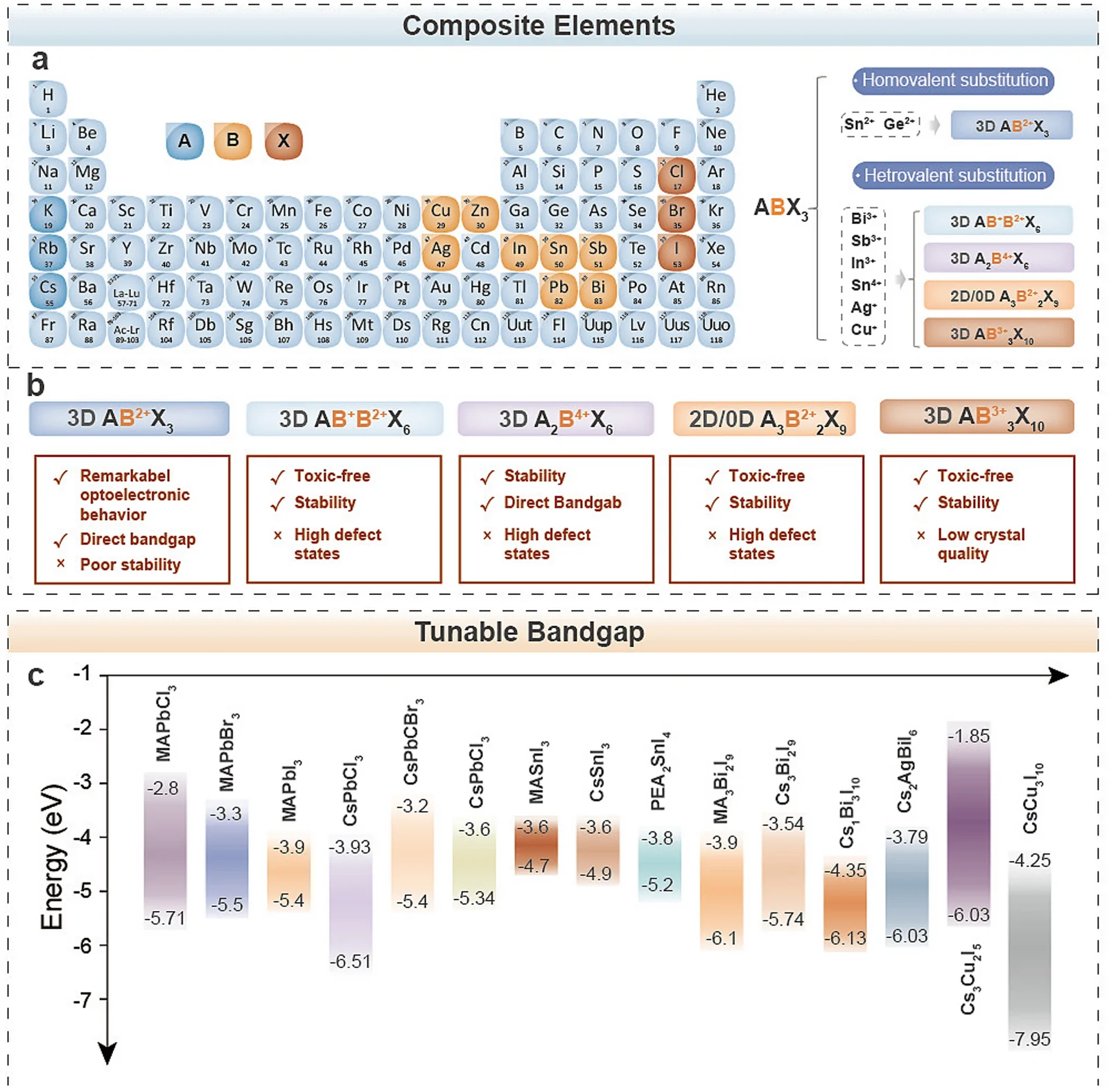
Classification of perovskites structures and their corresponding optoelectronic properties. **a** Candidates for replacing Pb from the periodic table of elements and design principle for lead-free perovskite materials. **b** Corresponding properties of various lead-free perovskite materials. **c** Band structures of common perovskite materials

Pb^2+^ halide perovskites have excellent optoelectronic properties, including direct band gap [45], large light absorption coefficient (10^5^ cm^−1^) [46], suitable adjustable band gap [47], long photogenerated carrier lifetimes (1 μs) [48], long diffusion length and high defect tolerance [49, 50]. These remarkable optoelectronic properties are derived from the combination of high perovskite symmetry (*O*_h_), high electronic dimensionality, and the unique atomic electronic configuration of Pb [51]. However, the toxicity of Pb element is harmful to human and environment, which will hinder further deployment and commercialization. In many countries, there are regulations restricting the use of harmful heavy metals (including Pb) in electronic devices. To get rid of the Pb but keep the optimal properties of the material, B-site doping by nontoxic elements could provide a solution. In order to retain the advantages of Pb-based perovskite materials, the B-site replacement material should conform to the electronic configuration of a typical Pb-based perovskite, where the valence band consists of the p-orbitals of halide and s-orbitals of Pb, while the conduction band is only consisted of the p-orbitals of the Pb [52]. Based on these, the elements following the order of Pb electronic configuration are preferred as potential alternatives (Fig. 4a). Thus, some homovalent (such as Sn(II), Ge(II), and Cu(II)) or heterovalent elements (such as Sn(IV), Sb, Bi(III), Sb(III), In(III), Ag(I), and Cu(I)) have been employed as the replacements of Pb.

Some materials exhibit remarkable optoelectronic properties due to lattice distortion and deformation caused by B-site substitution, comparable to or even superior to Pb-based perovskites in some respects (Fig. 4b). Specifically, only Sn(II) and Ge(II) meet the preconditions of coordination and charge balance, avoiding obvious lattice perturbations caused by substitution, so that traditional perovskite structures can be formed [51]. At the same time, due to the same lone-pair *s*-orbitals, the Sn-/Ge-based perovskites have comparable optoelectronic properties to the Pb-based perovskites. However, the applications of ASn(II)X_3_ perovskites in optoelectronic devices still have the problem of instability and high density Sn vacancy, which leads to serious device degradation [53]. When stable Sn(IV) or Ge(IV) is used as a substitute element, one Sn(IV) or Ge(IV) ion and a vacancy replace two Pb(II) ions in APb(II)X_3_ perovskites to form vacancy-ordered perovskites. The structure is composed of a face-centered lattice of SnX_6_ units with A cations occupying the cuboctahedral voids.

Trivalent Bi^3+^ and Sb^3+^ ions possess *ns*^2^ electronic configurations and ionic radii similar to those of Pb^2+^, endowing them with notable optoelectronic performance. First, their similar *ns*^2^ electronic configuration with Pb promotes an anti-bonding interaction at the valence band maximum (VBM), confining defect states to shallow levels near the band edges. As a result, even in the presence of defects, the optoelectronic characteristics of B(III) cations can be largely preserved. Second, Bi^3+^ and Sb^3+^ exhibit high absorption coefficient, comparable to those of Pb-based materials, which originate from transitions between X-5*p* valence states and 6*p* conduction states [54]. Moreover, the optical transition from 6* s* states (near the VBM) to 6*p* conduction states further enhances the high absorption coefficient. Third, the octahedrally coordinated structure of Bi/Sb-based perovskites favors an extended carrier diffusion lifetime. These remarkable properties position Bi- and Sb-based materials as promising, environmentally benign alternatives to toxic Pb [55]. For such heterovalent substitutions, the chemical formula is A_3_B(III)_2_X_9_, which is equivalent to that obtained by removing 1/3 of the B-site cations from ABX_3_. The A_3_B_2_X_9_ compounds have two typical structures: One is 0D dimer form composed of isolated face-sharing octahedra [B_2_X_9_]^3−^, and the other is the 2D layered perovskite. The preferential formation of two competing structures depends on the selection of A, B, and X ions. Additionally, halide double perovskites with B-site substitution by mixed cations (B = B(I), B’ = B(III)) have been explored to form 3D A_2_BB’X_6_ structure. The crystal unit of halide double perovskites is like AB(II)X_3_, and the difference is that the B-site cations are alternately arranged by mixed cations (B = B(I), B’ = B(III)) to occupy octahedral center. The wide range of different elements are available for B(I) and B(III) ions, providing tremendous options. In addition, Cu(I) and In(III) are also candidates for B-site substitution. Due to their smaller ion radius than that of Pb cation, low-dimensional perovskite derivatives are formed. These halide semiconductors have a completely different crystal structure from AB(II)X_3_ and are often referred to as perovskite derivatives, with superior stability and a direct band gap [39].

### Photoelectric Properties

Excellent light absorption capability is one of the core advantages of perovskite materials. This strong absorption characteristic stems from the direct bandgap properties of perovskites and the allowed dipole transitions between the valence and conduction bands [56]. These materials exhibit an extremely high absorption coefficient (approximately 10^5^ cm⁻^1^) in the visible spectrum, representing an order of magnitude improvement over conventional Si and GaAs materials [51]. This remarkable property enables halide perovskites to achieve > 90% light absorption with absorber layers as thin as ~ 1 μm, facilitating exceptional photoelectric conversion efficiency in ultrathin device architectures. This property offers significant benefits for developing next-generation wearable devices with high integration density.

The carrier transport performance directly affects the efficiency of optoelectronic devices. Owing to the low effective mass of charge carriers in perovskite materials, their enhanced mobility contributes to significantly longer diffusion lengths. Research has demonstrated that perovskites exhibit exceptionally long carrier diffusion lengths of (reaching several micrometers), facilitating the effective extraction of photogenerated electron–hole pairs prior to recombination [49]. Additionally, perovskite materials exhibit balanced bipolar transport characteristics, with both electron and hole mobilities reaching 10 − 100 cm^2^ V^−1^ s^−1^, significantly higher than those of organic semiconductor materials and comparable to some inorganic semiconductors [57].

The tunable bandgap structure offers significant potential for application-specific optimization. By adjusting the halide composition (Cl^−^, Br^−^, I^−^), the optical bandgap of perovskites can be precisely modulated across a broad range of 1.2 − 3.0 eV, corresponding to absorption edges spanning from near-infrared (NIR) to UV regions. This unique characteristic enables tailored material design for diverse applications, including both narrowband and broadband detection. Furthermore, through B-site substitution strategies, the bandgap of conventional Pb-based perovskites can be extended into the deep UV spectrum, substantially expanding their application potential. Figure 4c summarizes the band structures of different perovskites materials. It can be seen that the bandgap energies of perovskite materials vary significantly with composition, accompanied by substantial fluctuations in the conduction band minimum (CBM) and VBM. These parameters form the essential basis for structural design and device performance optimization.

### Challenges in Perovskite Stability

Perovskite materials demonstrate exceptional optoelectronic performance for various applications, including photovoltaics, light-emitting devices, and PDs. However, their stability issues present significant obstacles to commercial viability. The main stability issues include moisture stability, thermal stability, and photostability [58].

Firstly, perovskite materials exhibit extreme sensitivity to moisture owing to their ionic nature, making them prone to rapid degradation [59]. The decomposition mechanisms primarily involve hydrolysis reactions and phase separation. Taking MAPbI_3_ as an example, water molecules can penetrate the perovskite lattice on a timescale of seconds, leading to the dissociation of MA⁺ from the Pb − I framework and the formation of PbI_2_ and HI or CH_3_NH_2_. In addition, moisture exposure induces the migration of halide anions (I⁻/Br⁻), resulting in phase segregation and deteriorated optoelectronic properties [60].

Thermal instability in perovskite materials manifests as phase transitions or structural decomposition at high temperatures, leading to the subsequent degradation of MA⁺ cations [61]. The probable decomposition mechanism proceeds as follows [58]:

9 $${\mathrm{CH}}_{{3}} {\mathrm{NH}}_{{3}} {\mathrm{PbI}}_{{3}} \rightarrow {\mathrm{PbI}}_{{2}} \left( {\mathrm{s}} \right) + {\mathrm{CH}}_{{3}} {\mathrm{NH}}_{{2}} \left( {\mathrm{g}} \right) + {\mathrm{HI}}\left( {\mathrm{g}} \right)$$

10 $${\mathrm{CH}}_{{3}} {\mathrm{NH}}_{{3}} {\mathrm{PbI}}_{{3}} \rightarrow {\mathrm{PbI}}_{{2}} \left( {\mathrm{s}} \right) + {\mathrm{CH}}_{{3}} {\text{I }}\left( {\mathrm{g}} \right) + {\mathrm{NI}}_{{3}} \left( {\mathrm{g}} \right)$$

Moreover, thermal stress promotes the generation of halogen vacancies, thereby enhancing ion migration.

The photochemical instability of perovskites manifests through two primary mechanisms under UV exposure. On the one hand, the light-induced mobility of halide anions (I⁻/Br⁻) initiates phase separation. On the other hand, photogenerated reactive oxygen species degrade the crystalline framework via these reaction steps [62]:

11 $${\mathrm{CH}}_{{3}} {\mathrm{NH}}_{{3}} {\mathrm{PbI}}_{{3}} + {\mathrm{h}}\nu \rightarrow {\mathrm{CH}}_{{3}} {\mathrm{NH}}_{{3}} {\mathrm{PbI}}_{{3}}^{*}$$

12 $${\mathrm{CH}}_{{3}} {\mathrm{NH}}_{{3}} {\mathrm{PbI}}_{{3}}^{*} + {\mathrm{O}}_{{2}} \rightarrow {\mathrm{CH}}_{{3}} {\mathrm{NH}}_{{3}} {\mathrm{PbI}}_{{3}} + {\text{ O}}_{{2}}^{ - }$$

13 $${\mathrm{4CH}}_{{3}} {\mathrm{NH}}_{{3}} {\mathrm{PbI}}_{{3}}^{*} + {\text{ O}}_{{2}}^{ - } \rightarrow {\mathrm{4CH}}_{{3}} {\mathrm{NH}}_{{2}} + {\mathrm{4PbI}}_{{2}} + {\mathrm{2H}}_{{2}} {\mathrm{O}}$$

Given the susceptibility of perovskite materials to environmental factors such as humidity, illumination, and thermal stress, significant efforts have been devoted to improving their stability via material engineering [63], additive optimization [22], and device architecture design [64], thereby advancing their commercialization in optoelectronic devices.

## Material Quality Control Engineering for High-Performance Perovskite PDs

Perovskite materials possess excellent photoelectric properties and relatively poor stability. To maximize the advantages of perovskite while enhancing its stability, a series of multidimensional material preparation methods have been developed.

**Fig. 5:**
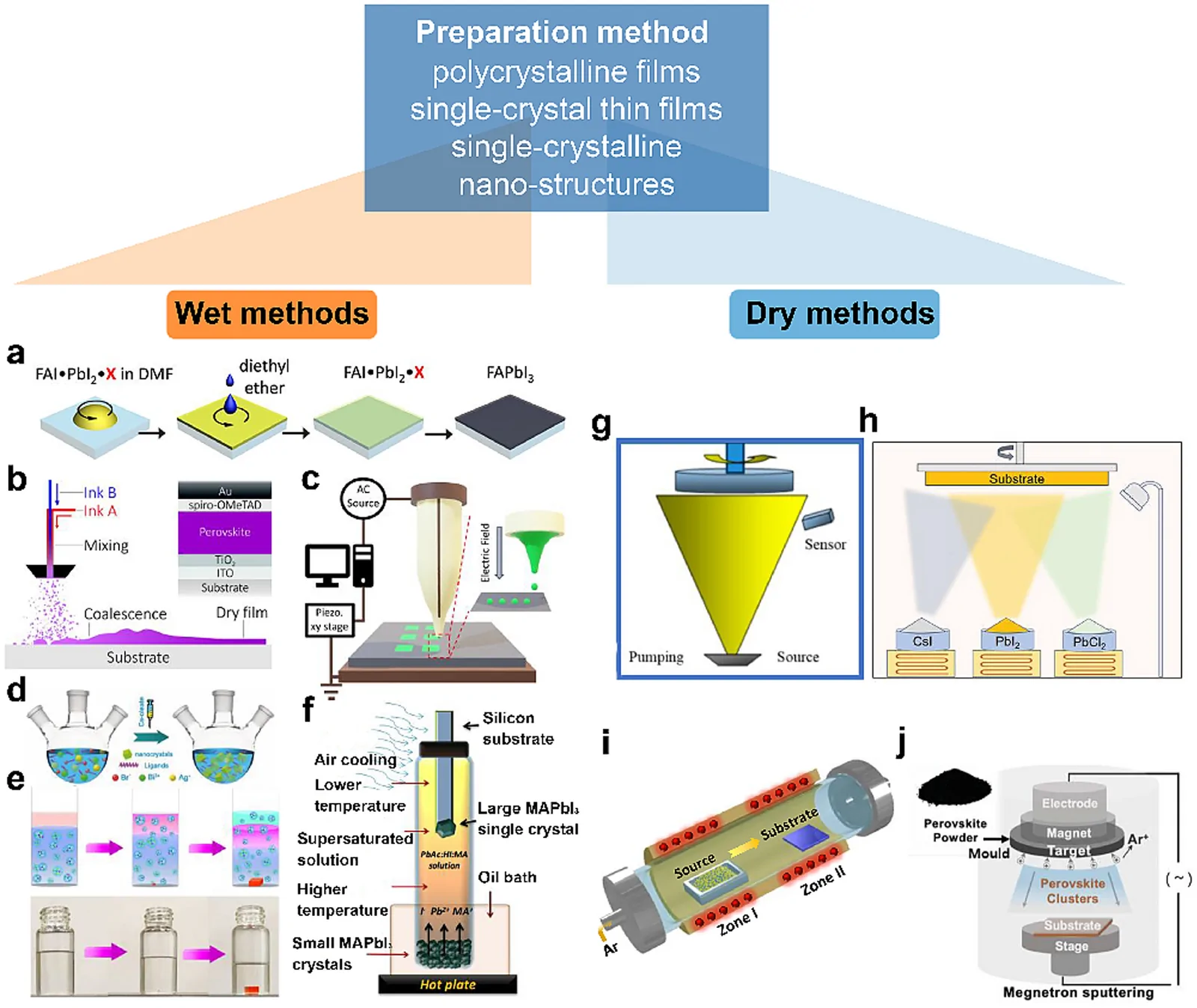
Different preparation methods of perovskite materials. Schematic diagram of the formation of perovskite films by **a** spin coating method. Reproduced with permission from Ref. [65] Copyright 2018, American Chemical Society; **b** Concurrently pumped ultrasonic spray coating method. Reproduced with permission from Ref. [66] Copyright 2016, Royal Society of Chemistry; **c** Inkjet printing. Reproduced with permission from Ref. [67] Copyright 2022, American Chemical Society. **d** Schematic illustration of the solution phase synthesis of perovskites nanocrystals or quantum dots by hot injection route. Reproduced with permission from Ref. [68] Copyright 2018, Wiley. Schematic illustration of growth diagram of perovskite SCs by **e** room-temperature liquid diffused separation induced crystallization method, Reproduced with permission from Ref. [69] Copyright 2020, Springer Nature; **f** Top-seeded solution growth method. Reproduced with permission from Ref. [49] Copyright 2015, AAAS. Schematic diagram of the formation of perovskite films or micro-/nanostructures by **g** single-source coevaporation method, Reproduced with permission from Ref. [70] Copyright 2016, Springer Nature; **h** Multiple-source coevaporation method, Reproduced with permission from Ref. [71] Copyright 2023, Wiley; **i** Chemical vapor deposition method. Reproduced with permission from Ref. [72] Copyright 2018, American Chemical Society; **j** Single-step sputtering method. Reproduced with permission from Ref. [73] Copyright 2021, Wiley

This section begins with an overview of traditional preparation methods for perovskite materials and then extends the discussion to recent advances in multidimensional synthesis techniques for enhancing device performance, including polycrystalline films, single-crystal thin films (SCTFs), single-crystalline, micro-/nanostructure and their arrays, oriented nanostructures. The traditional fabrication methods can be divided into wet methods and dry methods (Fig. 5).

### Wet Methods

Specifically, the wet methods rely on chemical reactions and molecular assembly in a liquid environment, and precursor solutions need to be prepared before the subsequent treatment. Typical wet preparation methods include spin coating, spray coating, drop casting, inkjet printing, and hot injection, etc.

#### Spin Coating

As shown in Fig. 5a, one-step spin coating is one of the most typical methods to prepare polycrystalline thin film because of its simplification and low cost, in which a mixture of precursors was dissolved in solvents and the crystalline absorber is formed by spin coating. During this process, many strategies are used to improve the quality of the polycrystalline films, such as adding antisolvent and forming Lewis acid–base adducts. The spinning coating method has significant advantages in the preparation of large-area thin films.

#### Spray Coating

In order to prepare the films at specific locations, spray and inkjet printing methods are proposed. As shown in Fig. 5b, micron-sized aerosol droplets were generated through the mega sonic nebulizer source; then, the aerosol is mixed with the carrier gas (such as inert gas) controlled by the mass flow controller and sprayed through the nozzle. The nozzle and substrates can be moved by motion stages. Thus, the spraying operation can be precisely controlled for a consistent and reliable coating.

#### Inkjet Printing

Figure 5c shows a schematic of the typical inkjet printing setup. A voltage is applied between the electrode inside the print nozzle and the substrate to perform the printing. This voltage polarizes the printing solution, creating an electric field that overcomes the intrinsic surface energy controlling the meniscus curvature of the ink at the nozzle orifice. Droplet formation and acceleration to the substrate can be controlled by changing the voltage, and the controlled movement of the substrate stage enables the printing of high-resolution patterns.

#### Preparation Techniques for Quantum Dots (QDs)

Perovskite QDs can be prepared by ligand-assisted reprecipitation and hot injection. The ligand-assisted reprecipitation method is a process in which the prepared precursor solution is quickly injected into an antisolvent environment, and the concentration of the precursor solution is quickly saturated to obtain crystallization products. Then, a homogenized QDs or nanocrystals (NCs) are obtained by centrifugation process. In a typical hot injection method, a source of A-site ions is injected into a high-boiling solution containing B-site ions and halogen ions. After cooling rapidly, perovskite QDs or NCs are obtained, as shown in Fig. 5d.

#### Preparation Techniques for Single Crystals

In addition, in order to obtain high-quality perovskite SCs, a variety of methods for preparing SCs have been developed, including antisolvent vapor-assisted crystallization, temperature-lowering crystallization, slow evaporation of the solution crystallization, nucleation-controlled growth, top-seeded solution growth, room-temperature liquid diffused separation induced crystallization, and low-temperature-gradient crystallization. The key of the above method is to change the unsaturated solution into a saturated solution by changing the temperature or changing the solubility of the solvent, causing the crystal to nucleate and grow. For example, Fig. 5e shows the schematic illustration of room-temperature liquid diffused separation induced crystallization methods. As the solvent from the precursor solution diffuses spontaneously into the silicone oil, the solution becomes oversaturated, leading to the progressive crystallization of perovskite SCs. At the same time, the thickness of crystals can be controlled by tuning the height of the precursor solution from microns to millimeters. Figure 5f presents the process of top-seeded solution growth method. The precursor solution is cooled to induce saturation, concomitant with the nucleation of small SCs. The left clear solution is carefully decanted into a closed bottle to function as the growth medium for larger SCs. These seed crystals are then deposited at the base of the bottle, followed by insertion of a silicon substrate. The air-cooled silicon substrate positioned in the upper chamber significantly enhanced latent heat dissipation, thereby promoting controlled SCs nucleation and the subsequent formation of larger SCs.

### Dry Methods

Dry preparation methods focus on direct reactions in the gas phase or solid state, offering distinct advantages in terms of precise control over material morphology, thickness, and uniformity. Representative techniques include vacuum deposition and chemical vapor deposition. These methods exhibit significant potential for industrial applications, characterized by their high efficiency, high quality, and suitability for large-scale production.

#### Vacuum Deposition

Vacuum deposition has emerged as a widely adopted deposition technique for perovskite films fabrication. The film quality is critically influenced by multiple parameters, including chamber vacuum pressure, crucible heating power, and the quantity and spatial distribution of source materials within the crucible. To date, two principal evaporation methodologies have been developed, single-source coevaporation method, and multiple-source coevaporation method. Single-source coevaporation involves the simultaneous vapor deposition of multiple precursor materials mixed at predetermined stoichiometric ratios (Fig. 5g). In this process, the precursors form a liquid-phase molten salt at temperatures significantly below the melting points of constituent metal halides, thereby enabling controlled evaporation of the mixture with precisely tunable composition and film thickness [71]. Figure 5h schematically illustrates the multi-source coevaporation method. In this approach, precursor materials are loaded into separate crucibles and simultaneously evaporated by individually controlling their heating temperatures according to their distinct melting points. While the above single-step coevaporation techniques have demonstrated promising results, the random diffusion of molecules within the vacuum chamber poses challenges for real-time monitoring and precise control of perovskite film deposition rates [74]. To address this limitation, a multiple-step sequential evaporation method has also been developed, involving layer-by-layer deposition of source materials followed by controlled crystallization.

#### Chemical Vapor Deposition

Another representative dry method is chemical vapor deposition, which offers distinct advantages in precisely controlling the composition and stoichiometry of perovskite materials. Additionally, chemical vapor deposition facilitates the deposition of perovskite films on a wide range of substrates, including flexible and non-planar surfaces. Figure 5i shows a typical diagram of the double-zone chemical vapor deposition methods. The perovskite precursors are premixed and loaded into the high-temperature zone, while the substrate is placed in the low-temperature zone. The vaporized precursors are transported by carrier gas to the deposition zone, where they undergo controlled crystallization on the substrate to form perovskite films or micro-/nanostructure materials.

#### Sputtering Deposition

Sputtering deposition of perovskite materials offers the distinct advantage of precise control over film thickness and composition through manipulation of sputtering parameters, as schematically illustrated in Fig. 5j. Similar to vapor deposition methods, this approach accommodates various substrate geometries including flexible and non-planar configurations, and it can operate at relatively low temperatures. The sputtering process exhibits exceptional scalability for industrial-scale manufacturing, with demonstrated capability for meter-scale uniform deposition. Its superior material utilization efficiency provides distinct economic advantages over thermal evaporation methods.

### Space-Limiting Methods for Material Improvement

**Fig. 6:**
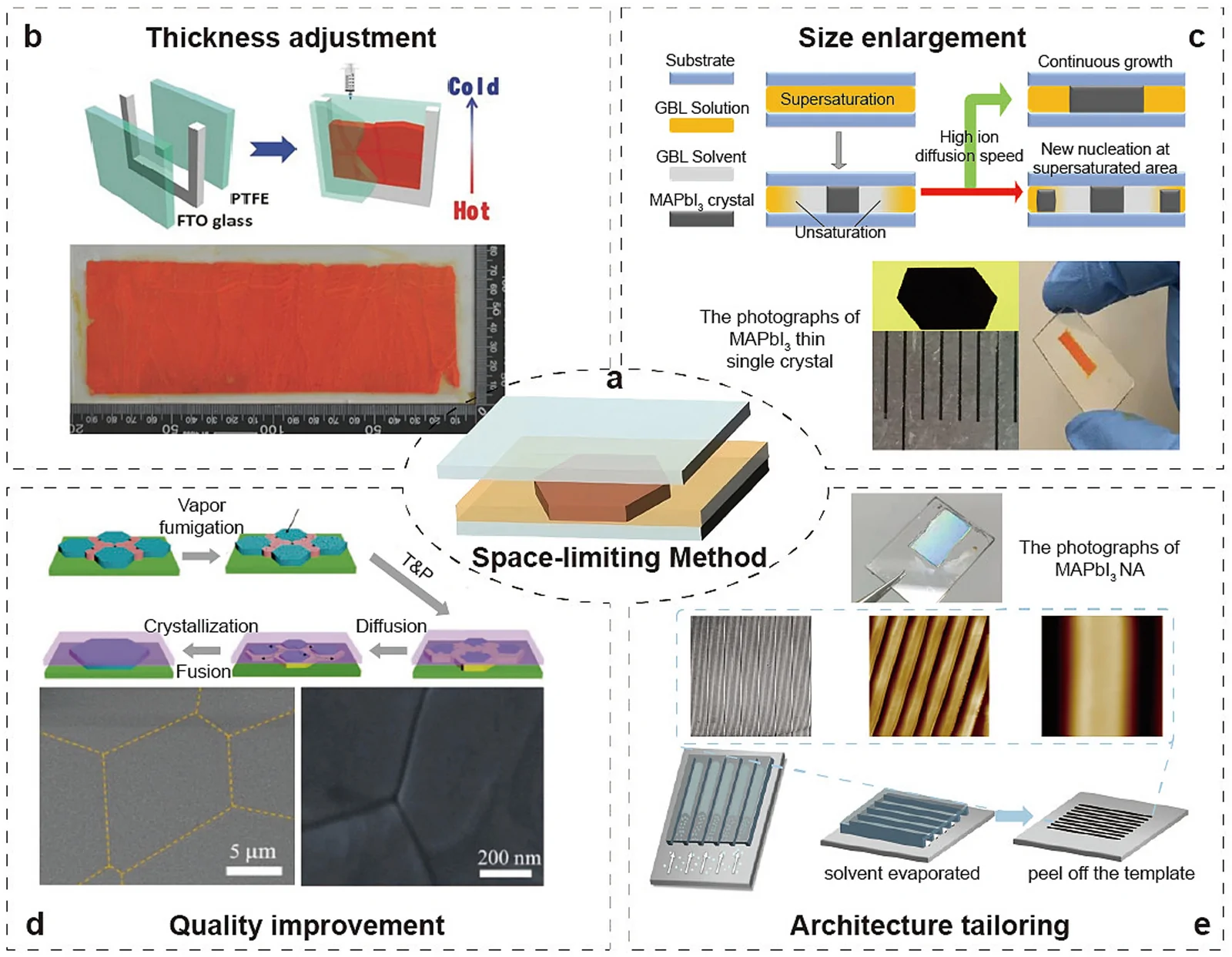
Advancements in perovskite growth through spatial confinement techniques. **a** Schematic diagram of space confinement method. Reproduced with permission from Ref. [75] Copyright 2021, Springer Nature. **b** Schematic diagram of the module to grow laminar CH_3_NH_3_PbBr_3_ crystal films, whose thickness was defined by polytetra-fluoroethylene (PTFE) thin membranes’ thickness. Reproduced with permission from Ref. [79] Copyright 2017, Wiley. **c** Schematic illustrations of correlation between ion diffusion and thin single-crystal growth. Reproduced with permission from Ref. [80] Copyright 2017, Springer Nature. **d** Schematic illustration of the growth mechanism of the MAPbI_3_ film by the space-confined solvent engineering strategy. Reproduced with permission from Ref. [81] Copyright 2018, American Chemical Society. **e** Nanowire growth process by capillary condensation containing solution infiltration, solvent evaporation, and remove template. Reproduced with permission from Ref. [82], Copyright 2025, Springer Nature

The perovskite polycrystalline films, SCs, and nanostructures fabricated using the above methods have been widely used in the field of photodetection. To simultaneously improve the optoelectronic performance and stability of perovskite PDs while broadening their application scope, space-limiting methods have been developed. Figure 6a presents the commonly approach for preparing SCTFs without grain boundary, namely space-confined crystallization technique [75]. This approach achieves controlled directional growth of SCTFs through the application of external forces. The standard experimental method involves depositing the precursor solution between two substrates with opposing wettability characteristics (a hydrophilic and a hydrophobic substrate). Upon application of appropriate physical pressure, high-quality SCTFs can be obtained on the hydrophilic substrate surface. It should be particularly noted that this spatial confinement strategy can be implemented through multiple approaches [76–78]. In addition to the aforementioned physical template confinement method, electrostatic interactions or van der Waals forces can also effectively regulate the directional crystallization process of the SCTFs. By fine-tuning the space-limited method, material growth quality can be enhanced in multiple aspects, including controllable thickness, increased dimensions, improved quality, and tailored architecture.

#### Thickness Adjustment

The preparation of perovskite films necessitates breaking away from isotropic growth conditions, thereby requiring the establishment of an anisotropic growth environment. To this end, the space-limiting methods (Fig. 6a), which possess the capability for controllable adjustment of crystal thickness, have garnered significant attention. This approach achieves precise regulation of perovskite film thickness by restricting the vertical growth space of the crystal. By precisely controlling the inter-substrate spacing, thickness-tunable SCTFs can be fabricated via the spatially confined growth method. Figure 6b illustrates a sandwich-type assembly model for perovskite crystalline film growth, where the thickness of the U-shaped PTFE spacer determines the resulting perovskite film thickness. The confined growth space is constructed by clamping FTO glass with a U-shaped PTFE placed in the middle. Subsequently, the perovskite precursor solution is injected into this spatially confined cavity, with residual bubbles being removed through vacuum pumping. To obtain dense and continuous perovskite crystalline films, a descending temperature gradient is applied to the assembly. This thermal regulation is achieved by transferring heat from a hotplate to the bottom FTO glass through a metal block, enabling controlled crystallization conditions. Following seven days of growth, a 120 cm^2^ crack-free CH_3_NH_3_PbBr_3_ planar crystal film was continuously deposited onto FTO glass. The initial CH_3_NH_3_PbBr_3_ crystals exhibited disordered growth orientations with dimensions below 1 mm [79]. During subsequent prolonged growth, as the precursor solution was renewed, some small crystals continued epitaxial growth along different crystal planes at anisotropic rates, ultimately forming large crystals approximately 1 cm wide and several centimeters long.

#### Size Enlargement

Nucleation and subsequent crystal growth are the two decisive steps in controlling perovskite crystal size. For instance, an excess of nuclei reduces crystal size, while insufficient precursor supply hinders the prolongation of the subsequent growth phase required for crystal enlargement. Various strategies based on the space-limiting method can regulate both nucleation and subsequent crystal growth processes, thereby enabling the expansion of perovskite crystal thin films [83]. A method for promoting precursor ion diffusion via hydrophobic interface-constrained lateral crystal growth has been developed for obtaining large-sized crystals. As shown in Fig. 6c, at low ion diffusion rates, precursors consumed during crystal growth cannot be replenished promptly, leading to interruptions in the continuous crystal growth process. Simultaneously, random nucleation occurs within supersaturated regions, resulting in the formation of numerous small crystals rather than a few large ones. Following surface modification, a marked enhancement in ion diffusion rates is clearly observable. Compared to hydrophilic surfaces (glass), ion diffusion rates on hydrophobic surfaces (ITO substrates coated with poly(bis(4-phenyl)(2,4,6-trimethylphenyl)amine) (PTAA)) are at least 100 times faster. This can be attributed to the non-wetting surface weakening solvent–substrate interactions, thereby promoting solvent diffusion. Consequently, precursor ions are not entrained during diffusion, accelerating their transport and enabling continuous perovskite crystal film growth over large areas. In summary, continuous perovskite crystal film growth is achievable when hydrophilic surfaces are replaced with hydrophobic ones. Moreover, this diffusion-enhanced spatial confinement approach is broadly applicable to both iodide- and bromide-based perovskites. The photographs display grown MAPbI_3_ and MAPbBr_3_ SCs, reaching millimeter-scale dimensions with thicknesses of 10− 20 μm [80].

#### Quality Improvement

High quality is a crucial characteristic of perovskite crystal films, playing a pivotal role in determining the photodetection performance and operational behavior of perovskite PDs. However, these films typically suffer from issues such as low crystallinity and small grain size. Enhancing the crystallinity and size of the grain structure through the space-limited method can effectively reduce the number of grain boundaries and the overall defect density, thereby suppressing carrier recombination during photodetection operations. A pressure-assisted space-constrained solvent engineering strategy (PSS) has been developed to govern the secondary growth of perovskite grains in thin films, thereby enhancing crystalline quality, with the procedure illustrated in the Fig. 6d. This process primarily comprises three steps: (1) adsorption of undesirable solvent (isopropyl alcohol, IPA) droplets onto the precursor film via steam fumigation; (2) partial dissolution of crystals as temperature and pressure increase, with solution diffusion occurring in a relatively sealed environment; (3) melting and recrystallization between adjacent grains [81]. Throughout this process, the method combines pressure assistance, spatial confinement, and solvent engineering effects to form high-quality MAPbI_3_ films. Scanning electron microscopy (SEM) results demonstrate large grain sizes and fusion between adjacent grains. Crucially, antisolvent annealing demonstrably exerts a significant influence on the quality of perovskite films, encompassing crystallinity, grain size, and grain boundaries.

#### Architecture Tailoring

The structural characteristics of perovskites are equally crucial to the performance of optoelectronic devices. For instance, perovskite materials with distinct structures, such as nanowires [84], nanodots [85], and inverted opals [86], prepared through morphological control techniques. Owing to their high surface-to-volume ratio, these structures significantly enhance light–matter interaction via multiple scattering and optical confinement effects, leading to improved light absorption compared to planar thin films. Moreover, the reduced dimensionality facilitates shorter carrier transport distances to charge-collecting interfaces, thereby suppressing bulk recombination and enhancing charge collection efficiency. Aligned microwire arrays have been developed using space-limiting methods for fabricating high-performance lateral photoconductive PDs. These arrays demonstrate remarkable advantages, where their high surface-to-volume ratio and extended Debye length not only effectively enhance light absorption capabilities, but also significantly prolong charge carrier lifetimes while reducing carrier transport times. Compared to their bulk counterparts, such microwire-based detectors exhibit significantly enhanced *R*, D*, and response speed. Furthermore, the 1D nanowire architecture offers exceptional integration flexibility, enabling facile incorporation with other semiconductor systems or direct growth on flexible substrates for advanced optoelectronic applications. Figure 6e illustrates a schematic diagram of the preparation of aligned microwires via a microchannel-confined crystallization (MCC) method [82]. The process primarily consists of three key steps. The fabrication process initiates with the creation of precisely aligned microchannels on a rigid substrate through lithographic patterning. Following this, a liquid polydimethylsiloxane (PDMS) prepolymer is uniformly deposited via spin coating onto the microchannel-bearing substrate and thermally cured, yielding an elastomeric template with faithfully replicated, well-defined microstructures. Finally, the PDMS template is contact with the target substrate, establishing hermetically sealed microchannel arrays. Precursor solution is subsequently injected from the open terminus, whereupon capillary forces drive spontaneous wicking through the entire channel network. A controlled annealing process then induces crystallization, yielding well-ordered microwire arrays. Building upon this approach, researchers have implemented several optimizations to improve microwire performance, including the development of solution immersion processes and gradient cooling crystallization methods, which significantly enhance array uniformity and single-crystal perfection [87].

Overall, the crystallization quality and microstructure of perovskite materials directly determine the ultimate performance of PDs, which is highly dependent on the material preparation process. Due to their low cost and highly flexible compositional tunability, wet methods have become the mainstream approach for fabricating polycrystalline thin-film perovskite devices in laboratories. However, the uncontrollable crystallization kinetics during solvent evaporation often leads to an abundance of grain boundary defects, and achieving uniformity over large areas remains a challenge. In contrast, although dry methods (vapor deposition) involve high time costs and more complex control of vacuum conditions and stoichiometric ratios, they enable pore-free, large-area, dense, and conformal growth while completely eliminating the issue of solvent residue. This provides a highly promising path for compatible integration with traditional silicon-based CMOS processes. To further overcome the performance bottlenecks of polycrystalline films, space-confined methods have been widely adopted for the growth of high-quality single-crystal films and low-dimensional arrays. In practical applications, specific implementation scenarios, such as flexible, wearable single-pixel devices or rigid, large-area imaging arrays, should be considered when selecting or combining these methods to develop optimal detector fabrication schemes. Although diverse material preparation techniques have laid the foundation for the fabrication of functional layers in perovskite PDs, the performance optimization of PDs still largely depends on a continuous physical process involving photon absorption, charge extraction, and long-term operation, with advancements in these areas summarized in subsequent sections.

## Photon Management Strategies for High-Performance Perovskite PDs

**Fig. 7:**
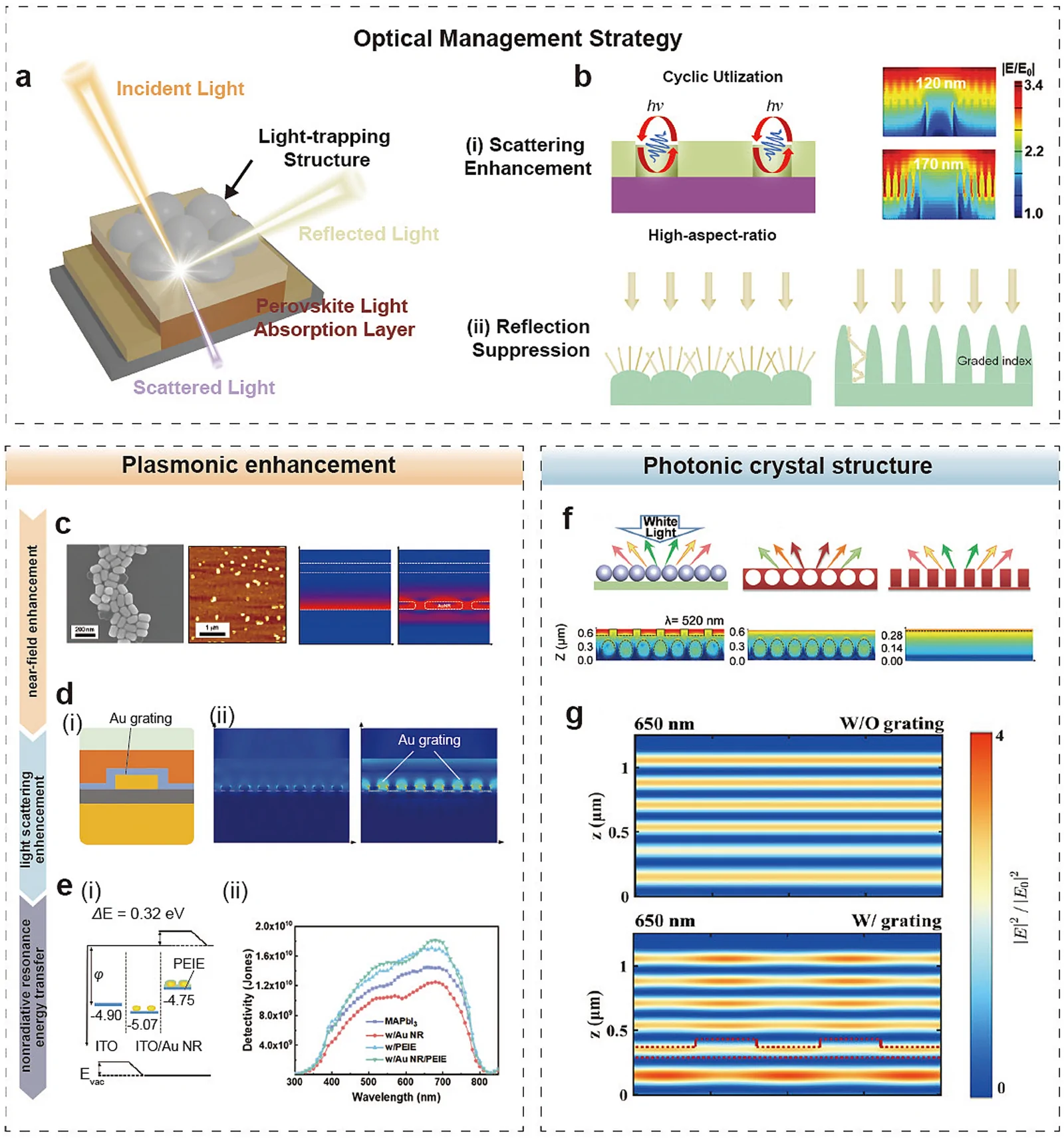
Optical management strategies for improving light utilization efficiency. **a** Schematic of light utilization in perovskite PDs. **b** Two approaches for enhancing light utilization efficiency. Reproduced with permission from Ref. [92] Copyright 2020, Wiley. Reproduced with permission from Ref. [93] Copyright 2023, Royal Society of Chemistry. **c** Near-field enhancement induced by metal nanoparticles. Reproduced with permission from Ref. [94] Copyright 2025, Elsevier. **d** Localized light scattering enhancement induced by metal nanoparticles. Reproduced with permission from Ref. [95] Copyright 2022, Wiley. **e** Nonradiative resonance energy transfer by introducing metal nanoparticles shows enhanced performance in PDs. Reproduced with permission from Ref. [94] Copyright 2025, Elsevier. **f** A composite structure of a photonic crystal and a top grating. Reproduced with permission from Ref. [96] Copyright, 2019, Wiley. **g** A bottom grating structure that reduces reflected light. Reproduced with permission from Ref. [97] Copyright 2025, Wiley

The initial stage of perovskite PD operation involves maximizing the utilization of incident light, which is limited by the finite optical path length and optical losses in the light-absorbing layer. Given the direct bandgap and high refractive index of perovskites, light management is particularly crucial for these materials. When the light beam is incident from free space onto the perovskite interface, it undergoes simultaneous reflection, refraction, and scattering as it penetrates the light-absorbing layer where it is absorbed and utilized, as shown in Fig. 7a [88]. Consequently, light scattering at the interface surface is required to be enhanced, as it can induce photon recycling near the band edge, while light reflection at the interface should be minimized to reduce light loss, as shown in Fig. 7b. Along this line, researchers have recently employed optical management strategies such as plasmonic enhancement and grating structures to modulate the light field distribution inside the devices. Plasmonic effects utilize the localized surface plasmon resonance of metallic nanostructures to generate extremely strong electromagnetic field enhancement in the near-field region, thereby improving light absorption. Grating structures, on the other hand, couple incident light into waveguide modes or produce slow-light effects through diffraction, prolonging the light–matter interaction path. These strategies significantly enhance light trapping capabilities and ultimately optimize device performance [89–91].

### Plasmonic Enhancement Strategies

Typically, constructing a low aspect ratio nanostructure using high-refractive-index dielectric materials enhances light scattering and induces enhanced surface resonance to improve light utilization. The plasmonic effect enhances light absorption by generating strongly localized electromagnetic fields near the perovskite active layer through localized surface plasmon resonance (LSPR) in metal nanoparticles (NPs) or nanostructures [98]. This enhancement mechanism operates through three primary pathways. Firstly, metal NPs excite LSPR, creating intense near-field enhancement that dramatically boost light absorption in perovskite films. Secondly, larger NPs enhance light scattering, effectively prolonging the optical path length within the active layer to improve photon harvesting. In addition, through nonradiative resonance energy transfer (NRET), plasmonic excitations can directly transfer energy to the perovskite material, thereby increasing exciton generation efficiency.

As a representative example of LSPR application, Zeng et al. incorporated Au NPs between CsPbBr_3_ NCs and a Si substrate; the introduction of Au NPs led to significant improvements in EQE and photocurrent while maintaining nearly identical baseline linearity. Meanwhile, the authors employed finite-difference time-domain (FDTD) simulations to analyze the electric field distribution around the NPs. The electric field enhancement near the Au NPs after integration with perovskite NCs was clearly demonstrated, with the electric field intensity around CsPbBr_3_ NCs being amplified by 2.5 times [99]. Kim et al. quantitatively calculated and analyzed the physical aspects of plasma enhancement to improve the photoresponse performance for perovskites PDs [94]. Firstly, they found that compared to the control device, the absorption of the active layer was enhanced when embedded with plasmonic gold nanorods (NR) or a gold NR/polyethyleneimine ethoxylated (PEIE) hybrid layer, as shown in Fig. 7c. Concurrently, the presence of PEIE prevents exciton quenching effects caused by direct contact between the plasmonic structures and the active layer. The gold NR/PEIE hybrid film shown in Fig. 7d(i) exhibits increased absorption in the photoactive layer due to the strong plasmonic effects of the gold NRs, similar to the enhanced light absorption. Moreover, within their constructed structure, PEIE formed an electric dipole layer at the perovskite/ITO-Au interface. This dipole layer allowed the work function to be altered by influencing the energy levels near the surface (from 5.07 to 4.75 eV). Under illumination, the significant electric field enhancement near Au NRs substantially increased photocarrier generation in the perovskite film and the reduced work function (W_F_) introduced by the PEIE surface modification layer facilitated electron extraction from the photoactive layer to the electrode. Consequently, the presence of the Au NRs/PEIE modified layer significantly enhanced the performance of the PD under illumination, as shown in Fig. 7e(ii).

In addition to randomly distributed Au NPs/NRs, similar enhancement effects have also made precisely aligned Au nanograting structures an effective approach to significantly boost device performance. Wang et al. introduced LSPR by embedding Au nanograting on the substrate [95]. The device architecture was constructed with an Au thin film at the bottom, a SiO_2_ dielectric spacer in the middle, and Au nanograting at the top. The aligned Au nanograting has a 150 nm width and 300-nm period. Then, a functional PD of PEDOT:PSS/(BA)_2_FA_2_PbI_7_/C8BTBT fabricated atop the plasmonic grating. As clearly evidenced in Fig. 7d(ii), the incorporation of nanograting structures induces remarkable enhancement in both optical absorption and photocurrent generation. The periodic Au nanograting simultaneously excites both LSPRs and propagating surface plasmon polaritons (SPPs). Notably, within the 700 − 1000 nm spectral range, the strong coupling between LSPRs and SPPs induces a substantial increase in absorption intensity. The pronounced near-field enhancement is found around the Au nanograting under both excitation wavelengths. In addition, deep penetration of enhanced fields into the perovskite layer facilitates extended light–matter interaction. Building on the aforementioned monolayer nanostructure, Lee et al. developed a hierarchical nanostructure characterized by nanocolumns arranged in a cross-patterned nanograting, which exhibited a significantly enhanced light trapping effect [100]. The perovskite PD based on the hierarchical pattern exhibited a responsivity of 580 mA W^−1^ and a specific detectivity of 3.2 × 10^12^ Jones, which were 420% and 990% higher than those of the perovskite PD without the nanograting structure, respectively.

### Photonic Crystal Structure

Photonic crystals are materials exhibiting periodic spatial refractive indices. This periodic refractive index endows photonic crystals with photonic bandgaps, where light of specific wavelengths cannot propagate through the structure, enabling them promising candidates for reflective polarizers and optical waveplates [93]. Consequently, the incorporation of photonic crystal structures in perovskite PDs enables the suppression of spontaneous emission at targeted wavelengths while significantly improving light trapping efficiency [101].

Among various photonic crystal configurations, polystyrene (PS) microspheres have emerged as a dominant structural motif [102]. For example, Oh et al. fabricated a PS microbead array atop the perovskite layer using a rubbing and transfer process [103]. The PS bead monolayer was located between two PDMS layers, followed by adhesion of the PS beads to the polymethyl methacrylate (PMMA) layer via compression from the upper PDMS layer. The generated interfacial light trapping enhanced absorption and response of PD. Remarkably, the strategic coupling of photonic crystal designs with nanoscale grating elements creates multifunctional platforms that simultaneously address light management challenges while enabling novel operational modalities in perovskite PDs. Inspired by the hierarchical structure of the butterfly, Zhan et al. proposed a 1D nanograting-bonded porous 2D photonic crystal perovskite PD (G-PC-PD) with high performance and polarized light detection capability [96]. The authors developed a simple, cost-effective, and reproducible fabrication method combining nanosphere lithography and commercial DVD nanoimprinting to construct this hierarchical light trapping perovskite PD. As shown in Fig. 7f, the field intensity distributions in perovskite films with various optical structure calculated by 3D FDTD method demonstrated that the 2D PC layer provided the feedback effect owing to its stopband. Meanwhile, the nanograting structure could reduce the light reflection and enhance the light trapping in the active layer through grating diffraction due to its height-to-width ratio (Fig. 7g(i)) [97]. Besides, a waveguide mode was formed in the middle perovskite layer between the grating and PC layers for both of them are porous and their effective refractive indexes are lower than that of the close-grained perovskite layer. The synergistic effects of diffraction and reflection coupling induced by the grating and PC structures significantly enhanced light trapping in the G-PC-PD. Further, the porous scaffold and nanoimprinting process improved the crystallinity of the nanograting and photonic crystal perovskite film, enabling a remarkable white-light *R* of 12.67 A W^−1^ and a D* of 3.22 × 10^13^ Jones, as shown in Fig. 7g(ii), which is 6 − 7 times higher than those of PDs with dense perovskite films.

In this section, we have summarized photon management strategies, exemplified by photonic crystals and plasmonic-enhanced light absorption, to enhance the performance of perovskite PDs. In certain applications where the thickness of the absorption layer is limited, such as in high-speed optical communications or flexible electronics, photon management strategies are particularly crucial due to the ultra-thin nature of the absorption layer. It is essential to rely on micro- and nano-optical structures to forcibly enhance localized light trapping, thereby resolving the conflict between thin film and weak absorption. It must also be acknowledged that the microstructures inevitably increase the roughness and defect density at the heterojunction interface, necessitating the integration of charge carrier collection strategies to mitigate these detrimental effects.

## Charge Extraction and Interface Design for High-Performance Perovskite PDs

**Fig. 8:**
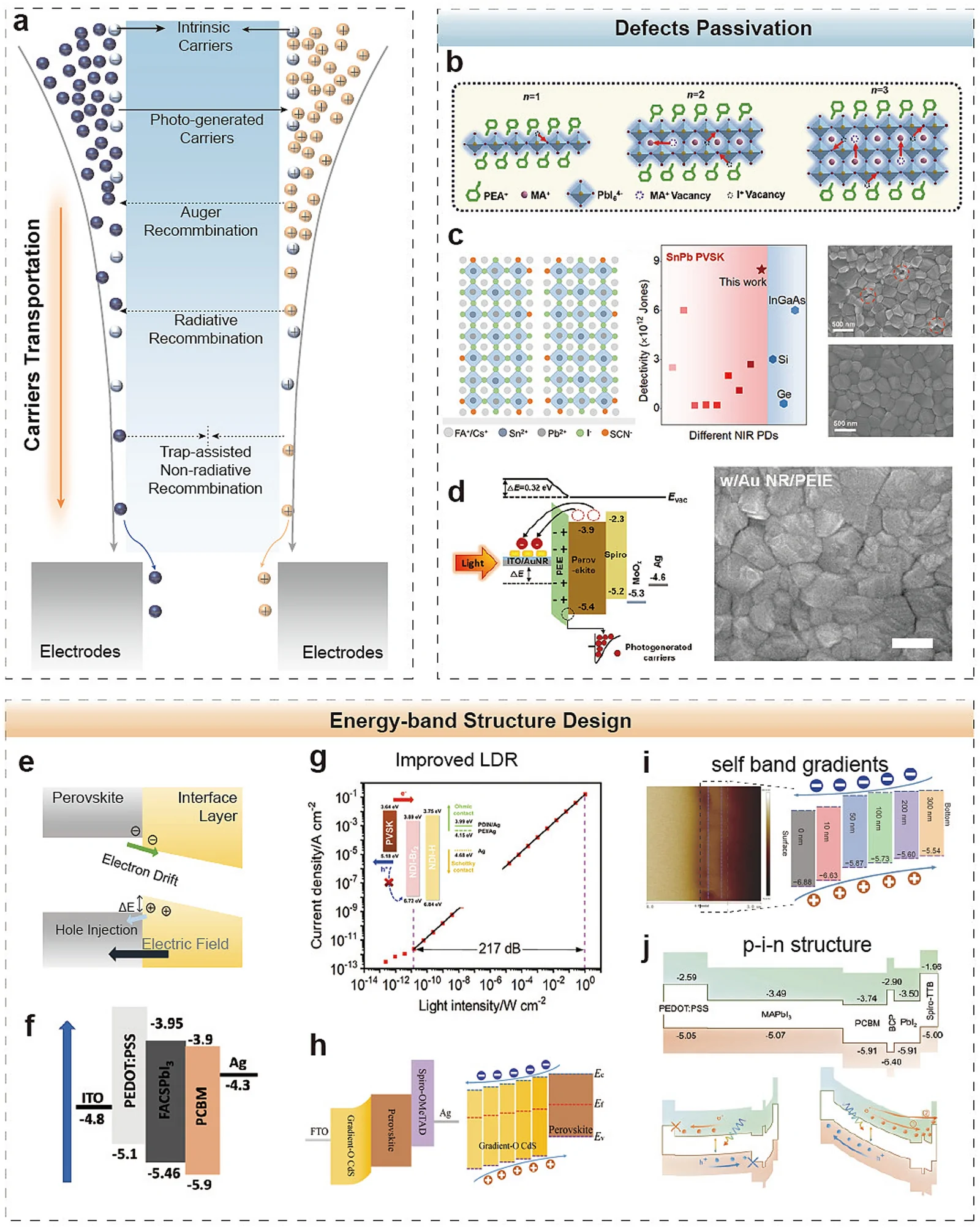
Optimization strategies for charge collection. **a** Carrier dynamic processes in perovskite PDs. **b** Ion migration pathways and the process of forming vacancy defects in two-dimensional perovskites with different n values. Reproduced with permission from Ref. [109] Copyright 2024, Wiley. **c** Crystallization control of FA_0.85_Cs_0.15_Sn_0.5_Pb_0.5_I_3_ perovskite film. Reproduced with permission from Ref. [110] Copyright 2023, American Chemical Society. **d** Schematic diagram illustrating utilization of the PEIE passivation layer to optimize crystal quality and suppress dark current. Reproduced with permission from Ref. [94] Copyright 2025, Elsevier. **e** Schematic band diagrams for band alignment and contact engineering. **f** Effect of different electronic transport layers on noise current in ITO/PEDOT:PSS/FA_0.95_Cs_0.05_PbI_3_/ETL/BCP/Ag devices. Reproduced with permission from Ref. [111] Copyright 2023, American Institute of Physics. **g** PD performances based on different electronic transport layers (NDI-Br_2_ and PC_61_BM). Reproduced with permission from Ref. [112] Copyright 2025, Wiley. **h** Schematic representation of perovskite/CdS device structure and the gradient energy levels. Reproduced with permission from Ref. [113] Copyright 2019, Wiley. **i** Gradient energy levels and carrier transport at the CsPb_0.7_Sn_0.3_I_3_ (140 nm)/PCBM interface. Reproduced with permission from Ref. [114] Copyright 2023, Springer Nature. **j** Schematic energy diagrams and performance of the PIN-BD PDs. Reproduced with permission from Ref. [115] Copyright 2022, Wiley

While effective optical management significantly enhances the concentration of photogenerated carriers, if these carriers undergo severe non-radiative recombination as they cross the heterojunction interface, the benefits gained from earlier light harvesting will be completely negated. Therefore, the critical step following optical management is charge collection optimization. The carrier density (current density) detectable in the PD after illumination originates from two components, intrinsic carriers and light-generated carriers [104]. Both collectively determine the performance of the PD: The former significantly influences the dark current and noise of the device, while the latter dictates key parameters such as on/off ratio, responsivity, and detection efficiency. After light-induced carrier generation, free carriers can readily be captured by non-radiative recombination centers, primarily energy level traps, especially at high charge carrier densities [105]. Furthermore, free electrons located in the conduction band may undergo radiative recombination with free holes. These two recombination processes are conventionally recognized as unimolecular and bimolecular recombination, respectively. Additionally, under high-intensity illumination, the accumulation of carriers readily triggers Auger recombination, resulting in energy conversion to thermal energy, as shown in Fig. 8a [106]. Hence, charge collection optimization, collecting the most photogenerated charges while simultaneously mitigating intrinsic charge effects, is critical for fabricating high-performance perovskite PDs. Efficient charge extraction and transport minimize recombination losses, leading to higher responsivity, faster response times, and improved detectivity. Recombination losses mainly originate from non-radiative recombination of carriers in the bulk or at interfaces, such as trap-assisted Shockley–Read–Hall recombination processes. From the perspectives of material optimization and structural design, recent research on charge collection optimization has focused on two physically well-defined approaches: defect suppression and interface engineering [107, 108]. The core of defect suppression lies in the chemical or physical passivation of deep-level or shallow-level traps in the film. This not only improves the crystalline quality of the film and reduces leakage current paths to suppress the intrinsic dark current, but also prolongs the effective carrier lifetime and enhances carrier mobility, allowing more photogenerated minority carriers to diffuse into the depletion region and be collected. Meanwhile, interface engineering achieves efficient charge extraction primarily by tuning the band alignment between the functional layers of the device. Specifically, by rationally selecting charge transport layers or introducing interfacial modification layers, a stepped or type-II band structure can be formed at the interface, reducing the hole or electron injection barrier and establishing a favorable built-in electric field. This maximally drives the directional transport of photogenerated charges toward the external electrodes and suppresses charge accumulation and recombination losses at the interfaces. The synergistic effect of the above two mechanisms ultimately ensures that the device achieves near-ideal optoelectronic conversion efficiency.

### Defects Passivation

The tiny grains, abundant defects in the crystal lattice, and grain boundaries in the perovskite layer provide high surface traps state, which offers potential pathways for ion migration and water ingress, leading to increased dark current and degraded detection performance [115]. Furthermore, this will increase the noise of the PD, one of the most critical limiting factors for perovskite PDs performance, particularly in low-light-intensity or high-sensitivity applications. Shot noise is the primary and controllable noise source, originating from random fluctuations of charged particles and commonly presented in PDs. According to the mean square deviation of fluctuation, shot noise in the dark (*I*_nd_) can be calculated as following equation:

14 $$I_{nd} = \sqrt {{2eI_{d}} \Delta f}$$

And *NEP*, also known as minimum detectable power, is defined as the incident power when the signal-to-noise ratio is 1, which is an important performance parameter for measuring the ability of PDs to detect weak signals and noise suppression.

From the above two equations, it can be concluded that dark current is an important parameter determining device noise and detection capability; therefore, reducing dark current is the most direct way to suppress noise and improve SNR. Therefore, the effect of defect passivation will primarily manifest in suppressing the intrinsic carrier concentration and dark current.

3D perovskite materials exhibit a higher defects self-doped carrier concentration compared to their extrinsic carrier concentration, which significantly increases the dark-state noise current and consequently reduces the SNR [116]. Compared to 3D perovskites, 2D perovskites theoretically have a lower dielectric constant, enabling the dark current suppression. The exceptional performance of 2D perovskite materials can be attributed to their unique molecular structure, which forms a quantum well structure through the alternating arrangement of inorganic and organic layers [117]. This arrangement not only provides efficient carrier conduction pathways within the quantum wells but also forms effective electronic and ionic transition barriers between adjacent quantum wells. Cui’s group synthesized a series of PEA_2_MA_n-1_PbnI_3n+1_ (*n* = 1, 2, 3) two-dimensional perovskites and systematically investigated the influence of n values on the weak light detection capability of photodiodes constructed using these materials [109]. Notably, perovskite PDs based on the (PEA)_2_ PbI_4_ structure with *n* = 1 exhibit extremely low noise current levels (10^−15^ A Hz^−1/2^ in the dark state, significantly lower than those with higher n values. The (PEA)_2_PbI_4_-based PDs achieved an ultra-low detection limit of 14 pW cm^−2^ under 532-nm illumination at 1 V, demonstrating exceptional detection performance of 3.25 × 10^15^ Jones. The improved performance can be attributed to the total cation PEA^+^ ratio increasing with n value, which effectively passivates defects while increasing the activation energy and exciton binding energy of ion migration, thus reducing noise currents caused by the electrons/holes, ions, and excitons dynamic behavior, as shown in Fig. 8b.

Numerous fine grains and cracks at grain boundaries within pure perovskite films provide ample opportunities for defect formation, leading to carrier recombination and detrimental current leakage. Primarily, controlling crystal quality can effectively suppress grain boundaries and passivate surface defects. Lee et al. reported a perovskite granular wires PD with ultra-high specific detectivity (3.17 × 10^15^ Jones) synthesized via a simple and effective template-free self-assembly process [118]. Its exceptional photodetection performance stems from the unique morphology-derived band-edge modulation exhibited by the nanowires, where the energy barrier effectively suppresses interface defects, resulting in an extremely low dark current at 1 V bias. The incorporation of certain additives can effectively improve perovskite morphology and passivate device defects. As illustrated in Fig. 8c, Choy’s group reported that the addition of Sn(SCN)_2_ to Sn–Pb perovskite resulted in a smooth perovskite film free of pinholes and cracks [110]. When the additive is introduced into precursor solutions, Sn(SCN)_2_ would contribute to pre-existing nuclei composed of soft complexes of organic components coordinated with inorganic components due to the strong coordination between SCN^−^ and metal cations. The mechanism relied on the precise modulation of crystallization kinetics mediated by Sn(SCN)_2_. It is observed that the grain growth at the top is largely suppressed when the concentration of the Sn(SCN)_2_ additive is increased from 0 to the optimal 4%, in which grains at the top show greatly reduced sizes and even largely coalesce with grains at the bottom. This engineered perovskite structure, with unique additive distribution, markedly improved film morphology, reduced grain boundaries and intergranular defects, and enhanced oxidation resistance. Consequently, the devices obtained superior photodetection performance, including reduced dark current density at 0.1 V, exceptional NIR detection capability, and a broad dynamic range.

The collaborative application of passivation defects with other strategies for enhancing the optoelectronic performance of devices is also a matter requiring attention. As mentioned in the optical management strategy, the interfacial effects of nanoparticles offer advantages in regulating light propagation/absorption within the perovskite layer, yet simultaneously exacerbate dark current, attributable to the poor morphology and low crystallinity of perovskite film [119]. In this regard, on the basis of achieving photocurrent enhancement using gold nanorods (Au NRs) via the LSPR effect, Kwon et al. introduced a polyethyleneimine ethoxylated (PEIE) passivation layer between the ITO-Au NR layer and the ITO-Au NR layer/CH_3_NH_3_PbI_3_ interface to suppress device defects and dark current, as shown in Fig. 8d [94]. This method effectively mitigated the dark current degradation caused by the introduction of Au NRs. Following the introduction of Au-NR, the dark current of the plasmonic PDs increased tenfold compared to the original device at 1 V bias. However, with the assistance of the PEIE interface layer, the dark current was significantly reduced to a level close to that of the original perovskite device, simultaneously achieving dark current suppression and enhanced light harvesting.

### Interface Engineering

Charge collection optimization is critical for fabricating high-performance perovskite PDs. Efficient charge extraction and transport minimize recombination losses, leading to higher responsivity, faster response times, and improved detectivity. To optimize charge collection efficiency, energy level alignment and graded heterojunction structure design have been extensively investigated [120, 121].

Energy level matching between the perovskite layer and adjacent charge transport layers becomes critical for ensuring effective dissociation of electron–hole pairs. A rational energy level alignment scheme facilitates carrier generation, separation, and transport, as shown in Fig. 8e. The underlying physical mechanism is that photogenerated excitons need to overcome the exciton binding energy to dissociate into free electrons and holes [122, 123]. An appropriate energy level offset between the absorber layer and adjacent functional layers provides a thermodynamic driving force for exciton dissociation and ensures efficient separation of electrons and holes along their respective downhill energy pathways. Meanwhile, a rational energy level alignment also avoids the formation of energy barriers at the interfaces, thereby reducing carrier accumulation and interfacial recombination losses. Here, an ETL or HTL is commonly introduced to achieve effective separation and collection of electron–hole pairs. The ETL should possess a low-lying lowest unoccupied molecular orbital (LUMO) or conduction band energy level that matches the conduction band of the absorber layer, allowing rapid extraction of electrons while blocking holes [124–126]. Correspondingly, the HTL should match the valence band energy level of the absorber layer to extract holes and block electrons. Together, the two layers establish a built-in electric field and an energy gradient, ensuring that photogenerated carriers are directionally transported to their respective electrodes, thereby maximizing charge collection efficiency [127].

Simultaneously, mobile ions within the perovskite layer, particularly iodide ions, gradually diffuse into adjacent interface layers and accumulate near electrodes, leading to electrode corrosion and phase separation/dealloying of the perovskite layer. A direct approach involves inserting an ion barrier layer (IBL) onto the perovskite surface to physically inhibit ion migration from the perovskite layer to adjacent layers [128]. The introduction of the IBL not only suppresses ion migration to adjacent layers and mitigates electrode corrosion on a macroscopic scale, but also inhibits the formation of interface defects and decouples interface ion–electron coupling. By creating a chemically and physically robust barrier with high resistance, the IBL layer successfully prevents the formation of localized space charge fields caused by interface defects, thereby facilitating the efficient separation and collection of charge carriers.

Recent studies have demonstrated that carefully designing the ETL and IBL can achieve favorable energy level matching between different layers, enabling directed charge carrier transport within the perovskite PDs. Fullerene derivatives such as [6, 6]-phenyl-C61-butyric acid methyl ester (PC_61_BM) are the most commonly used ETL materials in perovskite PDs. Bhardwaj et al. systematically investigated the effects of thickness and type of fullerene-based electron transport layers on interfacial charge transfer and blocking [111]. They found that besides blocking interfacial charge recombination, the performance improvement of the perovskite PD with the EBL PC_70_BM compared to PC_60_BM is attributed to its smoother PC_70_BM-perovskite interface, which reduces leakage/noise current, as shown in Fig. 8f. Taking the appropriate PC_70_BM layer can effectively suppress dark current and noise current, yielding improved performance, including a *R* of 0.46 A W^−1^, a D* exceeding 10^12^ Jones, and a linear LDR > 90 dB in the NIR band (approximately 780 nm) under 0 V bias. To overcome the instability of fullerenes caused by thermal stress, Chang et al. selected superior EBL/IBL layers, such as derivatives of naphthalene diimide (NDI), to more precisely design the interlayer energy level structure, allowing the desired directional flow of charge carriers within the devices [112]. As shown in Fig. 8g, the energy offset between the conduction band of the perovskite layer and the LUMO level of the adjacent NDI-Br₂ was 0.25 eV. This indicated that NDI-Br_2_ displayed strong electron-accepting capability due to its electron-withdrawing dibromo substituent, suggesting the ability to form a heterojunction interface for efficient electron–hole pair dissociation. Furthermore, the deep highest occupied molecular orbital (HOMO) energy level of the NDI-Br₂ layer (6.72 eV) could block hole carriers from perovskite toward the cathode, which was advantageous for reducing unnecessary charge recombination within the device. Based on these results, the excellent energy matching between layers was demonstrated, allowing the desired directional flow of charge carriers within the devices. Consequently, the fabricated PD exhibited high performance with high *R* of 0.53 A W^−1^, high D* of 3.58 × 10^14^ Jones, and a wide LDR of 217 dB in a self-power mode.

Besides adding transport layers, delicate tuning of the transport layer bandgap also plays important roles, which can equally enhance the extraction and transfer of photogenerated carriers. Cao et al. presented a self-powered PD based on the integrated gradient O-doped CdS nanorod array and perovskite (Fig. 8h) [113]. By precisely tuning the energy bands gradient distribution of CdS, they achieve optimized continuous band bending that concurrently enhances the separation and transport efficiency of photogenerated carriers while effectively suppressing interfacial recombination processes. The gradient energy levels create a continuous built-in band bending across the range from the surface to the core of nanorods, which facilitates the transfer of photogenerated electrons from the surface to the core and the backflow of photogenerated holes into the perovskite. This continuous band bending also prevents electrons from flowing back from of CdS_10_, thereby preventing recombination with holes. Hence, under the self-powered voltage mode, the rise and decay time of the optimized device are 0.54/2.21 ms, substantially shorter than the control device. The bandgap gradient can also be continuously tuned by modulating the dimensionality of the light absorption layer, thereby enhancing carrier separation efficiency.

From a materials design perspective, engineering the bandgap gradient distribution within a single light absorption layer facilitates the separation and transport of photogenerated carriers. For example, Liu and co-workers presented a gradient inorganic CsPb_0.7_Sn_0.3_I_3_-based self-powered PD [114]. Figure 8i demonstrates that controlled annealing temperature modulation enables optimal Sn/Pb compositional gradient engineering in CsPb_0.7_Sn_0.3_I_3_ films. This process primarily relies on the fact that the crystallization rate of Sn-based perovskites is faster than the formation rate of Pb-based perovskites. Additionally, as the annealing temperature increases, the crystallization rate of the bottom Sn-based perovskite accelerates, while that of the Pb-based perovskite remains relatively unchanged. Consequently, adjusting the annealing temperature allows for modulation of the energy band distribution. The resulting graded perovskite device achieves a high *R*, large D*, and fast response time at 0.005 V and under 700 nm light. These improvements originate from band bending induced by the precisely tuned Sn–Pb gradient, which creates a built-in electric field that simultaneously enhances carrier separation efficiency and accelerates charge transport.

Combining the modulation methods described above, Zhan et al. developed a more complex structure design to realize diverse functions using a perovskite photodiode-barrier diode (PIN-BD) cross-array [115]. As shown in Fig. 8j, the device structure in the array was configured as ITO/PEDOT:PSS/MAPbI_3_/PC_61_BM/BCP/PbI_2_/Spiro-TTB/Ag, in which the PIN perovskite photodiode was stacked by a reverse BD and the PCBM/BCP serves as the co-cathode. Under illumination, incident photons generated electron–hole pairs due to the intrinsic absorption characteristics of the perovskite active layer. These electron–hole pairs were efficiently separated by the intrinsic barrier and asymmetric electron/hole barrier. The presence of BD introduced a barrier formed by lead iodide and Spiro-TTB, preventing electron transfer to the Ag electrode. On the one hand, this mitigated damage to the perovskite film from Ag⁺ ions. On the other hand, electrons drifting toward PEDOT:PSS under forward bias were blocked by the PEDOT:PSS/MAPbI_3_ interface barrier, while holes drifting toward PCBM were effectively blocked by the MAPbI_3_/PCBM interface barrier, preventing harmful carrier collection. Yet it does not hinder the efficient photoresponse of the PIN-BD devices under negative bias. When the PIN-BD PD was operated under high negative bias, the built-in potential increased as electrons gained sufficient energy to overcome the barrier and be collected by the electrode. Due to the improved charge extraction efficiency, the EQE increased with the external bias voltage. At − 1.0 V, the EQE reached its saturation limit, exceeding 45% at a wavelength of 710 nm.

This section systematically explores synergistic strategies for achieving precise control of interface charge carriers through defect passivation and energy band design. In fact, these two strategies are conceived from fundamentally different starting points. Interface passivation is used to control non-radiative recombination in order to reduce carrier generation and dark current, whereas interface energy band design aims to create a barrier-driven, highly efficient photocarrier collection. For different device structures, their applicability is not entirely identical. For example, in photodiode devices used for high-frequency imaging, defects and carrier collection must be strictly controlled. However, for some emerging multifunctional photoconductive/synaptic devices, interface engineering no longer focuses on defect elimination but instead utilizes the carrier trapping effect by precisely customizing the trap density through energy level design.

## Stability Improvement and Encapsulation for High-Performance Perovskite PDs

Once excellent optoelectronic performance has been realized through the synergy of optical management and interface engineering, the final hurdle for practical application of perovskite PDs is their operational lifespan in complex environments. In fact, many interface passivation layers employed for charge collection optimization also serve to block the ingress of water and oxygen and suppress ion migration. In fact, for perovskite PDs, stability is the primary factor preventing them from replacing commercial PDs. Therefore, further systematic exploration of strategies for improved stability is essential to protect the fragile internal photoconversion mechanisms from degradation caused by environmental factors and operational stresses.

**Fig. 9:**
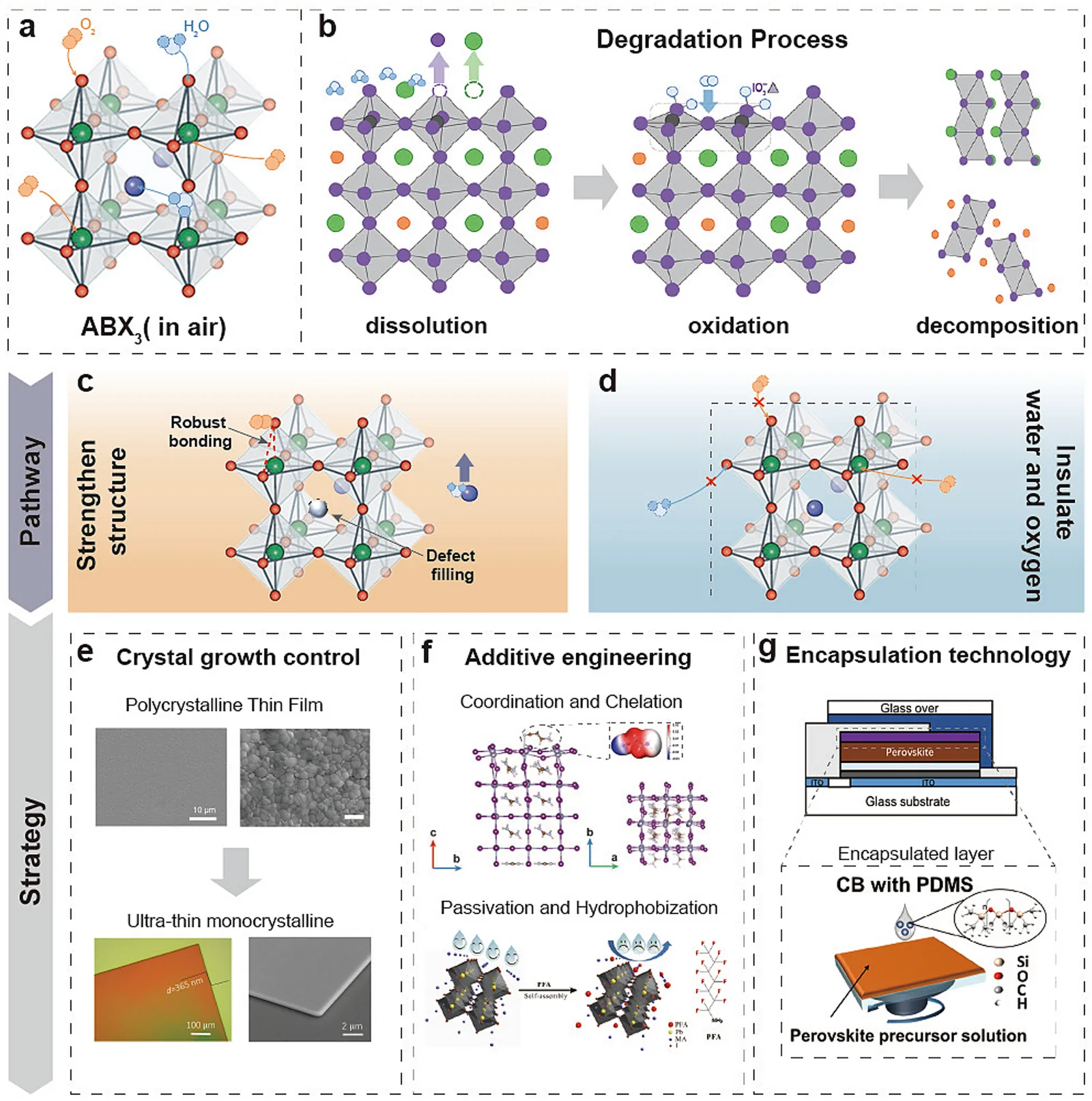
Strategies summary for enhancing the perovskite PDs stability. **a** Perovskite crystal structure. **b** Schematic of perovskite degradation process. Reproduced with permission from Ref. [130] Copyright 2023, American Chemical Society. Two pathways for improving perovskite stability: **c** enhancing the inherent structural stability and **d** preventing water and oxygen corrosion. Specific strategies for enhancing stability involving **e** crystal growth control, Reproduced with permission from Ref. [134] Copyright 2022, Wiley. Reproduced with permission from Ref [135] Copyright 2018, Wiley; **f** Additive engineering, Reproduced with permission from Ref. [136] Copyright 2024, Wiley. Reproduced with permission from Ref. [137] Copyright 2018, American Chemical Society; **g** Encapsulation technology. Reproduced with permission from Ref. [138] Copyright 2025, Wiley

The instability of perovskite materials stems from their inherent structural fragility, which causes phase transitions and phase separation under external conditions such as humidity, light exposure, and electrical fields, thereby limiting their practical application in the photodetection field [129]. A typical ABX_3_ perovskite structure is shown in Fig. 9a. Moisture (H_2_O) and oxygen (O_2_) in the air inevitably induce a phase transition from the perovskite structure to a non-perovskite structure [130]. We use a typical FAPbI_3_ molecule to detail the perovskite degradation process, as illustrated in Fig. 9b. Firstly, H_2_O molecules dissolve FAI on the perovskite surface, causing volatilization of iodide and FA⁺ cations. Subsequently, O_2_ may interact with the exposed lead iodide-rich surface, oxidizing iodides and forming the thermodynamically favored iodate species IO_3_⁻. Lead iodate Pb(IO_3_)_2_ can form and separate at the surface, leaving PbI_2_ vacancies and allowing further penetration of H_2_O molecules into the crystal structure. This degradation occurs under both dark and light conditions, with light accelerating the formation of the secondary phase. For some easily oxidized perovskite structures, such as Sn-based or Cu-based perovskite phases, the destructive effects of O_2_ molecular oxidation are even more pronounced [52]. Hence, it can be concluded that the susceptibility of perovskite to decomposition and phase transition stems from the combined effects of its inherently unstable structure and water–oxygen erosion (Fig. 9c, d). This phase separation will significantly affect the performance of perovskite PDs, including the device signal-to-noise ratio, dynamic response, and long-term operational lifetime. For example, the formation of the Pb(IO_3_)_2_ phase leads to a sharp increase in bulk series resistance, significantly reducing the collection efficiency of photogenerated carriers, causing the EQE and responsivity to decline precipitously [130]. In Sn-based perovskites, the oxidation-generated Sn^4+^ ions create a large number of Sn vacancies in the light-absorbing layer, significantly increasing the dark carrier concentration and causing dark current to rise exponentially, thereby overwhelming weak light signals [131]. Furthermore, under sustained operating heat or high-temperature conditions, the enhanced migration of halide ions in the perovskite leads to significant ions accumulation at the charge transport layer interface. These ions hinder the directed migration of photogenerated carriers under external voltage, resulting in irreversible baseline current drift and responsivity degradation.

Correspondingly, the path to enhancing the stability of perovskite PDs lies in improving structural stability and isolating external water and oxygen. Building on this principle, various specific and actionable strategies, including crystal growth control, additive modification, and interface encapsulation [132, 133], have been proposed aimed at enhancing the inherent stability of materials and improving interface contacts, as shown in Fig. 9e–g.

### Crystal Growth Control

**Fig. 10:**
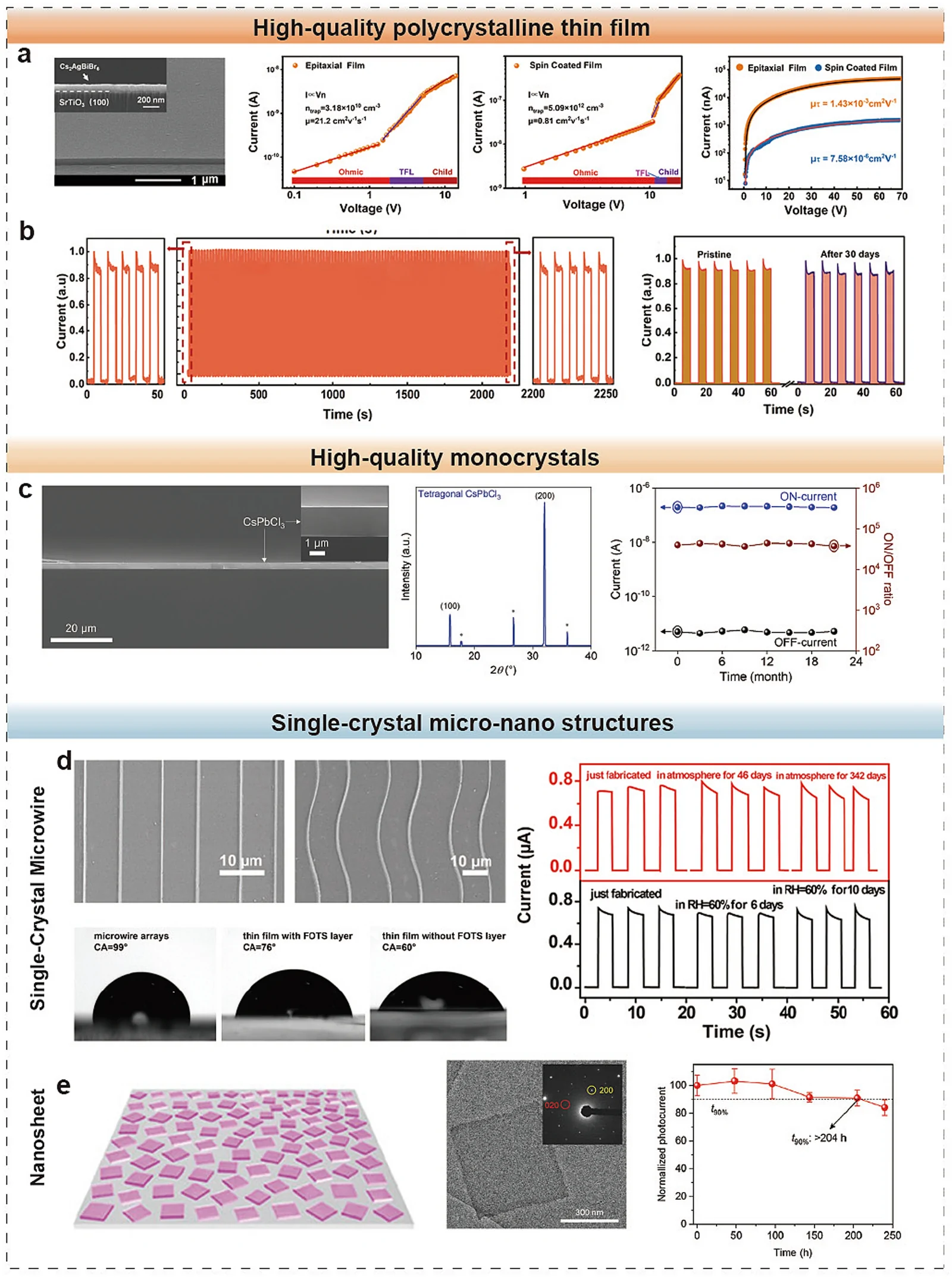
Enhanced stability in crystal growth control. **a** Crystallization control of polycrystalline thin films. **b** Thermal stability of epitaxial polycrystalline thin-film perovskite PDs. Reproduced with permission from Ref. [139] Copyright 2025, Royal Society of Chemistry. **c** Ultra-thin large-area perovskite SCs for perovskite PDs. Reproduced with permission from Ref. [143] Copyright 2023, Wiley. **d** The effectiveness of perovskite single-crystal microwires in enhancing stability. Reproduced with permission from Ref. [144] Copyright 2020, Wiley. **e** Monocrystalline perovskite nanosheet-based PDs with outstanding stability. Reproduced with permission from Ref. [64] Copyright 2025, Wiley

Polycrystalline perovskite films are widely employed in perovskite PDs manufacturing due to their simple preparation methods and suitability for large-area integration. However, the heterogeneous distribution of grain boundaries and grain sizes in polycrystalline perovskite films leads to high defect densities and poor stability. To improve this random and uncontrollable growth, Shan et al. epitaxially grew high-quality lead-free perovskite Cs_2_AgBiBr_6_ films on (100)-oriented STO substrates using pulsed laser deposition (PLD) technology [139]. Compared to spin coating techniques, the epitaxial method effectively suppressed defects concentration, including point defects, grain boundaries, and interface defects. The XRD reciprocal space mapping (RSM), 2θ-ω and phi-scans confirmed the good epitaxial relationship between the film and the substrate. Quantitative analysis based on space charge-limited current revealed that epitaxial Cs_2_AgBiBr_6_ films exhibited a two-order-of-magnitude reduction in defect density and a three-order-of-magnitude increase in carrier mobility compared to spin-coated films as shown in Fig. 10a. PDs based on such epitaxial Cs_2_AgBiBr_6_ films exhibit high responsivity of 12.1 A W^−1^, fast response times of 0.11/0.16 ms, and high specific detectivity of 4.63 × 10^12^ Jones. More importantly, the epitaxial perovskite PDs demonstrated exceptional operational stability during aging tests: Its photodetection capability remained nearly unchanged after continuous switching for 40 min and continuous illumination over 30 min, as shown in Fig. 10b. Furthermore, after storage for 30 days in air, the dark-to-bright current ratio remained comparable to that of the initial device, proving its outstanding environmental stability.

Single-crystal perovskite materials exhibit fewer grain boundaries and lower trap density, typically demonstrating superior carrier mobility and stability compared to polycrystalline materials [140]. However, their practical application is constrained by high thickness and small dimensions [141]. The thickness and dimensions of bulk SCs grown by solution methods are often difficult to control, particularly in achieving quasi-two-dimensional morphologies compatible with established integration techniques [142]. As an alternative, Duan’s group successfully grew wafer-scale, single-phase CsPbCl_3_ single-crystal thin-film phototransistors with excellent crystallinity and outstanding optoelectronic properties on mica substrates using vapor phase epitaxy (VPE) [143]. Simultaneously, they fabricated lateral photoconductive PDs on CsPbCl_3_ single-crystal thin-film transistors by transferring gold electrodes, without any charge separation layer or surface passivation layer. The SEM images revealed the CsPbCl_3_ thickness to be approximately 1.86 µm, exhibiting no grain boundaries or pinholes over large areas, indicating its high quality and favoring its flexible applications due to the reduced thickness, as shown in Fig. 10c. The CsPbCl_3_ SCs film-based PDs exhibited outstanding performance under 375-nm laser illumination, including an ultra-low dark current of 7.18 pA, an exceptionally high on/off ratio of approximately 5.22 × 10^5^ at an irradiance of 27.9 W cm^−2^, competitive *R*, EQE, and D* of 32.8 A W^−1^, 10,867% and 4.22 × 10^12^ Jones at 4.4 µW cm^−2^ light illumination, respectively. More importantly, this UV PD demonstrated exceptional stability in ambient air at room temperature over 21 months, with good high- and low-temperature tolerance and significant recovery capability after the first continuous rapid heat–cool–heat cycling test.

Compared to thin-film morphology, certain 2D/1D single-crystal structures fabricated using specialized methods such as space-confined techniques can combine the advantages of easily large-area integrated in polycrystalline films with the low defect density and high stability of single-crystal materials. Li et al. developed a template-assisted method for fabricating MAPbBr_3_ micro-/nanowire structures, achieving encapsulation through in situ coating with a protective hydrophobic molecular layer [144]. The protective layer, combined with high crystalline quality and highly ordered microstructure, significantly enhanced the stability of the MAPbBr_3_ single-crystal microwire array (Fig. 10d). Water contact angle measurements revealed that the highly ordered microstructure of MAPbBr_3_ confined microwires array (SCMWA) contributed to improved stability in humid environments. Similarly, after several days of exposure in a high-humidity environment, the response to pulsed light remained virtually unchanged, indicating that the MAPbBr_3_ SCMWA-based PD exhibits outstanding stability. Lee et al. demonstrated the application of two-dimensional PVSK nanocrystals in enhancing stability and achieving high-yield production of wafer-scale perovskite PDs arrays, as shown in Fig. 10e [64]. Thermal stability testing on these 2D single-crystal nanoribbons at 90 °C demonstrated their exceptional stability, with the perovskite PDs retaining 90% of the initial photocurrent over 200 h. The operational thermal stability at this temperature level is comparable to that of other lateral single-crystal perovskite PDs.

### Additive Engineering

**Fig. 11:**
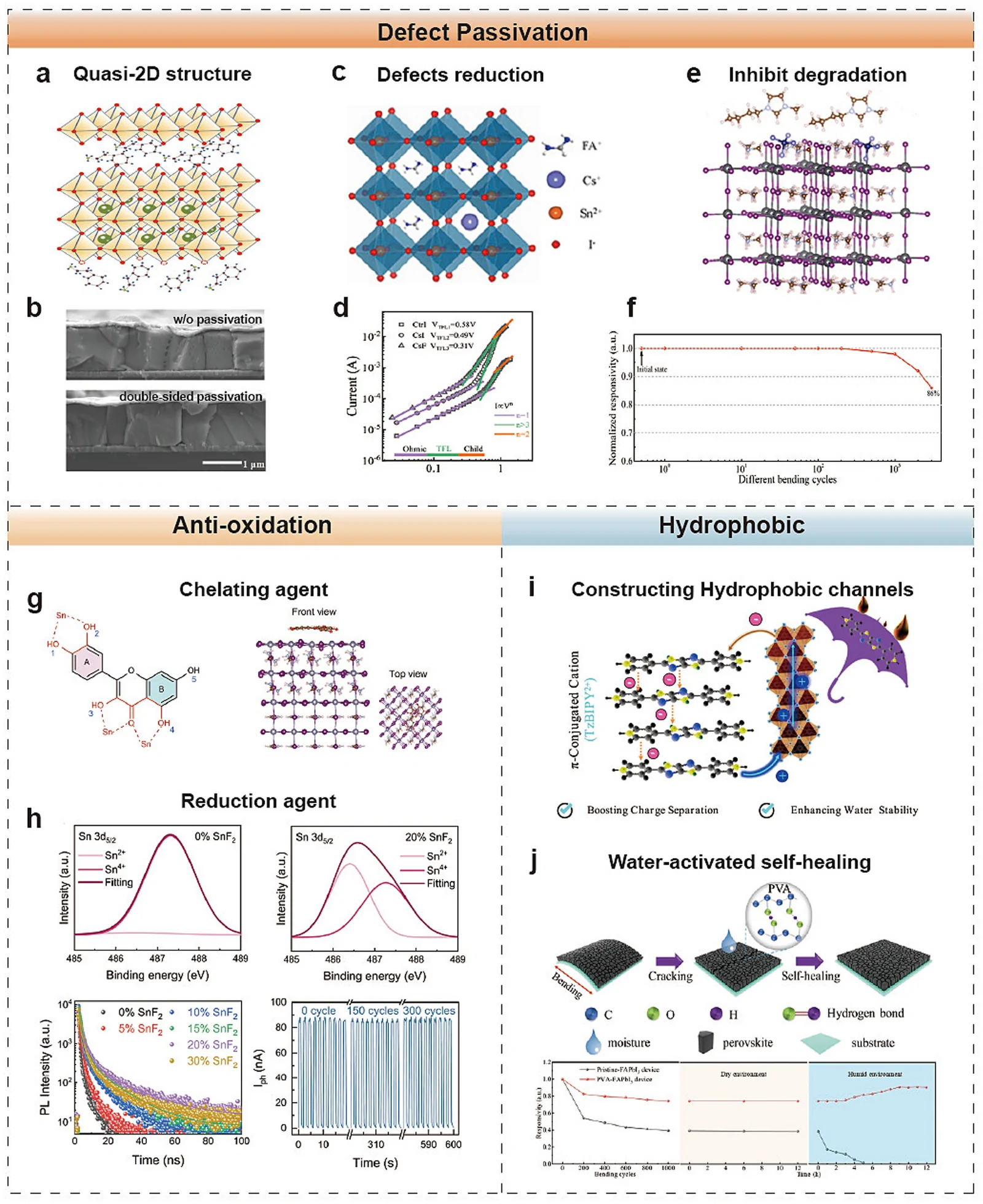
Improved perovskite PDs stability by additive engineering. **a** Schematic of PEA^+^ passivating iodide vacancy and 2D perovskite. **b** Cross-sectional SEM images of perovskite films without and with passivated HTL side. Reproduced with permission from Ref. [146] Copyright 2020, Wiley. **c** Crystal structure of the CsF-doped FASnI_3_ perovskite. **d** Reduction in defect density for improved FASnI_3_. Reproduced with permission from Ref. [147] Copyright 2024, American Chemical Society. **e** Schematic diagram of MAPbI_3_ nanowire with BMIMBF_4_. **f** Ultra-long stability achieved through additive engineering. Reproduced with permission from Ref. [148] Copyright 2022, Wiley. **g** Schematic diagram illustrating the inhibition of Sn oxidation through the chelation of Sn atoms by the quercetin molecule. Reproduced with permission from Ref. [149] Copyright 2025, Springer Nature. **h** Suppressing tin oxidation by adding SnF₂ as a reducing agent. Reproduced with permission from Ref. [150] Copyright 2023, Wiley. **i** Construct hydrophobic channels to prevent perovskite from being eroded by water molecules. Reproduced with permission from Ref. [151] Copyright 2024, Elsevier. **j** Mechanism of the self-healing process for moisture-triggered self-healing flexible perovskite. Reproduced with permission from Ref. [152] Copyright 2021, Wiley

The introduction of additives to passivate defects, enhance structural robustness, or inhibit water–oxygen corrosion serves as a common strategy for improving the stability of perovskite PDs [145]. The mechanism for enhancing stability through passivation defects is illustrated in Fig. 11a-f, which influence perovskite PDs stability at three levels. Firstly, the incorporation of certain long chains into three-dimensional perovskites can control the material’s crystallization, enhancing its crystallinity or transforming it into quasi-two-dimensional perovskites, which exhibit superior stability, as shown in Fig. 11a, b. This process is analogous to the crystallization control discussed in the previous section. Yan et al. innovatively employed an improved double-sided surface passivation method, utilizing PEAI as additives for Sn–Pb perovskite based on interface engineering defects passivation [146]. PEA^+^ can passivate iodine anion defects at the bottom of the perovskite film. Compared to unilateral passivation, the bilaterally PEA^+^-passivated perovskite layer exhibits superior growth characteristics, such as denser, larger, and more complete perovskite grains, which are conducive to excellent optoelectronic performance. Secondly, certain additives can compensate for inherent defects in the original perovskite structure due to their similar atomic radii, thereby enhancing the stability of the original structure. Zhang et al. reported a method for treating FASnI_3_ perovskite films using a bifunctional CsF additive, which improved film quality and significantly enhanced optoelectronic performance [147]. The improvement stemmed from the Cs substitution and fluoride ion reduction agents in cesium fluoride, which mitigate structural instability caused by abundant Sn vacancy defects and suppress Sn^2+^ oxidation, as shown in Fig. 11c, d, thereby boosting the long-term stability for PDs. Thirdly, the passivation of surface defects by additives enables perovskite films with high density, thereby preventing damage from water molecules and oxygen molecules. Zhai’s group incorporated an ionic liquid, 1-butyl-3-methylimidazolium tetrafluoroborate (BMIMBF_4_) as an additive into MAPbI_3_ nanowires, where the positive electron density of the organic cation [BMIM]⁺ enabled it to acquire electrons from undercoordinated I⁻ and Pb-I inverted defects (PbI_3_⁻), while the high negative electron density of the inorganic anion [BF_4_]⁻ enabled it to incorporate into the MAPbI_3_ lattice structure, compensating for I vacancies and passivating surface unsaturated Pb-related defects in MAPbI_3_, as shown in Fig. 11e [148]. Thus, excellent stability performance without degradation for over 5000 h was realized (Fig. 11f).

For certain perovskite materials prone to oxidation, such as Sn-based perovskites, additives with antioxidant properties have been developed. The primary mechanism for structurally stabilizing involves chelating easily oxidized molecules or inhibiting oxidation through strong reducing agents. Meng’s group introduced different coordinating additives based on this chelation method, such as quercetin (QR). They believed that QR molecules formed multiple coordination bonds with Sn^2+^, so QR molecules chelated with Sn through Sn–O bonds, forming stable five-membered rings [149]. Additionally, due to the multi-site chelation effect, the QR molecules exhibited significant binding energy in their interaction with the FASn_3_ perovskite lattice, indicating strong interaction with the SnI_6_ framework (Fig. 11g). Consequently, they used this method to slow down the crystallization rate of Sn-based perovskites. In Sn-based perovskite materials doped with QR, higher Sn^2+^ content and lower defect concentration demonstrated the effectiveness of the chelation strategy in antioxidant performance. Certain reducing agents that suppress the self-doping effect in Sn-based perovskites, with SnF_2_ as representative, have been demonstrated to effectively inhibit Sn oxidation and enhance device stability. Yun et al. prepared air-stable Pb-free 2D PEA_2_SnI_4_ films with SnF_2_ additive using a nitrogen quenching hot casting method, where SnF_2_ additives could inhibit the transformation form Sn^2+^ to Sn^4+^ and improve the crystallinity of PEA_2_SnI_4_ films [150]. On the one hand, SnF_2_ additives can increase the formation energy of Sn vacancies, thereby preventing the oxidation of Sn^2+^; on the other hand, the addition of SnF_2_ can provide more nucleation sites facilitating more uniform and compact perovskite film growth. Thus, the PEA_2_SnI_4_-based PDs with 20% SnF_2_ exhibited favorable air stability without significant photocurrent decay observed after 300 on–off cycles, as shown in Fig. 11h.

The humidity stability of perovskite materials also warrants investigation, as it facilitates the application of perovskite PDs in more scenarios, such as underwater detection. Significant efforts based on additive engineering have been developed to enhance the water stability of perovskites. Constructing hydrophobic pathways within or on the surface of perovskite materials is a common approach that reduces contact between the material and water molecules, thereby improving stability. Yang et al. have constructed a new class of (TzBIPY)Pb_2_X_6_ (X = Cl, Br, I) perovskites using the 2,5-di(pyridin-4-yl)thiazole-fused [5,4-D]thiazole (TzBIPY^2+^) cation as the organic cation [151]. The hydrophobic TzBIPY^2+^ cation effectively encapsulates the inorganic Pb-X framework through intermolecular interactions, including hydrogen halide bonds, π–π overlap, and π–cation interactions, as shown in Fig. 11i. As a result, the (TzBIPY)Pb_2_Br_6_ hybrid perovskite exhibited excellent stability over 3000 h in polar solvents such as MeOH and γ-butyrolactone as well as in water. Utilizing additional additives to adsorb water molecules and employing these molecules for perovskite healing also represent a promising strategy. Wang et al. fabricated a self-healing perovskite film that repairs mechanical damage by absorbing moisture from the surrounding environment [152]. The designed polyvinyl alcohol (PVA) micro-scaffold not only served to stabilize the black phase of the perovskite film but also endowed it with self-healing capabilities in humid environments. PVA, as a hydrophilic material, readily absorbed water and, through strong interactions with water molecules, displaced it away from the perovskite. Additionally, water molecules on the PVA surface activated hydroxyl groups, enabling PVA to self-heal through hydrogen bond reconstruction between hydroxyl groups while maintaining excellent flexibility, as shown in Fig. 11j. Consequently, it alleviated cracks and associated defects in the perovskite caused by repeated mechanical deformation, achieving outstanding water stability.

### Encapsulation Technology

Beyond additive engineering, the introduction and advancement of encapsulation technology can effectively improve stability and longevity of perovskite PDs without altering devices performance. For perovskite encapsulation, high-performance encapsulation materials and technological requirements should meet the following criteria: (a) good mechanical insulation properties, (b) high light transmittance, (c) effective barrier against moisture and oxygen penetration into the device, and (d) absence of damage to the perovskite material [153]. Based on the distinction of encapsulation regions, perovskite technology can be categorized into external encapsulation and internal encapsulation. The former involves encapsulation on the external side of the device, while the latter entails encapsulation at the grains level within the perovskite material itself [154].

**Fig. 12:**
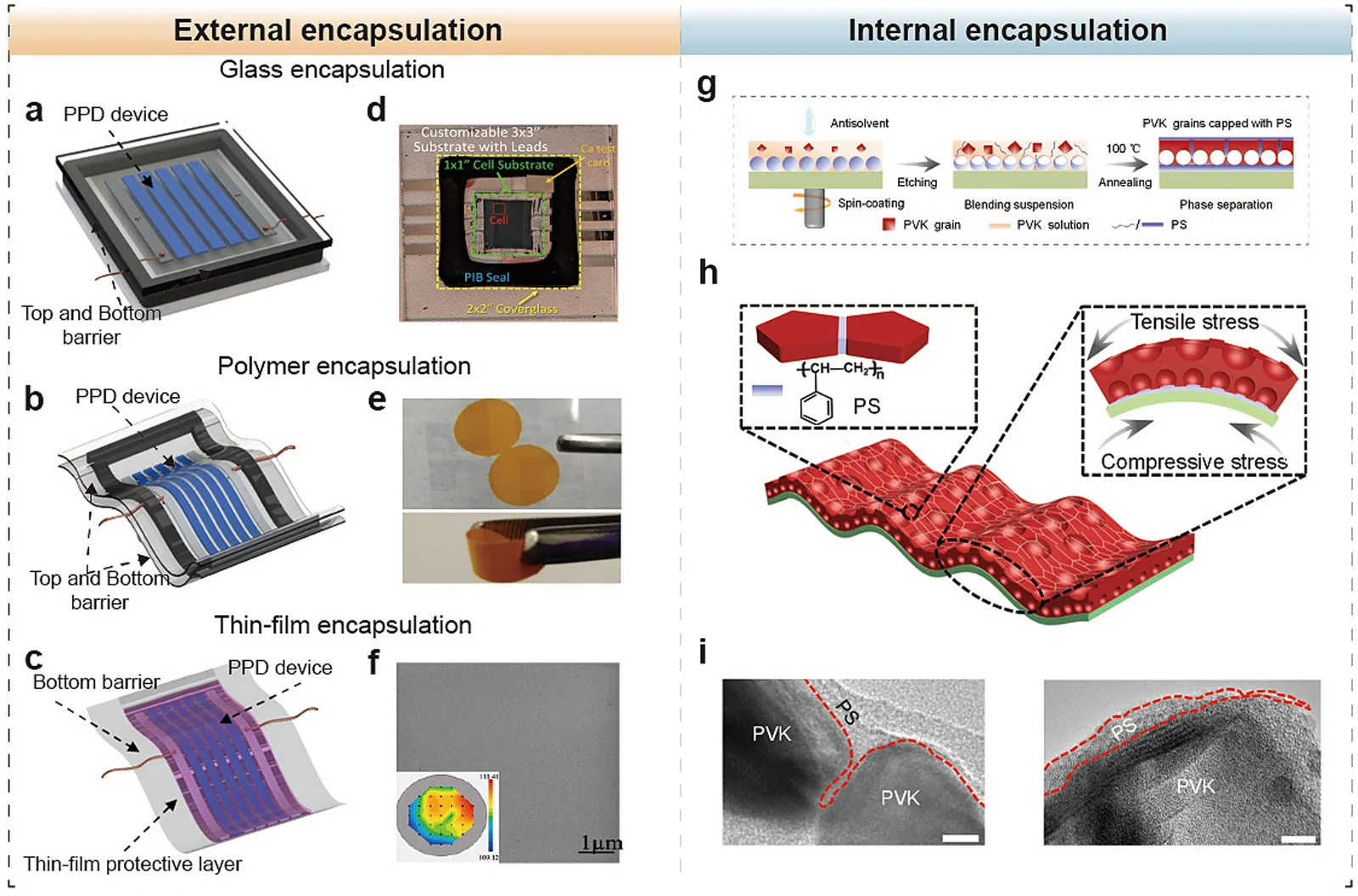
Encapsulation technologies for perovskite PDs. Schematic diagram of **a** glass encapsulation, **b** polymer encapsulation and **c** thin-film encapsulation. Reproduced with permission from Ref. [154] Copyright 2021, Wiley. Representative examples for **d** glass encapsulation, Reproduced with permission from Ref. [155] Copyright 2022, American Chemical Society; **e** polymer encapsulation, Reproduced with permission from Ref. [156] Copyright 2024, Wiley; **f** thin-film encapsulation. Reproduced with permission from Ref. [157] Copyright 2024, Elsevier. **g** Fabrication procedure of HG-PC perovskite-based PD. **h** Schematic diagram of the hierarchical HG-PC photonic and “brick-and-mortar” structure. **i** SEM image of PVK-encapsulated perovskite. Reproduced with permission from Ref. [158] Copyright 2022, Elsevier

External encapsulation methods originate from commercial encapsulation techniques in the perovskite photovoltaic industry, primarily including glass encapsulation (Fig. 12a, d), polymer encapsulation (Fig. 12b, e), and thin-film encapsulation (Fig. 12c, f). Glass encapsulation is a mature technical solution in the photovoltaic industry that effectively protects against corrosion from oxygen and moisture. However, this encapsulation process has a significant negative impact on thermosensitive perovskite materials. Furthermore, rigid glass substrates are incompatible with flexible electronic devices manufacturing, severely limiting the application potential of perovskite PDs in the large-area flexible devices production. Alternatively, polymer encapsulation technology has garnered significant attention due to its advantages of low cost, easy accessibility, and material selection flexibility. Materials employed in such technologies include PMMA, polyethylene terephthalate (PET), polytetrafluoroethylene (PTFE), and ethylene–vinyl acetate copolymer. Thin-film encapsulation consists of a multilayer structure incorporating both inorganic and organic layers, offering a promising solution for perovskite encapsulation. The inorganic layer determines the barrier protection, while the organic layer enhances flexibility by mitigating interfacial stress between brittle inorganic layers, thereby ensuring device reliability. However, the reaction temperatures of commonly employed physical or chemical vapor deposition techniques in thin-film encapsulation, such as plasma-enhanced chemical vapor deposition and atomic layer deposition, pose a risk of damaging perovskite materials. This issue warrants future attention and resolution.

Employing external encapsulation technology to prevent moisture and oxygen permeation serves as a simple and feasible process. However, external encapsulation cannot effectively address irreversible light-induced degradation stemming from various vacancies and ion diffusion or migration within the device itself. Consequently, the strategy of internal encapsulation has been proposed, which essentially represents a further refinement of additive engineering. Li’s group reported a self-encapsulation strategy that exemplifies internal encapsulation [158]. A method was proposed for fabricating flexible perovskite films using solvent-assisted self-assembly of polymers combined with imprinting technology during crystallization. This approach enabled the simultaneous formation of polymer-bonded perovskite grains, resulting in a pearlescent “brick-and-mortar” structure, as shown in Fig. 12g. As presented in Fig. 12h, i, two perovskite grains were bonded by polystyrene (PS), with the perovskite grain surfaces covered by a thin layer of polymer, confirming the formation of PS-bonded perovskite heterostructures and effectively establishing a water–oxygen-resistant barrier at the granular level.

This section provides a systematic summary of approaches to improving the stability for perovskite PDs, including optimizing crystal quality, additive engineering, and encapsulation strategies. Crystallization control suppresses grain boundaries and intrinsic defects prone to degradation at the thermodynamic root; additive engineering constructs a hydrophobic network while in situ passivating traps; and encapsulation technology serves as a final and robust physical barrier, blocking the pathways of environmental water and oxygen as well as dynamic stress. Still, as mentioned in the previous sections, the challenge for perovskite PDs does not lie in achieving extreme and singular stability enhancement, but rather in maximizing their operational lifespan to meet the specific application needs while preserving their excellent properties (such as inherent flexibility and high light-to-electricity conversion efficiency).

**Fig. 13:**
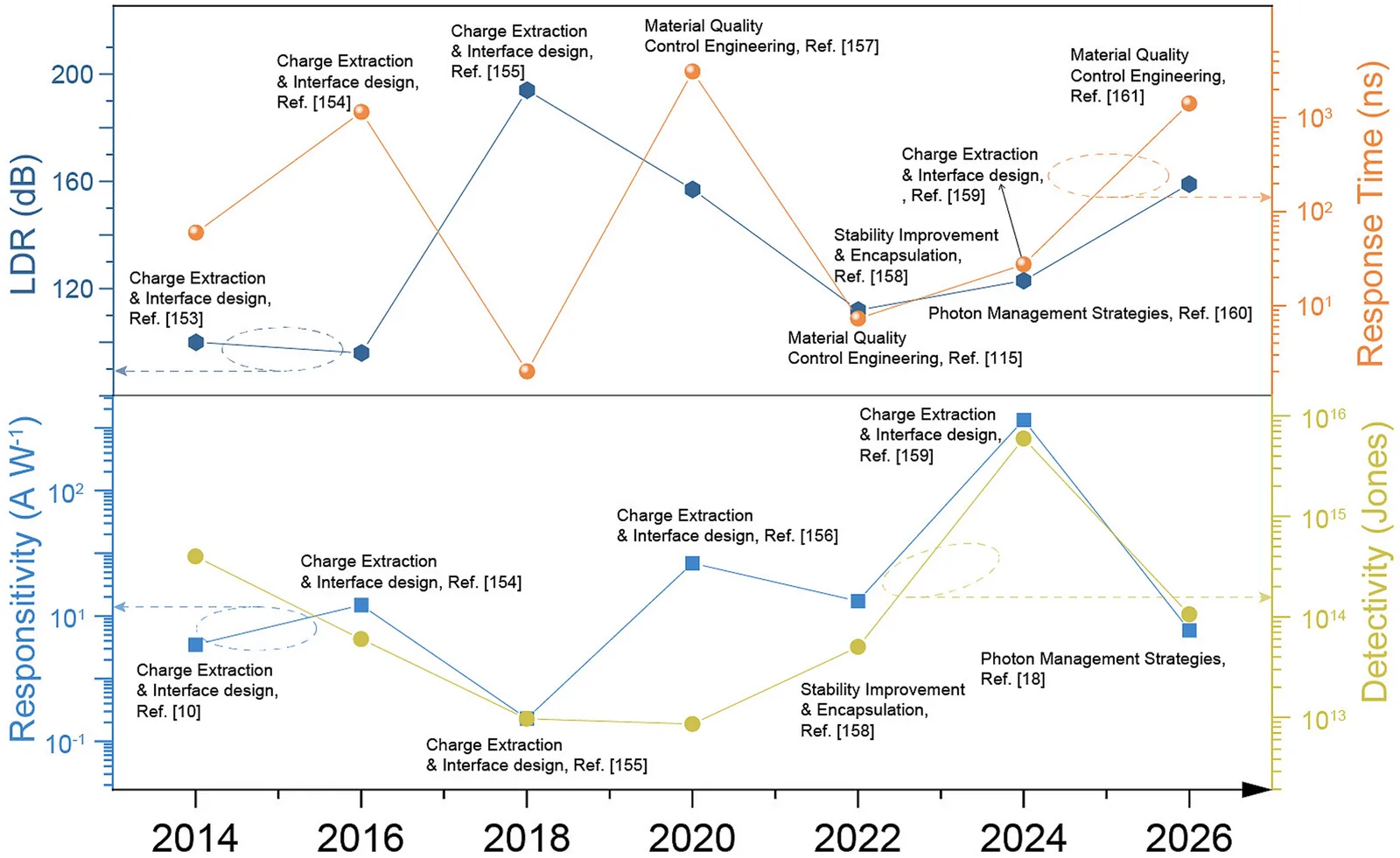
Performance evolution diagram over the past decade illustrates the relationship between optimization strategies and performance improvements for perovskite PDs [10, 18, 115, 159–167]

The above four sections summarize the core optimization strategies on device level for perovskite PDs by following a systematic approach that covers the entire workflow, including materials preparation, optical management, interfacial charge collection, and stability enhancement. The recent work on perovskite PDs performance optimizing utilizing the aforementioned optimization strategies is summarized in Table 1, with the key metrics of these devices compiled in the table. Over the past decade, these strategies have focused on improving various performance metrics of perovskite PDs, leading to significant advancements as shown in Fig. 13. Through the synergistic optimization of photophysical processes, electrical dynamics, and material chemical stability, significant breakthroughs can be realized to overcome the performance bottlenecks in PD sensitivity, response speed, and reliability, thereby laying a solid device foundation for the practical application of multifunctional sensing systems. The performance concerns and optimization strategies for different applications are summarized in Table 2 to clarify their relationships and trade-offs.

**Table 1:** Performance summary of perovskite PDs and commercialized silicon PDs

	Structure	Bias (V)	Dark currents (A)	Responsivity (A^−1^ W)	Specific detectivity (Jones)	Response speed	LDR (dB)	Stability
Si PDs	PIN	2	100 pA	0.25	\	2000 MHz	\	\
	PN	0	20 pA	0.4	\	0.5 μs	\	\
	APD	150	0.1 nA	0.5	\	1000 MHz	\	\
Perovskite photoconductor	Au/Cs_3_Bi_2_X_9_ SC/Au [16]	10	4.12 × 10^–14^	0.0231	7.37 × 10^11^	\	\	\
	Au/(C_6_H_5_(CH_2_)_2_NH_3_)_2_PbI_4_ NW/Au [17]	5	2 × 10^–10^	0.49	1.21 × 10^13^	78/105 μs	\	3000 bending cycles
	Au/black-phase γ-CsPbI_3_/Au [168]	\	2.9 × 10^–11^	42.1	1.3 × 10^14^	290/350 µs	112	192 h
	Ag/PEA_2_MA_n-1_Pb_n_I_3n+1_/Ag [109]	1	3.5 × 10^–11^	26	3.25 × 10^15^	\	147	after 40 days
	Au/(C_12_H_27_N)_2_(MA)_n-1_(Pb)_n_(Br)_3n+1_/Au [169]	4	\	0.183	2.9 × 10^11^	3.4/4.3 ms	43.8	\
	Au/CsPbX_3_/Au [20]	\	\	69.88	3.27 × 10^15^	100/60.1 ms	50	7 days
	Au/BTMA_2_PbI_4_/Au [170]	1	0.04 nA	314	3.4 × 10^13^	440/530 μs	\	5000 s
	Au/(TzBIPY)Pb_2_X_6_/Au [151]	2	2.86 × 10^–10^	\	\	96/158 ms	97	3000 h
	ITO/Al_2_O_3_/CsPbCl_3_/Au [171]	\	1 × 10^–10^	0.285	7 × 10^12^	2.1/5.5 μs	95	140 h
	Au/CsPbCl_3_/CsPbI_3_/Au [172]	5	\	49	1.51 × 10^13^	13/16 ms	\	\
	Au/Rb_2_CuCl_3_ MWs/Au [173]	3	\	0.078	0.27 × 10^12^	50/220 ms	\	60 days
	Au/nanograting-structured MAPbBr_3_/UCNPs/Au [91]	1	\	2.9	1.34 × 10^11^	\	\	124 days
	Au/2D Perovskite Nanocrystals/Au [64]	5	\	1.5 × 10^6^	1.1 × 10^16^	22.1 ms	\	204 h
	ITO/((Cs_0.06_FA_0.79_MA_0.15_)Pb(I_0.85_Br_0.15_)_3_):(SiO_2_/Ag/SiO_2_)/ITO [174]	3	\	1880	2.2 × 10^12^	600/650 μs	\	\
Perovskite photoconductor	ITO/MAPbX_3_-MNPs/ITO [89]	5	\	171.1 (410 nm), 269.1 (550 nm), 452.7 (830 nm)	1.26 × 10^14^ (410 nm), 1.84 × 10^14^ (550 nm), 3.13 × 10^14^ (830 nm)	\	\	\
	Au/Cu(GABA)_2_PbBr_4_/Au [108]	5	10^–14^	1.66	6.19 × 10^12^	\	\	\
	Au/PMA_2_PbI_4_/Au [175]	5	6.78 × 10^–12^	0.64 (488 nm), 1.02 (532 nm)	2.87 × 10^12^ (488 nm), 4.51 × 10^12^ (532 nm)	121/154 μs	130	4h and 500 bending cycles
	Au/CsPb(Br_1-x_I_x_)3/Au [176]	0.5	\	4.24	\	18.7/25.1 ms	\	\
Perovskite photodiode	ITO/PEDOT:PSS/(tBBA)_2_MA_2_Pb_3_I_10_/P3HT/Ag [177]	0	\	\	\	19.7/18.3 ns	\	\
	ITO/SnS_2_/SnO_2_/MAPbI_3_/P3HT/Ag [178]	0	1 × 10^–8^	0.4	9 × 10^12^	\	\	\
	PEN/ITO/PEDOT:PSS/SCPU-PVK/Ag [179]	0	\	0.4	2.5 × 10^11^	2 μs	\	9000 bending cycles
	ITO/SnO_2_/(PEA)_2_MA_3_Pb_4_I_13_/P3HT/Ag [180]	0	\	0.137	\	7 µs	\	\
	ITO/MeO-2PACz/Cs_0.05_[(FA)_0.83_(MA)_0.17_]_0.95_Pb(I_0.9_Br_0.1_)_3_/BHJ/C60/BCP/Ag [181]	0.5	5.8 × 10^–8^ A cm^−2^	0.44	4.7 × 10^12^	7.7/12.3 µs	118	800 cycles
	ITO/Cs_0.1_FA_0.87_GA_0.03_Pb_1-x_Sn_x_(I_0.95_Br_0.05_)_3_/Ga [182]	1	\	0.23	1.3 × 10^12^	34.7/81.2 µs	117	Over a year
	ITO/PEDOT:PSS/PTAA/Al_2_O_3_/(PEA)_2_(MA)_3_Pb_4_I_13_/PC_61_BM/BCP/Ag [183]	0	9.62 × 10^–11^	0.35	8.36 × 10^12^	0.43/4.13 µs	171.4	70 h
Perovskite photodiode	ITO/2D PEA_2_PbI_4_/SnO_2_/Ag [15]	0	4 × 10^–7^ A cm^−2^	2.12	\	160/128 ns	66	150 days
	FTO/SnO_2_/porous MAPbI_3_/FAPbI_3_ QDs/spiro-OMeTAD/Ag [184]	0	\	1.37	1 × 10^14^	6.6/7.9 µs	118.7	700 h
	ITO/SnO_2_/PbBr_2_-CsBr/CsPbBr_3_/conductive carbon paste [185]	0	1.8 × 10^–11^	0.35	1.25 × 10^13^	1.41/2.06 μs	175	\
	ITO/PEDOT:PSS/FASnI_3_:CNI/PCBM/C60/PFN-Br/Ag [136]	0	1.04 × 10^–9^	0.4	2.2 × 10^13^	2.62/5.91 µs	\	10,000 s
	ITO/PEDOT:PSS/PEA_0.15_FA_0.85_SnI_3_/EDA/CB/PCBM/C60/BCP/Ag [186]	0.1	1.39 × 10^–7^ A cm^−2^	0.34	1.6 × 10^12^	530 ns	45	\
	ITO/NiO_x_/PTAA/Cs_0.15_FA_0.85_Pb_0.5_Sn_0.5_I_3_/PCBM/BCP/Ag [187]	0	\	0.5	1.04 × 10^12^	61/30 µs	155	\
	ITO/SnO_2_/FASnI_3_ QSC film/NPB/Cu [188]	0.1	\	0.554	1.0 × 10^10^	130/130 ms	\	\
	ITO/SnO_2_/FA_0.9_Cs_0.1_PbI_1_Br_2_ /BHJ/MoO_x_/Cu [189]	0.4	3.41 × 10^–9^ A cm^−2^	\	1.86 × 10^11^	\	> 190	1000 cycles
	FTO/ETL/Cs_0.05_(MA_0.1_FA_0.9_)_0.95_Pb(I_0.9_Br_0.1_)_3_/Spiro-OMeTAD/Ag [190]	0	1.5 × 10^–10^ A cm^−2^	0.93	1.55 × 10^12^	0.3/25.5 ms	\	6000 s and bending 300 times
Perovskite photodiode	ITO/SnO_2_/FAPbI_3_ PQDs ink/Spiro-OMeTAD/Ag [191]	0	1.23 nA cm^−2^	0.38	5 × 10^12^	2.5/2.2 µs	\	\
	ITO/PEDOT:PSS/APV/Perovskite/PC_61_BM/Anth-hpp2/Ag[192]	0.5	5.75 × 10^–10^ mA cm^−2^	0.28	2.08 × 10^13^	256/438 ns	168	\
	FTO/PEDOT: PSS/MAPbI_2_Br/Au [193]	0	\	0.179	2.6 × 10^10^	1.8/48.7 µs	116	\
	Ag/(PEA)_2_PbI_4_/ZnPc/Ag [194]	0	1.5 × 10^–14^	32	3.14 × 10^15^	337/366 μs	111	\
	ITO/MeO-2PACz//Cs_0.05_(FA_5/6_MA_1/6_)_0.95_Pb(I_0.85_Br_0.15_)_3_/C60/BCP/Ag [195]	0.5	2.4 × 10^–9^ A cm^−2^	0.46	8.1 × 10^12^	133 ns	117	400 cycles
	ITO/NiO_x_-based HTL/(PEA)_2_FA_4_Pb_5_I_16_/PCBM/BCP/Ag [117]	0	\	0.325	3.15 × 10^10^	1.6 ms/235 µs	94.8	3750 cycles
	ITO/CL-PTAA/Cs_0.15_FA_0.85_PbI_3_/ETL/cathode buffer layer/Ag [112]	0	\	0.53	3.58 × 10^14^	15.7/7.8 µs	217	13,653 h
	ITO/PEDOT:PSS/FA_0.85_Cs_0.15_Pb_0.5_Sn_0.5_I_3_/PCBM/BCP/Ag [105]	0	\	0.47 (660 nm), 0.53 (860 nm)	4.6 × 10^12^	93.2/185.7 ns	145	2400 h
	ITO/PEDOT:PSS/FA_0.85_PEA_0.15_Sn_1-x_Pb_x_I_3_ perovskite/PCBM/BCP/Ag [196]	\	1.2 × 10^–8^ A cm^−2^	0.405	8.86 × 10^12^	684/683 ns	\	\
	PET/ITO/PEDOT:PSS/CsBi_3_I_10_/PC_61_BM/Au [197]	0	1.07 × 10^–8^	0.56	4.51 × 10^11^	15/1.1 μs	\	11 h
Perovskite photodiode	FTO/TiO_2_/PS/FASnI_3_-BDADI IO/P3HT/Ag [18]	0	3.9 × 10^–8^	5.9	1.06 × 10^14^	14.1/25.3 µs	\	70 days
	FTO/PEDOT: PSS/Cs_2_PbBr_2_I_2_/PCBM/Ag [198]	0	10^–9^ A cm^−2^	0.87	10^11^	300/101 µs	80.32	1200 s
	ITO)/MeO-6PACz/Sn–Pb perovskite/C60/BCP/Ag [199]	0	5.4 × 10^–10^ A cm^−2^	0.5	1.42 × 10^11^	377/860 ns	173	120 min
	ITO/MeO-2PACz/FA_0.55_MA_0.4_Cs_0.05_Sn_0.5_Pb_0.5_I_3_/C60/BCP/Cu [200]	0	3.5 × 10^–9^ A cm^−2^	0.51	3.6 × 10^12^	181/188 µs	96	25,000 cycles
	ITO/PEDOT:PSS/Sn–Pb perovskite with Sn(SCN)_2_/C60/BCP/Ag [22]	1	10 nA cm^−2^	0.58	1.6 × 10^13^	0.48/0.34 µs	147	14 days
Perovskite phototransistor	Au/(F-PEA)_2_PbI_4_/Au [201]	7.5	\	1100	5 × 10^17^	1.5 ns	228	1000 bending cycles
	Au/MAPbBr_3_/Au [202]	\	\	3700	\	55/35 μs	\	180 days
	Au/(PEA)_2_PbI_4_/Au [203]	1	10^–11^	2.02	5.8 × 10^11^	38.5/64.2 ms	\	\

**Table 2:** Performance improvements and optimization strategies on perovskite PDs for different applications, clarifying their relationships and trade-offs

	Structure		Optimization strategies	Performance improvements	Application	Ref
Material quality control engineering	Photoconductor	Au/Cs_3_Bi_2_X_9_ SC/Au	Large-area high-quality single-crystal growth	Dark currents (40 fA)	Optical imaging	[16]
	Photoconductor	Au/(C_6_H_5_(CH_2_)_2_NH_3_)_2_PbI4 NW/Au	Vacuum-assisted space-limiting methods	Stability (3000 bending cycles)	Flexible imaging	[17]
	Phototransistor	Au/(F-PEA)_2_PbI_4_/Au	Solvent-resistant vapor-assisted single crystals growth	Responsivity (1100 A^−1^ W) response speed (1.5 ns)	Flexible imaging	[201]
	Phototransistor	Au/MAPbBr_3_/Au	Self-limiting CVD single crystal growth method	Responsivity (3700 A-1 W) stability (180 days)	Image imaging	[202]
	Photodiode	ITO/NiO_x_-based HTL/(PEA)_2_FA_4_Pb_5_I_16_/PCBM/BCP/Ag	Thermal regulation method	Stability (3750 cycles)	NIR range and weak-light imaging	[117]
	Photodiode	PET/ITO/PEDOT:PSS/CsBi_3_I_10_/PC_61_BM/Au	Precise control of crystallization kinetics	Response speed (15/1.1 μs)	Wearable health monitoring	[197]
	Photoconductor	FAPbI_3_ SC rods	High-quality perovskite single-crystal rods growth	Sensitivity (2.16 × 10^5^ µC Gy^−1^ cm^−2^) detection limit (2 nGy s^−1^)	X-Ray detection and imaging	[204]
	Photodiode	Au/(PEA)_2_PbBr_4_/CsPbBr_3_/Au	Low-temperature liquid-phase epitaxy for high-quality single crystals growth	Detection limit (3.05 nGy s^−1^)	X-ray detection	[205]
Photon management strategies	Photodiode	ITO/SnS_2_/SnO_2_/MAPbI_3_/P3HT/Ag	Optical management and interface engineering	Specific detectivity (9 × 10^12^ Jones)	Multispectral imaging	[178]
	Photodiode	ITO/ 2D PEA_2_PbI_4_/SnO_2_/Ag	Light trapping with planar nanocavities	Response speed (160/128 ns)	Visible light communications	[15]
	Photoconductor	Au/nanograting-structured MAPbBr_3_/UCNPs/Au	Light conversion using dual-nanograting and upconversion particles	Expanded spectral range (from Vis to NIR)	NIR flexible detection	[91]
	Photoconductor	ITO/MAPbX_3_-MNPs/ITO	Plasmon-mediated	Specific detectivity (3.13 × 10^14^ Jones)	Wavelength-selective detection	[89]
	Photodiode	FTO/TiO_2_/PS/FASnI_3_-BDADI IO/P3HT/Ag	Photonic crystal	Response speed (14.1/25.3 µs)	NIR optical communication	[18]
Charge extraction and interface design	Photoconductor	FTO/(HtrzT)PbI_3_/FAPbI_3_/Au	Additive engineering	Sensitivity (1.83 × 10^5^ μC Gy^−1^ cm^−2^)	X-ray detection and imaging	[206]
	Photoconductor	FTO/(PBL)_4_PbI_6_-MAPbI_3_/FTO	Carrier extraction via1D/3D heterostructures	sensitivity (76,553 μC Gy^−1^) detection limit (9.5 nGy s^−1^)	X-ray detection and imaging	[19]
	Photodiode	ITO/MeO-2PACz/Cs_0.05_[(FA)_0.83_(MA)_0.17_]_0.95_Pb(I_0.9_Br_0.1_)_3_/BHJ/C60/BCP/Ag	Heterostructure design	Dark current (5.8 × 10^–8^ A cm^−2^)	Heartbeat monitoring	[181]
	Photoconductor	Au/CsPbX_3_/Au	Controllable gradient components and gradient-controlled bandgap	specific detectivity (3.27 × 10^15^ Jones)	Color recognition and imaging	[20]
	Photodiode	ITO/SnO_2_/FAPbI_3_ PQDs ink/Spiro-OMeTAD/Ag	Surface passivation and regulating potential barriers	Dark current (1.23 × 10^–9^ cm^−2^) response speed (2.5/2.2 µs)	Health monitoring	[191]
	Photoconductor	Au/2D Perovskite Nanocrystals/Au	Liquid-bridge transport and droplet-pinned crystallization	Responsivity (1.5 × 10^6^ A W^−1^) specific detectivity (1.1 × 10^16^ Jones)	Intelligent vision for shape recognition	[64]
	Photodiode	ITO/PEDOT:PSS/APV/Perovskite/PC_61_BM/Anth-hpp2/Ag	Surface engineering	Response speed (256/438 ns) LDR (168 dB)	Optical communications	[192]
	Photodiode	Ag/(PEA)_2_PbI_4_/ZnPc/Ag	Heterojunction design	Dark current (15 fA) specific detectivity (3.14 × 10^15^ Jones)	Acousto-optic conversion communication	[194]
	Photodiode	ITO/]CL-PTAA/Cs_0.15_FA_0.85_PbI_3_/ETL/cathode buffer layer/Ag	Facile interface engineering	Specific detectivity (3.58 × 10^14^ Jones) LDR (217 dB) stability (13,653 h)	Versatile photosensing	[112]
Stability improvement and encapsulation	Photodiode	PEN/ITO/PEDOT: PSS/SCPU-PVK/Ag	Additive engineering	Stability (9000 bending cycles)	Flexible PDs	[179]
	Photoconductor	Ag/PEA_2_MA_n-1_Pb_n_I_3n+1_/Ag	Interface passivation	Dark current (35 fA) specific detectivity (3.25 × 10^15^ Jones)	Weak-light imaging	[109]
	Photoconductor	ITO/Al_2_O_3_/CsPbCl_3_/Au	Passivation layer thickness optimization	Response speed (2.1/5.5 μs) stability (40 days)	Solar ultraviolet radiation monitoring	[171]
	Photodiode	ITO/PEDOT:PSS/FASnI:CNI/PCBM/C60/PFN-Br/Ag	Additive engineering	Response speed (2.62/5.91 µs)	Wearable health monitoring and weak light imaging	[136]
	Photodiode	ITO)/MeO-6PACz/Sn–Pb perovskite/C60/BCP/Ag	Additive engineering	Dark current (5.4 × 10^–10^ A cm^−2^) response speed (377/860 ns) LDR (173 dB)	NIR imaging	[199]
	Photodiode	ITO/PEDOT:PSS/Sn–Pb perovskite with Sn(SCN)2/C60/BCP/Ag	Surface passivation strategy	stability (14 days)	NIR imaging	[22]
	Photodiode	Au/MAPbBr_3_/C60/Cr	Epoxy encapsulation	Sensitivity (1.55020 µC Gy^−1^ cm^−2^)	Underwater X-ray imaging	[207]
	Photoconductor	Au/MAPbI_3_ SC: Bvim-TFSI/Au	In situ ionic liquid oligomer encapsulation	Sensitivity (1.80 × 10^5^ μC Gy^−1^ cm^−2^)	X-Ray detection and imaging	[207]

## Application and Research Progress of Perovskite PDs

**Fig. 14:**
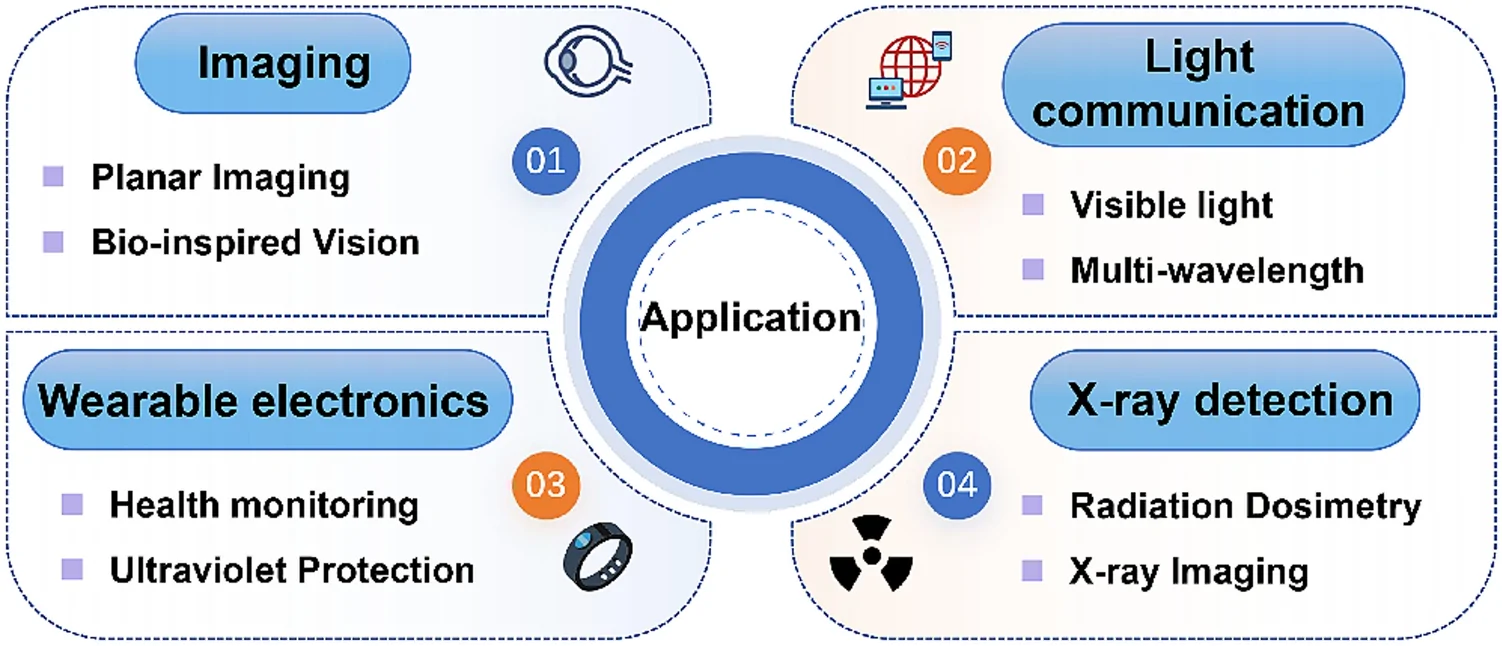
A schematic diagram illustrating the framework of perovskite PD applications discussed in this review

Perovskite PDs, with their exceptional performance across a broad spectral range, combine lightweight and flexible characteristics. Compared to conventional PDs, the perovskite PDs offer distinct advantages such as wider light detection ranges, higher sensitivity, and outstanding external quantum efficiency, allowing for broader application scenarios [208, 209]. The perovskite PDs exhibit distinct application advantages in various scenarios depending on device structure. Photodiodes typically feature faster response speed and self-powering, particularly suitable for scenarios requiring high-speed transmission such as optical communications and offline signal monitoring. Photoconductors and phototransistors, while offering higher response sensitivity, exhibit slower response speeds and are preferred for applications such as optical control switches. This section will focus on their potential applications in imaging, light communications, wearable electronics, and X-ray detection, as shown in Fig. 14.

### Imaging

Image imaging is a fundamental function that transforms optical information into electrical signals, serving as the core of numerous devices including digital cameras, smartphones, security surveillance systems, and medical equipment [210]. In recent years, research on imaging technologies based on perovskite materials has been extensively conducted, and planar image sensors employing various measurement methods have been successfully realized. Furthermore, inspired by the diversity of biological visual systems in nature, the development of bio-inspired vision has introduced capabilities such as an ultra-wide field of view (FoV), high spectral resolution, and excellent environmental adaptability, which are unattainable in planar imaging. Meanwhile, in-sensor visual computing systems inspired by biological synapses and neural networks have been intensively investigated to enhance image processing efficiency. These emerging optoelectronic devices are no longer limited to traditional light signal detection but are evolving toward multifunctional integration. For example, some PDs can directly perform analog or digital computing tasks while sensing light signals, such as simulating optically controlled synaptic plasticity, image preprocessing, or matrix–vector multiplication. Others possess the capability of optical writing and electrical reading, enabling non-volatile optical memory functions. This photodetection architecture that integrates sensing, computing, and memory into a single device offers new possibilities for constructing intelligent optoelectronic systems with integrated sensing, memory, and processing capabilities.

Advances in image sensing technology have directly benefited from high-quality thin-film growth, interface passivation, and bandgap engineering. However, optimization strategies vary depending on the imaging paradigm. For traditional large-scale planar imaging arrays that prioritize high resolution and extreme uniformity, the core strategy lies in eliminating inter-device variations. Through coordinated chemical interface passivation, dark noise variations between pixels caused by random grain boundaries are eliminated. In contrast, for future-oriented bio-inspired imaging, the core strategy shifts toward the active utilization of carrier dynamics. Through precise bandgap engineering, involving the tailored introduction, custom design of specific trap states or asymmetric bandgap gradients, devices can leverage trap-assisted grating effects or ion–electron coupling behavior to simulate the preprocessing functions of biological retina such as local feature extraction and dynamic environmental adaptation. This section will discuss the latest advances in perovskite-based detectors for imaging applications, focusing on both planar imaging and bio-inspired vision.

#### Planar Imaging

Based on pixel configuration, planar imaging technology is defined by two main approaches, comprising single-point imaging and array imaging. Single-point imaging offers advantages in spectral selectivity, sensitivity, and resolution accuracy [211]. In contrast, array imaging is compatible with conventional silicon-based sensors but requires large-area, high-pixel-density PD arrays to achieve full camera functionality [212]. These two imaging technologies exhibit distinct strengths across different application domains, thereby imposing divergent technical requirements on perovskite PDs. Moreover, it is noteworthy that the tunable bandgap of perovskite materials enables their optical response to span from UV to NIR spectra for advanced detection and imaging. Currently, various types of perovskite PDs have demonstrated extensive imaging applications across UV, visible, and NIR spectral regions. This section surveys the applications of perovskite PDs across spectral bands through examinations of both single-point and array imaging configurations.

I. Single-point imaging

**Fig. 15:**
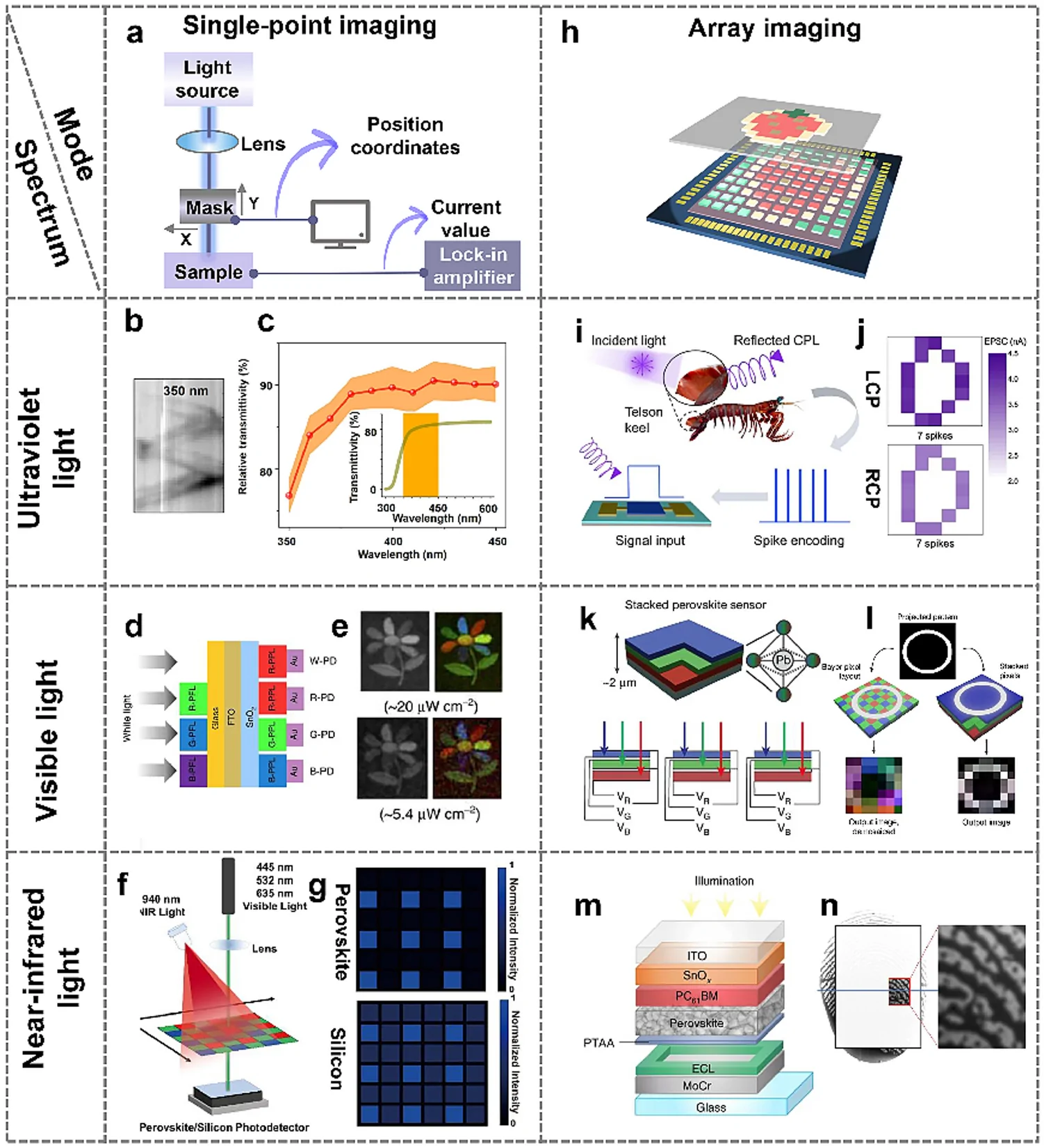
Applications of perovskite PDs in planar imaging. **a** Schematic of the single-point imaging method. **b** Imaging results of a letter “W” patterned on glass substrate using TiO_2_ paste at 350 nm. **c** Calculated transmission spectra from the obtained hyperspectral imaging results, inset is the spectrum of TiO_2_ measured by a conventional spectrophotometer. Reproduced with permission from Ref. [213] Copyright 2022, Springer Nature. **d** Device structure of the perovskite-based PD. **e** Imaging results of the proposed perovskite-based color camera under different light intensities. Reproduced with permission from Ref. [214] Copyright 2023, Springer Nature. **f** Schematic of imaging equipment. Three colors of lasers are used as the target light, and 940 nm LEDs are used as interference light. **g** Color reproduction ability of perovskite PDs and Si PD. Reproduced with permission from Ref. [182] Copyright 2024, Springer Nature. **h** Schematic of the array imaging method. **i** Schematic diagram of CPL imaging. **j** Measured weights of the target images after 395-nm LCP/RCP optical spike stimuli. Reproduced with permission from Ref. [215] Copyright 2023, Springer Nature. **k** Schematic of the designed stacked perovskite sensor. **l** A comparative experiment of imaging a black-and-white ring pattern (top) was conducted, comparing results from a Bayer CFA imager after demosaicing (left) with those from a stacked perovskite imager (right). Reproduced with permission from Ref. [33] Copyright 2025, Springer Nature. **m** Schematic of the designed discrete PD. **n** Fingerprint image obtained with the image sensor. Reproduced with permission from Ref. [216] Copyright 2021, Springer Nature

Figure 15a illustrates the schematic diagrams of the single-point imaging approaches, comprising a light source, a patterned photomask, and a PD. In this setup, the PD functions as a point-like detector to capture optical signals, while the photomask featuring hollow patterns is mounted on a computer-controlled two-dimensional translation stage. This stage moves continuously along a meandering path through the beam focus. The photocurrent signals generated by light excitation through the hollow patterns are collected by a lock-in amplifier synchronized with the translation stage under computer control.

*Ultraviolet light single-point imaging*. At present, environment-friendly, fully inorganic, lead-free perovskite wide bandgap materials (≥ 3.1 eV), including halogenated double perovskites [217], copper-based perovskites [218], and bismuth-based perovskites [219], are widely recognized as ideal candidate materials for UV imaging sensors due to their highly efficient UV light absorption, outstanding optoelectronic properties, and stability, which are employed extensively in life sciences, environmental monitoring, and aerospace. For example, Cs_3_Bi_2_Br_9_ exhibits a wide range of optical bandgaps spanning 2.5 − 3.3 eV due to various synthesis techniques, crystalline quality, and crystal orientation, endowing them with potential applications in UV imaging [220]. Liu et al. developed a self-powered NiO_x_/Cs_3_Bi_2_Br_9_ heterojunction PD that exhibits exceptional UV multispectral imaging capabilities in the 350–400 nm wavelength range [213].] Based on the principle that TiO_2_ paste coatings absorb UV light resulting in low transmittance, the authors employed a single-point imaging approach to demonstrate remarkable imaging performance for the letter “*W*” pattern (Fig. 15b). They further processed the spectral data (λ) from the TiO_2_-coated regions and extracted transmittance curves measured by the device. These results showed strong consistency with the transmittance spectra of TiO_2_ thin films obtained using a conventional spectrophotometer (Fig. 15c), thereby confirming the multispectral imaging capability of the optimized NiO_x_/Cs_3_Bi_2_Br_9_ heterojunction PD. This single-point imaging technique struggles to yield meaningful images from natural scenes. The researchers adopted an alternative approach, employing structured light as a spatial information carrier and utilizing PDs lacking spatial resolution as signal collectors to capture scenes [221]. In other words, in the field of diffuse light imaging, what is truly required at this stage is not an array-based PD camera, but rather a single PD. Early single-point imaging, also termed ghost imaging, primarily employed random pattern illumination. This technique typically requires combining photodetection results with optimization algorithms to enhance image quality, such as normalized ghost imaging and Fourier-based single-point imaging [212]. Such approaches demand the PD capable of perceiving minute variations in diffuse light, which is even more demanding than for conventional cameras.

*Visible spectrum single-point imaging*. Perovskite PDs for the visible spectrum imaging have been extensively reported; notably, in the visible light spectrum, perovskite PDs exhibit specific responses to red, green, and blue light, analogous to the photoreceptor cones in the human retina, thereby demonstrating advantages in full-color imaging. Conventional image sensors based on inorganic PD arrays typically require red, green, and blue color filters to distinguish hues, with difficulty integrating devices featuring different absorption bandgaps onto a single panel, which inevitably causes light absorption losses and reduced responsivity, whereas perovskite PDs can be fabricated using solution processing or chemical vapor deposition techniques whose pixelation process can be completed directly on a single panel allowing full-color detection without color filters. To date, wavelength-sensitive perovskite detectors featuring diverse architectures, including multilayer stacked structures, microcavity configurations, and planar back-to-back arrangements, have been designed to achieve wavelength discrimination and full-color image reconstruction [222]. For example, Liu et al. proposed a filter-free integrated imaging PD system [214]. By employing a set of red (R), green (G), and blue (B) self-filtering narrowband perovskite PDs along with a broadband white PD to mimic the long-, medium-, and short-wavelength cone cells and rod cells of the human visual system, they achieved high-resolution (up to 256 × 256 pixels) single-pixel color imaging in diffusion mode. As schematically illustrated in Fig. 15d, the perovskite PD comprises two spin-coated perovskite layers. One serving as a perovskite filter layer (PFL) with shorter-wavelength absorption, and the other functioning as a perovskite photodetection layer (PPL) with longer-wavelength absorption. Through proper bandgap matching and consequent absorption spectral overlap, the detector generates narrowband photoresponses to red, green, and blue colors, respectively. Building on this architecture, they demonstrated a high-resolution color camera using predesigned patterned illumination and subsequent image reconstruction algorithms. The article presents color imaging capabilities when the RGB PDs are exposed to both adequate light (> 20 μW cm^−2^) and weak illumination (5.4 μW cm^−2^). Results show that employing a simple image overlay method for color/white image fusion maintains robust color imaging capability even under weak lighting conditions (Fig. 15e).

*NIR single-point imaging.* Most reported perovskite PDs based on Pb-based or Bi-based perovskites were only sensitive to the UV–vis spectrum (up to 800 nm). Consequently, fabricating perovskite NIR PDs with high sensitivity requires additional bandgap tuning approaches. The dominant approach focuses on hybrid Pb–Sn halide perovskites, which hold significant potential for advancing NIR detection. The design of Sn–Pb perovskite with mixed composition allowed precise controlling of its light absorption behavior in the NIR region at a direct bandgap of approximately 1.2 eV (corresponding to around 1033 nm) [223]. For example, Ma et al. demonstrated the validity of bandgap tuning via B-site ion doping, successfully extending the detection limit to approximately 1100 nm [182]. Moreover, the researchers demonstrated a prototype day-night detection sensor based on the Pb–Sn alloyed SCs perovskite PDs (Fig. 15f). The designed detector leverages its tunable spectral response characteristics that eliminate the need for mechanical ICR components, significantly reducing the size of day-night surveillance cameras. Furthermore, this perovskite detector can switch between day and night imaging modes simply by adjusting the bias voltage, eliminating dependence on failure-prone mechanical switching devices. For daylight imaging, the authors selected a low-bias mode (-1 V) combined with cost-effective notch filters to block light in the 780 − 900 nm wavelength range. For nighttime imaging, a high-bias mode (-3 V) was employed for monochrome imaging, as its secondary response peak at 940 nm is fully compatible with widely used 940-nm supplemental lighting sources (without red bursts). The authors further demonstrated the color imaging performance of the PD under monochromatic light illumination (red: 650 nm, green: 532 nm, blue: 445 nm) combined with interference light at 940 nm, the schematic diagram shown in Fig. 15g. The results clearly indicate that when using the Si PD, all monochromatic light imaging outcomes exhibited severe color distortion. In contrast, the perovskite PD maintained excellent color reproduction characteristics without noticeable distortion.

II. Array imaging

Array imaging is a point-to-point imaging technique that utilizes multiple sensors to capture image information simultaneously, which is widely employed in mature conventional charge-coupled device (CCD) and complementary CMOS image sensors. This technology provides high resolution, rapid imaging, and intelligent functionality at a relatively low cost, meeting most imaging requirements and applications. A schematic diagram of a typical array imaging system is illustrated in Fig. 15h. Beyond specifying performance requirements for individual detectors, the array imaging technique also demands high uniformity of the arrays. In recent years, innovative developments have been made in the methodologies and applications of array imaging.

*Ultraviolet light array imaging*. Liu et al. advanced an integrated approach combining UV imaging and polarization perception to develop a visual system sensitive to UV circularly polarized light (CPL) [215]. By constructing a heterostructure based on helical one-dimensional chiral perovskites, they demonstrated capabilities in recognizing, learning, and memorizing left-handed CPL (LCP) and right-handed CPL (RCP). Figure 15i illustrates the CPL imaging process inspired by mantis shrimps, which utilize cuticular reflections of CPL for optical communication. Visual signals were encoded into CPL pulses with varying spatiotemporal information to program the developed array. The distinct image contrast observed under LCP versus RCP illumination indicates the significant potential of the fabricated devices for constructing polarization-related neuromorphic visual systems (Fig. 15j).

*Visible spectrum array imaging*. Perovskite-based detector arrays for visible light imaging have been extensively researched. To overcome the limited light utilization efficiency of optical filters in modern image sensors, Tsarev et al. developed an innovative color sensor array architecture by vertically stacking multi-layer lead halide perovskite thin-film PDs (Fig. 15k) [33]. Leveraging the tunable bandgap of perovskites, this design enables selective absorption in the red, green, and blue spectral regions without requiring color filters. To demonstrate the advantages of the designed device, the authors utilized experimental photoresponse data obtained from an 8 × 8 × 3 perovskite sensor array to simulate the image output of both Bayer-pattern sensors and the stacked device. The simulated raw output from the Bayer-pattern sensor exhibited suboptimal image quality, highlighting the negative impact of demos icing on final image resolution. Furthermore, the image displayed noticeable color artifacts. In contrast, the image acquired in Foveon mode demonstrated superior quality without apparent color artifacts, as shown in Fig. 15l, confirming the benefits of the stacked architecture.

*NIR array imaging*. The aforementioned study demonstrates the anti-interference capability of perovskites in single-point imaging. Leveraging their NIR perception capabilities, research has also been conducted on their application in flexible array imaging, particularly in machine vision and biometric identification [224]. For instance, Van Breemen et al. reported a solution-processed high-performance Pb-based photodiode integrated with a high-resolution 508 PPI oxide-based thin-film transistor (TFT) backplane to fabricate an image scanner capable of capturing full-color images and high-resolution fingerprints [216]. To improve the sensitivity of the device for weak light perception, an inverted stacked structure was implemented (Fig. 15m). A key innovation was the introduction of a 50-nm-thick silicon nitride edge cover layer (ECL), which effectively suppressed dark current density. Subsequently, fingerprints were captured under ambient light conditions in transmission mode. Figure 15n presents the fingerprint image after offset/gain correction and bad pixel interpolation, where sweat pores are clearly visible along the ridges, demonstrating the high sensitivity of the fabricated image sensor.

#### Bio-inspired Imaging

Leveraging the exceptional light absorption capacity of perovskite materials, which enables efficient photon capture even in thin-film configurations, a variety of bionic visual perception systems based on curved image sensors have been developed in recent years [225–227]. These imaging devices mimic biological visual systems in both structure and function, not only effectively correcting optical aberrations but also significantly expanding the field of view and enhancing environmental adaptability. Meanwhile, sensor-level visual computing systems inspired by biological synapses and neural networks have been extensively investigated to improve image processing efficiency. By integrating detection, storage, and processing capabilities within individual sensing units, this technology achieves notable reductions in system latency and power consumption. This chapter will systematically elaborate on the recent advances and applications of bio-inspired visual sensors.

**Fig. 16:**
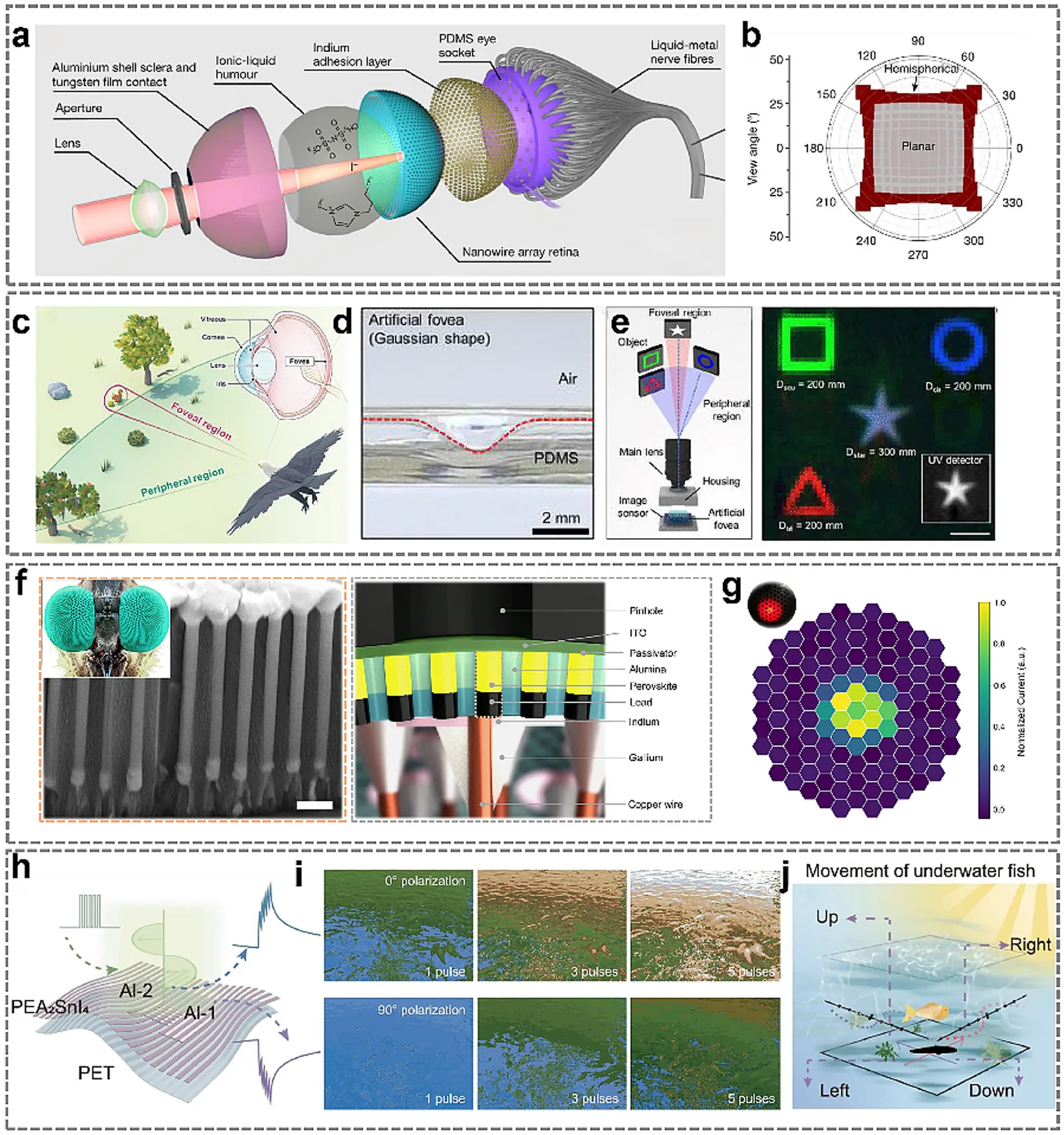
Advanced bio-inspired imaging based on perovskite PDs. **a** Schematic setup of individual pixel measurement. **b** Calculated FOV of the planar and hemispherical image-sensing systems. Reproduced with permission from Ref. [228] Copyright 2020, Springer Nature. **c** Schematic illustration showing the visual ecology of birds. **d** Optical camera image of a cross-sectional view of the artificial fovea. **e** Schematic illustration of the measurement setup for the imaging demonstration. Four objects (triangle, square, circle, and star shape) were placed at two different distances (*D*_tri_ = *D*_squ_ = *D*_cir_ = 200 mm and *D*_star_ = 300 mm). Imaging demonstration of the foveated and multispectral vision. The inset shows the star-shaped image obtained from UV detector. Reproduced with permission from Ref. [229] Copyright 2024, AAAS. **f** Cross-sectional SEM image of perovskite nanowires in hemispherical device and the enlarged view of the designed system. Inset is the macrophotograph of a robber fly’s eye. **g** Captured image of circular pattern. Reproduced with permission from Ref. [230] Copyright 2024, AAAS. **h** Schematic diagram of the device. **i** Simulated polarization imaging landscape map at 0° and 90° polarization angles processed by the imaging system. **j** Schematic trajectory of underwater fish movement in four different states. Reproduced with permission from Ref. [231] Copyright 2025, Springer Nature

The human eye features a quasi-spherical structure capable of perceiving and converging incident light, precisely focusing it onto the retina. The retina then converts these optical signals into neural electrical signals, which are ultimately interpreted by the brain as visual images. This hemispherical configuration effectively simplifies optical design, enabling an ultra-wide field of view of approximately 155°. Inspired by this biological architecture, researchers have developed various bionic human vision systems that employ curved image sensors to mimic the hemispherical structure of the retina. These sensors typically utilize flexible photosensitive materials and stretchable interconnects, allowing stable integration with curved substrates. Taking the electrochemical bionic eye proposed by Gu et al. as an example, its structure is shown in Fig. 16a [228]. The spherical device consists of an optical lens and an aluminum-coated shell serving as the front electrode at the anterior segment, while the posterior section features a hemispherical nanowire array forming the bionic retina. The cavity is filled with an ionic liquid electrolyte. At the rear side of the retina, liquid metal fibers contact the nanowires to form back electrodes, with each contact point defining an individual pixel. The authors further utilized porous PDMS polymer as an artificial orbit and constructed a back electrode array through orderly arrangement of liquid metal fibers, ultimately achieving a complete pixelated architecture. Benefiting from the excellent optoelectronic properties of FAPbI_3_ perovskite, each individual nanowire exhibits sufficiently strong photoresponse. Meanwhile, effective physical isolation of the nanowires by a porous aluminum membrane significantly suppresses inter-pixel crosstalk. Figure 16b demonstrates the imaging result of the letter “A” captured by this bionic eye. Compared to planar image sensors, this hemispherical configuration ensures equidistant distribution between each pixel and the lens, thereby achieving a wider FoV and more uniform pixel-level focusing.

Beyond the human eye, the visual capabilities of various animals have been extensively studied, driving further advancements in bionic electronic eyes. For instance, birds possess vision analogous to a telephoto lens, enabling them to identify prey from great distances in the sky (Fig. 16c) [229]. This visual ability primarily stems from a unique deep foveal structure within their eyeballs. This structure effectively refracts incident light and enhances image details by projecting magnified images of distant objects onto a broader foveal region. Moreover, the retinal cells within the avian fovea are densely packed, preventing resolution degradation caused by image magnification. Inspired by avian vision, Park et al. developed a bionic vision system integrating an artificial foveal structure with vertically stacked perovskite image sensors (Fig. 16d). This system employs a Gaussian-shaped artificial fovea to optically magnify target objects and enhances motion detection by amplifying movement disparities between consecutive frames. To compensate for potential resolution loss induced by image magnification, the image sensor was designed with a non-uniform pixel distribution, achieving the highest pixel density in the foveal region. Furthermore, the constructed artificial vision system demonstrates the capability to detect, track, and identify distant targets in dynamic environments by utilizing foveal imaging to perceive remote objects beyond the focal length (Fig. 16e).

Through prolonged natural selection, insects have evolved unique compound eye structures that enable static and dynamic visual perception in complex environments. Comprising hundreds to thousands of individual ommatidia distributed across a curved surface, compound eyes provide panoramic vision, depth perception, distortion-free imaging, and rapid motion tracking. Inspired by this, Zhou et al. reported a lensless pinhole compound eye system capable of precise target localization and dynamic motion tracking [230]. This visual system utilized perovskite nanowires as photosensitive material to fabricate hemispherical image sensors with fast response times and high photosensitivity. By precisely aligning pinhole arrays as lensless optical channels with perovskite nanowire PDs, potential alignment deviations that may occur during deformation between microlens arrays and detectors were effectively avoided. Furthermore, this compound eye system employed a honeycomb-shaped pinhole array consisting of 121 independent ommatidia, which effectively eliminated visual blind spots between adjacent units and achieved seamless wide-angle imaging within a 140° field of view (Fig. 16f). This design enables simultaneous detection and discrimination of multiple target patterns from various angles. The authors further constructed a binocular vision system comprising two pinhole compound eyes, which was mounted on an unmanned aerial vehicle platform and successfully demonstrated stable tracking of continuously moving light sources (Fig. 16g), verifying the system’s approximately 220° ultra-wide field of view and high-precision target localization capability.

The described bio-inspired vision sensors mimic the wide-field perception of the human eye, the long-range target tracking capability of birds, and the high-precision positioning function of insect compound eyes. Inspired by the parallel processing mechanisms in biological neuromorphic visual systems, researchers have further developed neuromorphic vision sensors. At the core of such sensors are optoelectronic artificial synaptic devices, which simulate synaptic activities between biological neurons and can respond to electrical or optical stimuli. In complex neural networks, synapses serve as the fundamental units for information processing and learning. To date, researchers have developed various types of optoelectronic synaptic devices, including photoelectric and all-optical types. Furthermore, these devices have been extended to achieve multiple bio-inspired visual functions such as visual adaptation, multi-color perception, UV light perception, and polarized light perception, thereby further enriching their application potential in bio-inspired vision systems. In the field of bio-inspired polarization sensing, inspired by the polarization-sensitive vision of kingfishers, the authors have developed a polarization-sensitive optoelectronic synaptic array device based on PEA_2_SnI_4_ microwire arrays (Fig. 16h), enabling multidimensional visual information in-sensor processing in complex lighting environments [231]. Utilizing the memory characteristics dominated by Sn vacancies, the device mimics synaptic functions for primary information processing of the nervous system, including excitatory/inhibitory postsynaptic currents, paired-pulse facilitation, short-term synaptic plasticity, and long-term synaptic plasticity. Owing to the anisotropy of its material morphology and crystal structure, the fabricated device can distinguish polarized light and has successfully identified the number and movement direction of fish beneath glaring water surfaces (Fig. 16i, j). Similar polarization-sensitive properties have also been reported in certain chiral perovskite crystals. Kim et al. realized chiral optical properties in 3D Cs_0.05_FA_0.5_MA_0.45_Pb_0.5_Sn_0.5_I_3_ polycrystalline films by incorporating chiral plasmonic gold nanoparticles (Au NPs) [232]. Circularly polarized light (CPL) detectors fabricated using perovskite films embedded with chiral Au NPs can operate in a self-powered mode, exhibiting significant chirality in the NIR region. CPL PD arrays fabricated on plastic substrates demonstrate highly sensitive, self-powered NIR polarization-sensitive imaging capabilities, combined with excellent flexibility and durability.

### Light Communication

**Fig. 17:**
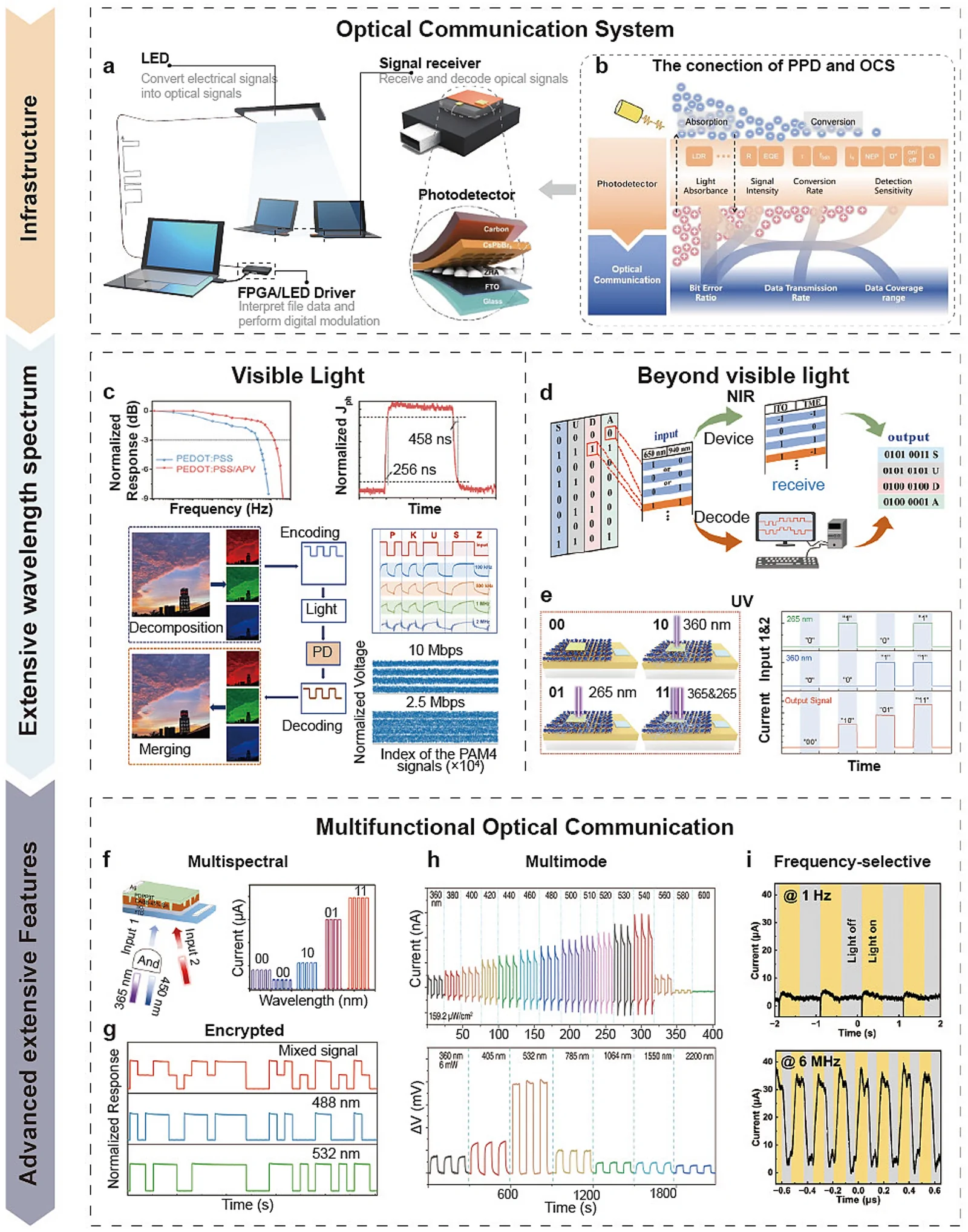
Applications of perovskite PDs in optical communication systems. **a** Schematic diagram of an optical communication system. **b** Connection between the performance of PD and optical communication. Reproduced with permission from Ref. [233] Copyright 2026, Royal Society of Chemistry. **c** Visible light communication system. Reproduced with permission from Ref. [192] Copyright 2025, Wiley. **d** Infrared optical communication encryption solution. Reproduced with permission from Ref. [235] Copyright 2025, Wiley. **e** Schematic diagram of the UV communication system. Reproduced with permission from Ref. [236] Copyright 2025, Wiley. **f** Multispectral communication system. Reproduced with permission from Ref. [237] Copyright 2024, American Chemical Society. **g** Encryption of optical communication signals. Reproduced with permission from Ref. [175] Copyright 2025, Cell Press. **h** Narrowband-broadband dual-mode optical communication. Reproduced with permission from Ref. [238] Copyright 2025, Wiley. **i** Selective frequency optical communication. Reproduced with permission from Ref. [177] Copyright 2024, Springer Nature

Optical communication is a wireless communication technology that employs optical signals as information carriers for transmission over specific distances. It offers advantages including rapid response times, security, low energy consumption, and resistance to electromagnetic interference [233]. PDs are critical components within optical communication systems due to their highly efficient photoelectric signal conversion capabilities, with their response speed crucially determining the overall bandwidth and efficiency of the communication system. A typical optical communication system (OCS) is illustrated in Fig. 17a, where the LEDs at the transmitter port convert electrical signals into optical signals and the PDs at the receiver port convert the optical signals back into electrical signals which are then decoded by the digital-to-analogue converter. Three transmission characteristics are evaluated in optical communication systems: bit error rate (BER), data transmission rate, and coverage range. As a core component of optical communication systems, the performance of PDs can influence key performance indicators that determine the overall functionality of the system, as summarized in Fig. 17b. The intensity of optical signals generated by PDs is a crucial foundation for the reliability of optical communications. Signal intensity is jointly determined by the PD material’s *R* and EQE. Higher signal intensity typically yields a better signal-to-noise ratio (SNR), enhancing noise immunity and thereby reducing the bit error rate (BER) in optical data transmission. The linear dynamic range, spectral response range, and spectral resolution of PDs are the three primary factors governing the coverage range of optical communication systems. The signal conversion rate directly determines the maximum optical signal modulation frequency that the PD can track, representing a critical bottleneck limiting the data transmission rate of the entire optical communication system. This rate is primarily governed by the response time (*τ*_rise_/*τ*_fall_) and bandwidth (*f*_3dB_) [234]. Consequently, the PD performances define the theoretical upper limit for maximum transmission rates in optical communication systems, serving as a fundamental prerequisite for achieving high-speed data transmission. Specifically, high-bandwidth communication places stricter demands on the response time of perovskite PDs. To meet these requirements, necessary performance optimization strategies become crucial. For example, to shorten the charge transport path, the absorption layer must be made extremely thin, while the resulting loss in light absorption can be mitigated through optical management engineering; simultaneously, the design of the interfacial band structure ensures the rapid extraction of transient photogenerated charges.

#### Various-Wavelength Optical Communication

The earliest optical communication applications were primarily concentrated in the visible light spectrum (300 − 900 nm). The workflow of visible light communication (VLC) systems for image information is detailed with the aid of the work by Chen et al. Chen et al. introduced a novel violanone derivative, 1-allyl-10-(2-phosphonethyl) violanone (APV), to enhance the crystalline quality of perovskite films and effectively suppress dark current [192]. The APV-modified perovskite PDs was applied in back-to-back OWC systems, as shown in Fig. 17c. The process began by decomposing the color image into three color channels (red, green, and blue). These channels employed on–off keyed modulation to control the intensity of the 680 nm LED at a frequency of 1 MHz. The modulated light signal transmitted through free space was then detected by APV-enhanced perovskite PDs and captured via an oscilloscope. Following detection, the received signal was decoded, and the color channels recombined to reconstruct the color image. The device successfully transmitted images error-free at 1 MHz, highlighting its significant bandwidth. Furthermore, the system achieved a BER of 0% at 10 Mbps and 0.018% at 12.5 Mbps.

To meet diverse application requirements, the researchers have extended optical communication into the UV and NIR wavebands [239]. Li’s group reported a self-powered perovskite/silicon hybrid visible-to-NIR dual-response PD, where the dual response is tuned by controlling the transparency of the transparent electrode and the interfacial contact. In their constructed optical communication system, the opposite-polarity optical response induced by NIR light served as a complementary signal to the visible light signal, as shown in Fig. 17d. Consequently, the receiver needed to distinguish between positive and negative signals transmitted by the device, without differentiating signal amplitudes caused by variations in light intensity. This approach avoided the inaccuracies inherent in traditional dual-channel optical communication encryption systems, which rely on adjusting light intensity to differentiate signal response values.

Compared to VLC, UV communication is highly attractive due to its low background solar radiation, particularly in the UVC band (100 − 280 nm) that is strongly absorbed by the ozone layer. Low-noise free space communication in the UVC spectrum supports various applications, such as missile detection and aircraft landing under low-visibility conditions [240]. Ma et al. designed and fabricated a self-powered dual-band UV PD based on a Cs_3_BiCl_6_/GaN heterojunction using dual-source coevaporation technology [236]. Experimental and theoretical simulations confirmed that the unique dual-band absorption characteristics of Cs_3_BiCl_6_ and the strong surface charge recombination near its surface are the primary factors enabling UV dual-band photodetection. The unique dual-band UV photoresponse characteristics of the Cs₃BiCl_6_/GaN heterojunction PD have endowed this device with tremendous potential for applications in encrypted optical communications. Benefiting from the availability of this device to respond to daylight-blind light (265 nm) signals, it served as a supplementary optical communication signal in UVB waveband (Fig. 17e). The inherent difficulty in capturing daylight-blind light reduced the feasibility of conventional PDs for signal decryption, thereby ensuring the security of information transmission.

#### Multifunction Optical Communication

Optical communication serves as a vital complement to wireless communication, providing a crucial supplement to traditional radio frequency technology amid today's scarcity of wireless spectrum resources. As optical communication systems advance, single-function optical systems can no longer meet the demands for efficient, lossless, and secure information transmission. Consequently, diverse functionalities, including multispectral integration, encryption, multi-mode switching, and frequency selection, are incorporated into optical communication systems to address key challenges in modern wireless communication [95, 233].

As described in the previous section, perovskite PDs possess the ability to achieve UV to NIR detection by tuning their bandgap, enabling the realization of multi-channel optical communication systems. Developing an integrated PD capable of operating at room temperature with a light response range extending from UV to NIR could significantly reduce the complexity and cost of receiving systems in multispectral applications. Mao et al. leveraged the porous structure of Cs_2_AgBiBr_6_:Sn thin films to construct an efficient Cs_2_AgBiBr_6_:Sn/PDPP3T heterojunction, thereby realizing a self-powered detector with broad spectral response (UV–Vis-NIR) [237]. This perovskite PDS exhibited a broad spectral response from 350 to 950 nm, with an average detection rate exceeding 10^11^ Jones, and maintained excellent photoconductive performance for two months. Leveraging its wide spectral response across the UV–Vis-NIR range, a novel information transmission system employing all-optical modulation was realized, integrated within a single perovskite PD, as shown in Fig. 17f. The left pattern illustrated the operating principle of the PD: 365 nm and 450 nm lasers are coupled through optical fibers to function as an AND gate, while the 808-nm laser illuminates independently. And it was displayed that the output photocurrent of the perovskite PD exhibited excellent linear superposition under both monochromatic and multi-color illumination conditions in the right pattern. Consequently, it could effectively enhance optical communication transmission efficiency by superimposing optical information. Furthermore, multispectral communication also lays the foundation for encrypted communication, enhancing the security of information transmission. The working principle of signal encryption is illustrated in Fig. 17g. Specifically, the 26 English letters corresponding to Arabic numerals 1 − 26 are first converted into decimal numbers and then transformed into binary data to generate the ASCII code for each letter. To facilitate decryption at the receiving end, each letter’s ASCII code corresponds to four bits of binary data. Two distinct information streams carrying pseudo-information can be generated using 488 and 532-nm light beams. Subsequently, the mixed signal is transmitted to the receiver. Using the codebook and a known key (applying bias and light intensity), two independent information streams can be separated from the mixed signal. The true information is then further decoded through alphabet conversion [175].

In traditional optical communication systems, PDs typically support either broadband or narrowband detection. Broadband PDs are relatively easy to manufacture due to the broad optical absorption characteristics of photoreactive materials. In contrast, narrowband PDs are limited to specific spectral ranges. However, single-mode PDs are unsuitable for efficient optical communication encryption because they require separate devices for different signal bands. Developing a dual-mode photodetection method capable of simultaneously processing both broadband and narrowband signals is essential. Hu et al. demonstrated self-powered dual-mode perovskite PDs integrating the interfacial photothermal effect (IPPE) and parallel photothermal-electric effect (PTEE) through Ag⁺ doping and integrated broadband absorbers, enabling dual-mode switching between broadband and narrowband detection [238]. High-performance narrowband detection derived from IPPE and augmented by vertical charge collection narrowing effect enabled specific wavelengths to penetrate interfaces with enhanced carrier separation efficiency and elevated temperatures. In lateral perovskite PDs, broadband metamaterial absorbers serving as counter electrodes improved photothermal conversion and broadened light absorption, achieving ultra-wideband response spanning 360 to 2200 nm. By altering the halide type in perovskite SCs, the specific response band of the narrowband perovskite PDs could be modulated from violet to red light while maintaining broad spectral responsivity. Dual-mode photodetection was fully leveraged to achieve dual encryption during signal transmission (Fig. 17h).

Not all optical communication applications possess sealed optical transmission channels. As a crucial aspect of optical communications, free space coupling plays an indispensable role in air-to-ground information exchange, underwater wireless optical communications, and distributed optical fidelity communications [241]. Frequency-selective optical response appears to offer significant advantages in signal extraction, demonstrating potential for free space coupled optical communication systems application. Min et al. have demonstrated an asymmetric 2D-3D-2D perovskite PD comprising two photodiodes with back-to-back structure, enabling frequency-selective optical response in a single device [177]. The device exhibited no current generation at the low frequency of 1 Hz, yet produced significant photocurrents with at frequencies up to 6 MHz per unit cell in the perovskite device. Furthermore, the 3 dB cut-off frequency was calculated to lie within the range of 0.8–9.7 MHz, simultaneously meeting the frequency range and frequency selectivity requirements for free space optical communication, as shown in Fig. 17i. They demonstrated optically driven signals during character and video transmission in indoor lighting conditions with intense optical interference, proving the robust anti-interference transmission capability of this frequency-selective perovskite PDs.

### Wearable Electronics

**Fig. 18:**
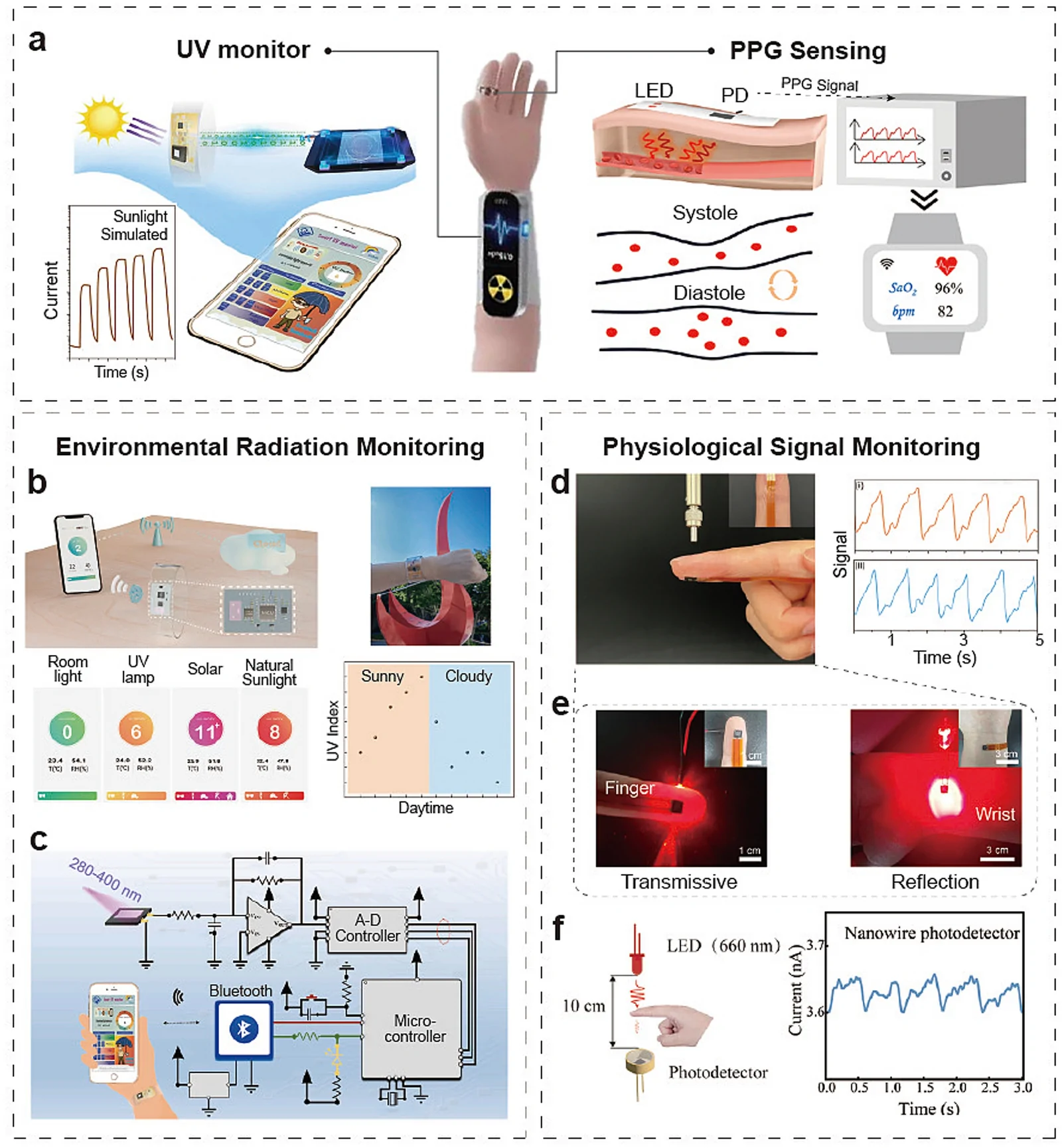
Applications of perovskite PDs in wearable electronics. **a** Applications of perovskite PDs in wearable devices include environmental UV radiation monitoring and physiological signal monitoring. Reproduced with permission from Ref. [242] Copyright 2025, Springer Nature. Reproduced with permission from Ref. [171] Copyright 2024, Elsevier. Reproduced with permission from Ref. [243] Copyright 2023, Cell Press. Reproduced with permission from Ref. [105] Copyright 2025, Wiley. **b** Perovskite PD-based wearable UV monitoring wristband system. Reproduced with permission from Ref. [244] Copyright 2022, Elsevier. **c** Circuit block diagram of the portable UV radiation monitoring system. Reproduced with permission from Ref. [171] Copyright 2024, Elsevier. **d** Application of perovskite PDs in blood pulse signal detection. Reproduced with permission from Ref. [245] Copyright 2023, Springer Nature. **e** High-fidelity pulse signals obtained by the PPG sensor operating in transmission and reflection modes. Reproduced with permission from Ref. [168] Copyright 2024, Springer Nature. **f** Contactless heart rate monitoring using MAPbI₃-based perovskite PDs. Reproduced with permission from Ref. [246] Copyright 2025, Wiley

Non-invasive technology employing high-performance PDs to monitor physiological signals such as heart rate, blood oxygen saturation, and respiratory metrics has been widely adopted in wearable devices due to its excellent extensibility, conformability, and biocompatibility. However, traditional PDs face significant limitations in wearable flexible electronics. Their inherent rigidity leads to mechanical failure under repeated bending, their intrinsic band structure restricts multispectral sensing capabilities, and bulky packaging hinders seamless integration with deformable substrates. Compared to conventional silicon-based information devices, perovskite PDs exhibit inherent advantages such as low-temperature solution fabrication processes and bandgap tunability through over-composition engineering, offering promising solutions to critical limitations of traditional semiconductors in wearable applications [227]. Low-temperature solution processes (< 150 °C) enable direct sensor integration onto flexible substrates like PET and PI. Broadband sensitivity spanning UV–visible to NIR supports multi-scenario sensing (e.g., simultaneous monitoring of UV exposure and PPG). For perovskite PDs in flexible wearable electronics, the key considerations are biocompatibility and stability. Under frequent dynamic stress from stretching and bending during contact with the human body, interface delamination and microcrack propagation between the perovskite and the flexible substrate caused by differences in thermal expansion coefficients and Young’s moduli directly determine the actual service life of the device in wearable applications. To address these requirements, high-quality polycrystalline films with low defect density can provide excellent intrinsic mechanical flexibility and resistance to stress-induced delamination. Additionally, the passivating effect of long-chain molecular additives can reduce surface defects while blocking the ingress of water and oxygen and providing certain self-healing capabilities. These strategies collectively support the application of perovskite PDs in wearable electronics. Figure 18a summarizes perovskite PDS-based wearable devices for health monitoring.

#### Environmental UV Radiation Monitor

As a significant component of solar radiation, excessive UV exposure has been proven to cause sunburn, skin aging, and skin cancer (the most common type of cancer globally). The UV wavelength range spans 100 − 400 nm and categorizes into three types: UVA, UVB, and UVC, corresponding to wavelength intervals of 315 − 400, 280 − 315, and 100 − 280 nm, respectively. Nearly all UVC radiation and approximately 90% of UVB radiation are absorbed by the atmosphere. Consequently, there is an urgent need for wearable UV sensors with real-time, precise detection capabilities—capable of identifying radiation combinations dominated by UVA with minimal UVB—to mitigate the potential risks associated with prolonged UV exposure [247].

The exceptional tunable bandgap and customizable spectral selectivity of perovskites enable their design as PDs suitable for monitoring UV radiation. As a member of the lead chloride perovskite family, MAPbCl_3_ exhibits a broader bandgap compared to FAPbCl_3_ and CsPbCl_3_, making it particularly suitable for UV sensing applications. Zhou et al. designed a wearable UV monitor using a flexible perovskite PDS with a vertical PET/ITO/SnO_2_/MAPbCl_3_/PTAA/Au structure [244]. The MAPbCl_3_ layer exhibiting high UV absorption was prepared via a solution-assisted halide exchange method. Notably, the flexible UV-perovskite PDs retained over 81% of its initial optical response after 2500 bending cycles at a radius of 7 mm. Moreover, owing to the high crystallinity and film quality of MAPbCl_3_, the encapsulated UV-perovskite PDs exhibited stable current output for 500 h under continuous UV illumination (395 nm). Furthermore, by integrating the device with a flexible printed circuit board, a wearable UV monitoring wristband was developed. This wristband was capable of real-time monitoring of UV radiation under various conditions, including indoor lighting, UV lamps, solar simulators, and natural sunlight as shown in Fig. 18b. The specific circuit design for this type of UV monitoring system is shown in Fig. 18c [171]. Specifically, the dual-ended output of the self-powered perovskite PD is first connected to the input of a transimpedance amplifier (TIA) module to convert the photocurrent into a voltage signal. This converted analog voltage signal is then normalized into a digital signal via an analog-to-digital converter (ADC), which can be processed by a microcontroller (MCU). The normalized digital signal is transmitted via the commercial Bluetooth 4.0 communication protocol to a proprietary Android application. Such a system with a low-cost fabrication and potable characteristics will exactly be a general platform for UV monitor applications.

#### Physiological Signal Monitoring

Photoplethysmography (PPG) represents the most prominent non-invasive blood oxygen monitoring technique. This method characterizes critical health parameters, such as blood pressure, oxygen saturation, and heart rate, by analyzing the absorption rates of human vascular tissue at different wavelengths, offering potential for flexible PD applications in assistive healthcare. Specifically, when a beam of light is projected onto the skin surface at the fingertip, the beam transmits through the tissue into the PD, while part of the light is absorbed, reflected, and scattered by the human tissue. During the test, the light intensity detected by the PD fluctuates in response to changes in blood vessel volume caused by the heartbeat. When the heart contracts, the increased blood volume in the vessels decreases, reducing the light intensity detectable by the PD (absorbed by blood). Conversely, in the diastolic phase, more light can transmit to the PD [248]. Thus, physiological data such as heart rate and blood volume can be extracted from the photocurrent signal to assess human cardiopulmonary function, as shown in Fig. 18d [245].

Taking an innovative wearable remote health monitoring system developed by Liu et al. as an example, the system comprised a wearable health monitoring module and a remote optical communication module utilizing Sn–Pb perovskite-based PDs [168]. The device effectively detected NIR light capable of penetrating skin, enabling the device to operate in both transmission and reflection modes (Fig. 18e). Within this system, 850-nm light emitted by a red LED was absorbed, reflected, and scattered by human tissue, which allowed the perovskite PDs worn by the first user to detect blood volume changes with each heartbeat. Concurrently, a remote optical communication module was constructed based on this PDs. In the remote optical communication module, the detected pulse wave modulation acted as the on/off state of the blue LED in the transmitter, which was received by another device worn by the second user and then transmitted to a computer for analysis. The device demonstrated high sensitivity and detection rates, successfully measuring resting heart rates of 67 bpm and post-exercise rates of 115 bpm. Using two modes of PPG sensing, transmission mode and reflection mode, the reliability of PPG devices has been validated by measuring blood pulse signals in both resting and active states of the human body. This research highlights the significant potential of perovskite PDs for wearable health monitoring and real-time data transmission.

Devices attached to the human body for physiological signal monitoring must consider the potential harm of their toxicity to the body, yet the toxicity of perovskites contradicts this requirement. While developing lead-free perovskite materials is one approach, non-contact monitoring may offer a superior solution in comparison. However, non-contact measurement methods often cause attenuation of the pulse wave signal, challenging the monitoring process and placing higher demands on the detection performance of perovskite PDs. Shen’s group proposed a strategy to optimize MAPbI_3_ nanowire PDs through surface state engineering using C_60_ interface layer. The interface layer formed an electron-depleted region within the nanowires, reducing noise current by 74% and tripling the photoresponse [246]. Consequently, a remarkable D* of 6.7 × 10^14^ Jones was realized, the highest value reported for MAPbI_3_ nanowire-based PDs to date. The high-performance PD facilitated non-contact heart rate monitoring. Compared to silicon PDs, the nanowire device clearly captured periodic pulse wave signals despite minor noise (attributed to slight finger tremors during measurement), as shown in Fig. 18f, demonstrating superior performance and reliable pulse detection capability beyond commercial silicon PDs.

### X-Ray Detection

X-ray detection plays a vital role in medical imaging, industrial defect detection, and scientific research. However, as a form of high-energy radiation, the precise detection of X-rays presents significant challenges. Current commercial X-ray detectors based on silicon, amorphous selenium (a-Se), cadmium telluride (CdTe), and cadmium zinc telluride (CZT) are limited by their low atomic absorption coefficients, rendering them ineffective at blocking X-rays. Moreover, with the increasing demand for X-ray imaging applications, achieving lower radiation doses and higher-resolution X-ray imaging has become the primary objective for next-generation X-ray detectors. However, finding an excellent semiconductor that meets all prerequisites for X-ray detection and can be easily integrated with the readout electronics of planar X-ray detectors remains a highly challenging task [249]. As an improvement pathway, halide perovskites have attracted significant attention in X-ray detection [250]. The key requirements for high-energy X-ray detection are extremely high photon blocking efficiency and charge collection at large thicknesses. The development of perovskite PDs in this field has been driven by heavy-atom substitution (such as Bi-based modification) and advances in dry-process thick-film growth and single-crystal fabrication techniques. Meanwhile, the growth of ultra-thin and high-quality single crystals using methods such as confinement techniques has opened up the potential for large-area imaging and flexible applications in the X-ray detection field.

#### Performance Improvement for Low Radiation Dosimetry Detection

**Fig. 19:**
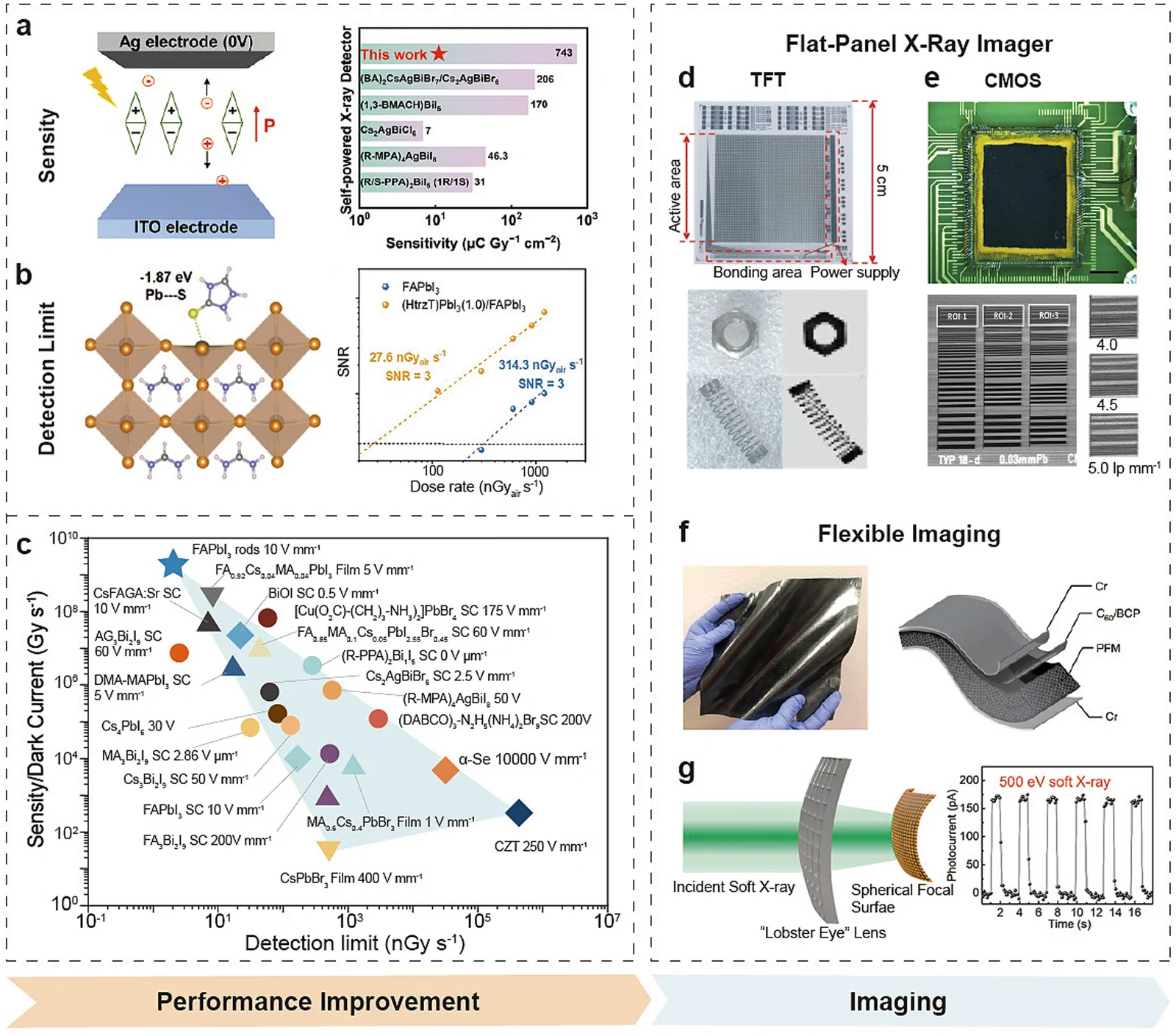
Applications of perovskite PDs in X-ray detection. **a** Dipole arrangement and carrier transport of the (Br-PA)_3_BiI_3_Br_3_-based device under X-ray irradiation and its sensitivity improvement. Reproduced with permission from Ref. [251] Copyright 2025, Wiley. **b** Diagrams of calculated binding free energy of FAPbI_3_ and HtrzT^+^ and the detection limit improvement in (HtrzT)PbI_3_-based detector. Reproduced with permission from Ref. [206] Copyright 2025, Springer Nature. **c** Summary of the sensitivity and detection limits for advanced X-ray detectors in recent researches. Reproduced with permission from Ref. [204] Copyright 2025, Wiley. **d** TFT-based X-ray imaging system. Reproduced with permission from Ref. [252] Copyright 2023, Wiley. **e** CMOS-based X-ray imaging system. Reproduced with permission from Ref. [253] Copyright 2024, Springer Nature. **f** Perovskite microcrystalline films for flexible X-ray imaging. Reproduced with permission from Ref. [254] Copyright 2020, Springer Nature. **g** Schematic of a curved X-ray imaging sensor. Reproduced with permission from Ref. [255] Copyright 2024, Wiley

Sensitivity, as the responsiveness of a detector to incident X-rays, serves as the core metric quantifying the signal conversion capability of a detector. Detection limit characterizes the minimum radiation dose threshold at which the detector can reliably distinguish true signals from background noise interference. Consequently, in recent years, researchers have devoted substantial efforts toward enhancing the sensitivity of X-ray detectors and lowering their detection limits [256]. Following this line of thought, Wang et al. developed a lead-free ferroelectric perovskite material (Br-PA)_3_BiI_3_Br_3_ with a high saturation polarization intensity of 0.39 µC cm^−2^ [251]. The spontaneous ferroelectric polarization promoted charge separation and transport, thereby reducing the exciton binding energy (112.8 meV) and extending the charge carrier lifetime (0.96 ms), ultimately achieving a remarkable mobility-lifetime product (1.08 × 10^−3^ cm^2^ V^−1^). This spontaneous ferroelectric polarization yielded a bulk photovoltaic effect, enabling the device to operate at 0.2 V open-circuit voltage for self-powered operation. The (Br-PA)_3_BiI_3_Br_3_-based X-ray detector achieved a sensitivity of 743 µC Gyair^−1^ cm^−2^ at 0 V bias, representing the highest value reported for lead-free, self-powered perovskite detectors, as shown in Fig. 19a. Furthermore, its inherent 0D structure significantly elevated the activation energy for ion migration (808.9 meV), thereby achieving a low current drift of 7.63 × 10^−17^ A cm^−1^ V^−1^ s^−1^ and markedly enhanced operational stability. After 30 days of exposure under ambient conditions, the device retained 96.3% and 99.7% of its initial photocurrent at 0 V and 10 V bias, respectively. In addition, Huang et al. utilized a conjugated organic cation (1H-1,2,4-triazole-3-thiol, HtrzT⁺) at grain boundaries of FAPbI_3_ perovskite, forming trace amounts of LD(HtrzT)PbI_3_ perovskite, which exhibited a conjugated organic cation and exhibited excellent matching with the α-FAPbI_3_ lattice in the two-dimensional plane [206]. Benefiting from the lattice matching between (HtrzT)PbI_3_ and α-FAPbI_3_, the anchoring lattice enhanced the Pb-I bond strength and effectively alleviated the inherent tensile strain in the α-FAPbI_3_ crystal lattice. The X-ray detector based on (HtrzT)PbI_3_-perovskite device achieved a low detection limit of 27.6 nGy·air s^−1^, attributed to residual stress release and improved charge carrier mobility-lifetime product, as shown in Fig. 19b. Furthermore, the detector displayed exceptional stability under X-ray irradiation, withstanding radiation doses equivalent to nearly 1.17 × 10^6^ chest imaging exposures.

Figure 19c summarizes the currently advanced X-ray detectors performance, including detection limits and sensitivity/dark current density ratio (S/J, a more reliable quality factor for evaluating X-ray detectors) [204]. It can be observed that perovskite-based X-ray detectors significantly outperform commercially available CZT detectors, with continuous breakthroughs in low-dose detection. The lowest detection limit falls below 10 nGy_air_ s^−1^, while the highest S/J exceeds 10^9^ Gy_air_ s^−1^.

#### X-Ray Imaging

X-ray imaging, a technique that utilizes X-rays passing through a planar X-ray detector to reveal the internal structure of materials relies not only on the performance of the integrated X-ray detector but also on the external integrated system for its imaging quality. For example, Wu et al. reported an FA_0.9_MA_0.05_Cs_0.05_Pb(I_0.9_Br_0.1_)_3_-based (FAMACs) X-ray detector, demonstrating a high spatial resolution of 0.46 lp/pixel and excellent thermal stability under industrial standards after integrated with a thin-film transistor (TFT) backplane, as shown in Fig. 19d [252]. The spatial resolution of X-ray imaging was evaluated by measuring MTF using the oblique edge method. The FAMACs-based X-ray imaging achieved a spatial resolution of 0.46 line pairs per pixel at MTF = 0.2. Further, the imaging capability of the X-ray detector was demonstrated by visualizing a nut and spring, with the object contours clearly discernible. The TFT array measured 5× 5 cm^2^, with an effective area of 3.2 × 3.2 cm^2^ and an imaging resolution of 64 × 64 (pixel pitch: 500 μm). Although most previously reported X-ray imaging systems rely on TFT substrates, CMOS based on single-crystal silicon offers superior electron mobility and smaller pixels, presenting a promising alternative for high-performance planar X-ray detectors [250]. Compared to TFT technology, it enables faster readout speeds and higher spatial resolution. Liu et al. reported a dynamically direct-conversion CMOS-compatible X-ray detector assembled using screen-printed CsPbBr_3_ material, exhibiting a mobility-lifetime product of 5.2 × 10^−4^ cm^2^ V^−1^ and a sensitivity of 1.6 × 10^4^ µC Gy_air_^−1^ cm^−2^. The layer-by-layer printed CsPbBr_3_ perovskite was integrated onto a CMOS array (72 × 72; pixel size: 83.2 µm) for X-ray imaging, as shown in Fig. 19e. By scanning the detector at 300 frames per second in both vertical and horizontal directions, rapid imaging of samples larger than 5 × 10 cm^2^ was realized [253]. Compared to conventional indirect conversion CMOS X-ray detectors, this perovskite CMOS detector provided high spatial resolution (5.0 lp mm^−1^ X-ray radiographic imaging capability at low radiation doses (260 nGy). Furthermore, 3D tomographic images of biological samples were successfully reconstructed.

Furthermore, compared to traditional rigid detectors, flexible detectors can bend to conform to non-planar objects or perform imaging in confined spaces without requiring external irradiation. Huang’s group reported a perovskite-filled membrane (PFM) structure for high-sensitivity, flexible, and large-area X-ray detectors [254]. By saturating a porous polymer membrane with a perovskite solution followed by thermal lamination, PFMs with an area up to 400 cm^2^ were formed. The excellent connectivity and crystallinity of perovskite crystals within the film enabled a high mobility–lifetime product. Under an electric field of 0.05 V µm^−1^, the X-ray detector achieves a sensitivity of 8,696 ± 228 µC Gy_air_^−1^ cm^−2^. No performance degradation was observed after over six months of storage or exposure to a dose of 376.8 Gy_air_ (equivalent to 1.88 million chest X-ray scans). Most importantly, the flexible PFMs maintained performance even at bending radii as low as 2 mm. An independent detector arrays, curved and housed within a metal tube, demonstrated superior imaging quality compared to flat-panel detectors, as shown in Fig. 19f. Tan et al. employed a nanopermeable network electrode structure to effectively reduce light loss caused by insensitive layer shading [255]. Simultaneously, they constructed a multidimensional perovskite heterojunction structure to further minimize photogenerated carrier loss, achieving high-performance soft X-ray image sensors. Subsequently, a curved soft X-ray imaging array was fabricated, demonstrating stable soft X-ray response and sharp imaging capabilities, as illustrated in Fig. 19g. It highlights the strong candidate advantage of low-cost, high-efficiency perovskite PDs as next-generation soft X-ray image sensors.

**Fig. 20:**
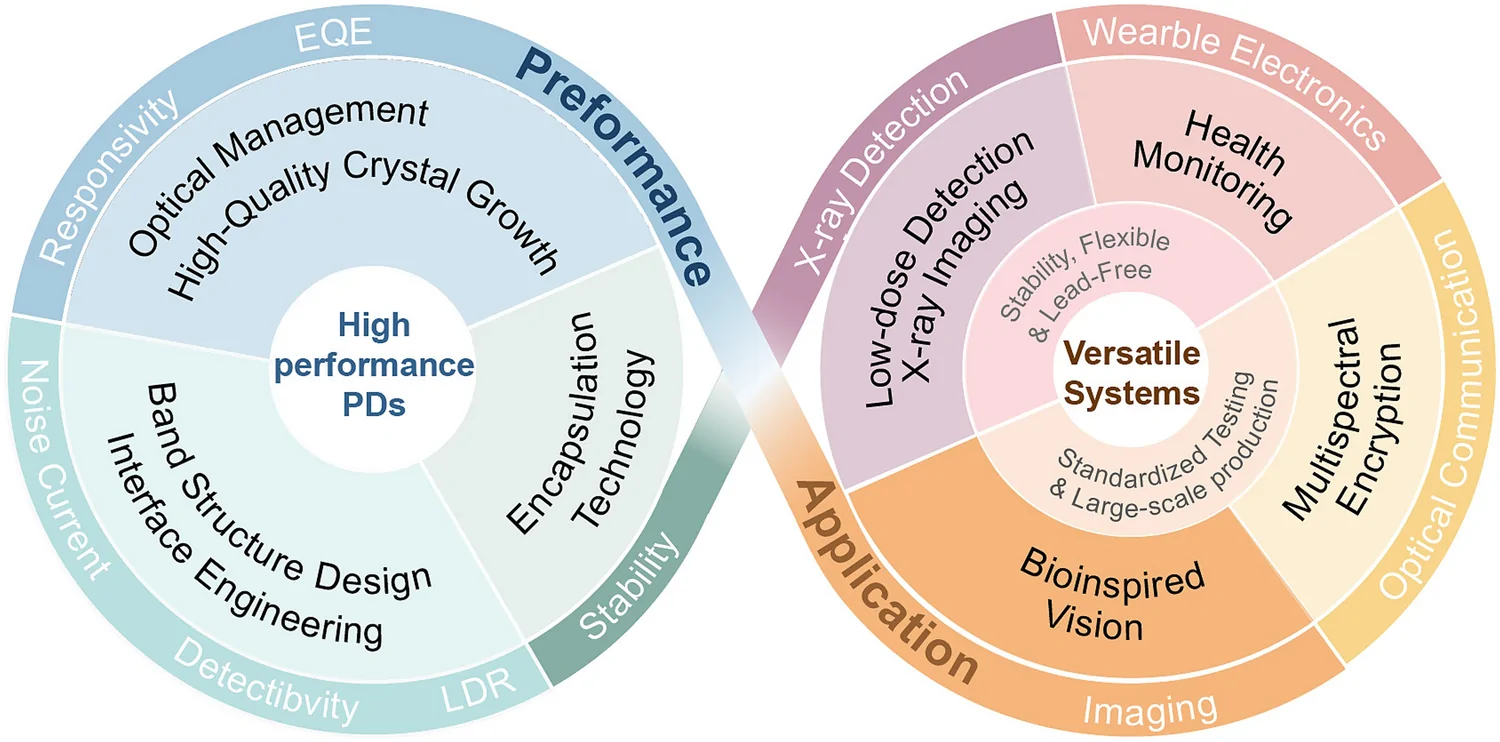
Relationship between the performance parameters, optimization strategies, and applications of perovskite PDs

Benefiting from synergistic optimization of material preparation and device architecture, high-performance perovskite PDs have demonstrated transformative application potential in numerous cutting-edge interdisciplinary fields, as shown in Fig. 20. The unique spectral tunability of perovskites has driven the evolution of high-resolution and multimodal fusion imaging arrays, while also extending traditional imaging into the realms of bionic and neuromorphic vision systems. Furthermore, with their ultra-fast response speeds and inherent mechanical flexibility, they offer significant advantages for applications in optical communications and wearable conformal electronics. Beyond this, the intrinsic heavy-atom effect of perovskites has successfully extended the detection spectrum of devices into the high-energy radiation range, enabling X-ray detection. Future perovskite PDs will undergo material and device codesign tailored to specific spectral ranges and operational scenarios, driving the practical implementation of next-generation intelligent detection systems.

## Challenges and Perspectives

**Fig. 21:**
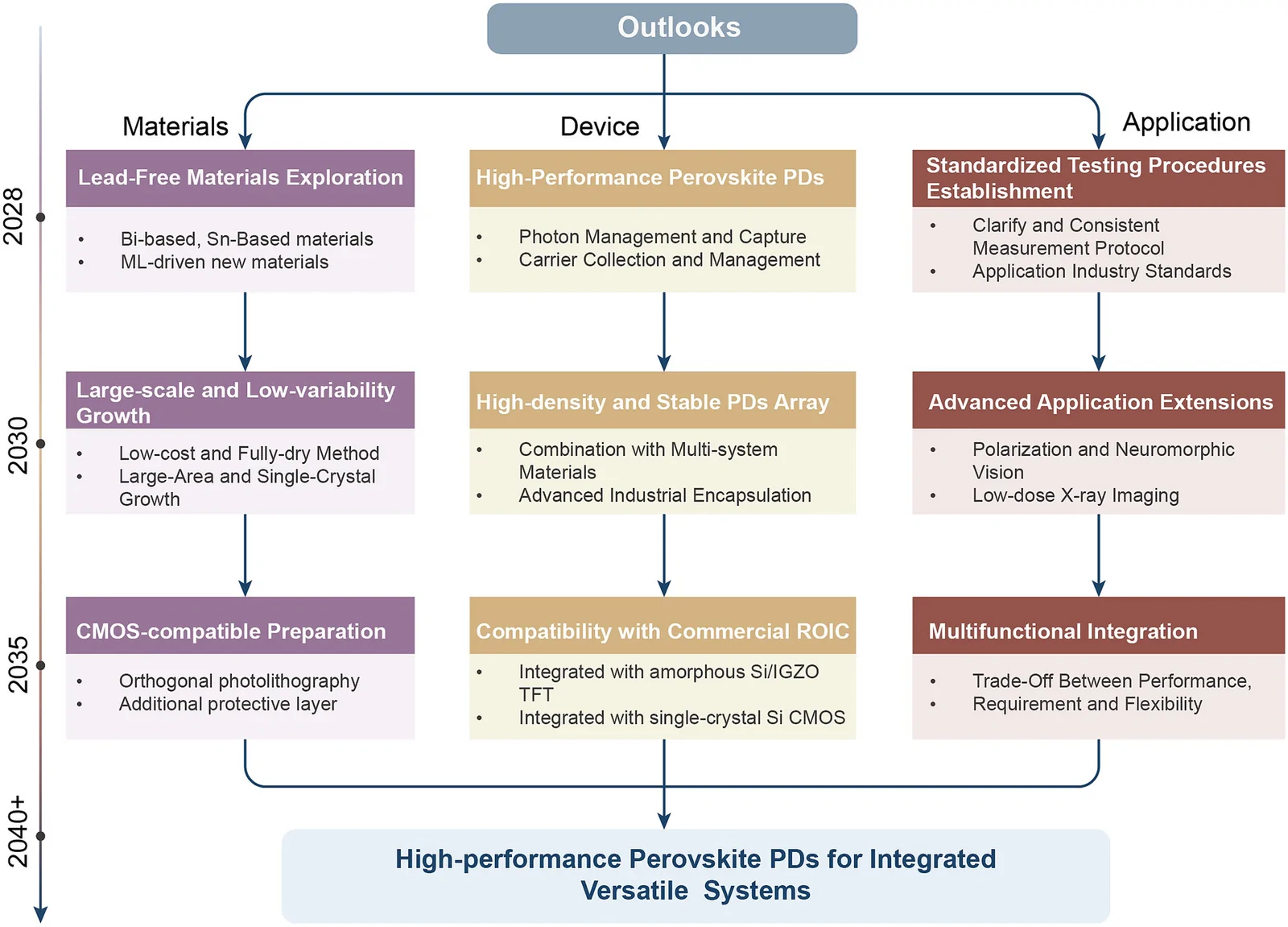
Overview of challenges and countermeasures for perovskite PDs

In this review, we demonstrate that the significant progress has been made in enhancing the performance of perovskite PDs, enabling emerging applications [257]. However, challenges related to toxicity, stability, large-area fabrication, and standardized commercial testing remain to be addressed before practical and widespread implementation should be achieved, as summarized in Fig. 21 regarding future challenges and roadmap.

### Lead-free Perovskite PDs

Although lead-containing perovskites demonstrate outstanding performance in PD applications, the presence of lead restricts their widespread adoption in industrial-scale production and commercial wearable devices. A commercial trade-off exists between non-toxicity and high performance, making the substitution of lead with non-toxic elements essential. By replacing Pb^2+^ with Cu^+^, Sn^2+^, Bi^3+^, Sb^3+^, or Ag^+^Bi^3+^, the lead-free perovskite materials can be prepared and exhibit superior photodetection performance compared to Pb-based perovskite materials under specific conditions. Sn-based perovskites appear more promising, primarily due to their narrow bandgap, high carrier mobility, and high absorption coefficient, which exhibit response from UV to NIR, filling the gap in the NIR region left by lead-based PDs. Bi^3+^ occupies an adjacent position in the periodic table, possessing an ionic radius similar to Pb^2+^. It can be incorporated into the perovskite lattice while exhibiting carrier mobility comparable to that of lead-based perovskites. Moreover, compared to unstable cations such as Sn^2+^ and Ge^2+^ in atmospheric conditions, Bi^3+^ exhibits greater stability toward humidity, demonstrating the potential for storage in air for 2300 h with negligible responsivity degradation. Compared to Sn- and Bi-based photodiodes, research on Sb-, Cu- and Yb-based perovskites PDs appears less extensive, yet these devices exhibit equally competitive characteristics. For instance, MA_3_Sb_2_I_9_-based photodiodes exhibited rapid response speeds and high gain due to their low trap density and long carrier diffusion length. Cs_3_Cu_2_I_5_-based perovskites PDs have realized narrow response bandwidths in the UV region, capable of operating at elevated temperatures with microsecond-level response speeds.

However, the practical application of lead-free perovskite PDs faces distinct challenges. For Bi-based, Sb-based, Cu-based, and double perovskite materials, their indirect bandgap characteristics result in relatively low optical absorption coefficients, consequently yielding lower corresponding sensitivities [2]. With the tendency of Sn^2+^ to oxidize readily and crystallize rapidly, it leads to low-quality films that significantly compromise device stability and performance, despite various efforts, such as synthesizing materials in diverse morphologies like nanowires and SCs, employing additive engineering to introduce reducing agents or chelating molecules to inhibit Sn^2+^ oxidation [149], or preparing stable vacancy-ordered perovskites (Cs_2_SnX_6_) with high visible light absorption coefficients to achieve improved stability [258]. The design and prediction of a large number of new perovskite materials assisted by machine learning (ML) could represent a promising pathway. In recent studies, an ML algorithm aimed at minimizing decomposition was trained using a database derived from high-throughput DFT calculations [259]. This approach predicted the properties of an additional 14,190 perovskites and identified 2,275 more stable perovskite compounds. These findings provide valuable insights and inspiration for the future design of lead-free, stable perovskite PDs.

It must be objectively acknowledged that current assessments of the practical potential of lead-free Sn-based and Bi-based perovskite PDs remain overly optimistic. At present, these lead-free alternatives are still unable to replace traditional lead-based perovskite materials, whether in the more mature photovoltaic sector or the emerging field of high-performance PDs. The spontaneous oxidation of Sn-based perovskites and the strong carrier–phonon coupling in Bi-based materials both stem from their intrinsic physical structural characteristics. Relying solely on device engineering is unlikely to bridge the performance gap. Hence, lead-free perovskite PDs cannot simultaneously achieve both environmental sustainability and optimal optoelectronic performance in the short term, and their commercialization process remains in the extremely early stage of fundamental material validation [5, 68]. Yet, following a similar development trajectory to that of Pb-based perovskites in terms of performance optimization and stability enhancement, it is reasonable to believe that further expansion of the optimization strategies mentioned above will maximize the potential of lead-free perovskite PDs.

### Stability and Reliability

Despite the unique optical and optoelectronic properties of perovskite materials, their instability remains a key challenge for application in optoelectronic devices, stemming from inherent structural instability and defects that accelerate degradation. Defects introduced during the preparation of perovskite materials remain the primary bottleneck constraining photodetection performance. SCs materials exhibit significantly fewer defects due to their reduced amorphous boundaries and fewer pinholes, thereby enabling lower dark currents, higher responsivities, and faster response speeds. Based on existing solution-processed techniques, a series of approaches, such as optimizing crystal growth processes to obtain perovskites with perfect lattice structures, or employing additive engineering to introduce ionic additives for defect passivation or reducing agents for antioxidation to minimize trap states, will contribute to improve device performance. Whether in SCs or polycrystalline thin-film forms, perovskite materials still require further in-depth exploration of defect passivation engineering [129]. It should be noted, however, that the passivation effects of certain functional groups or ions have been repeatedly confirmed and those studies that replacing similar materials to passivate defects without significant improvement is undesirable, as such approaches essentially employ similar passivation mechanisms. Attention should be focused on materials or techniques offering innovative perspectives and robust passivation performance, representing the correct direction for the performance optimization of perovskite PDs. Additionally, the application of perovskite PDs in the field of wearable flexible electronics (such as physiological signal detection) places higher demands on their stability and reliability. Beyond stability in aqueous environments, it is necessary to consider the compatibility of perovskite materials with flexible substrates (PET, PI) as well as mechanical stress issues during dynamic stretching [227]. Therefore, future research should not be limited to static environmental stability but should focus on mechanical robustness under multiphysics coupling. This could involve introducing functional additives to strengthen grain boundaries, utilizing flexible encapsulation layers to achieve stress-neutral balance or integrating perovskite PDs with organic elastomers to mitigate and repair mechanical damage during deformation.

Encapsulation technology has been demonstrated as an effective solution for enhancing stability and reliability in perovskite photovoltaics, and its integration with PDs can effectively suppress water–oxygen erosion. Currently, there are still limited discussions regarding high-performance encapsulation materials and encapsulation systems suitable for perovskite PDs. High-performance encapsulation materials and with high light transmittance, low elastic modulus, suitable thermal expansion coefficient, high mechanical strength, and excellent chemical inertness need to be explored to meet the long-term devices stability requirements [260]. Exploring top-layer functional materials with encapsulation effects or self-sealing materials would be highly beneficial, as this would also enhance device performance and water resistance. In terms of encapsulation systems, the integration of top encapsulation barriers with edge sealing techniques has demonstrated superior reliability. Meanwhile, it is essential to comprehensively evaluate suitable encapsulation materials, low-temperature processing conditions, and cost-effective encapsulation processes, combining multiple encapsulation materials and strategies to deliver enhanced stability improvements for commercial application requirements.

### Large-Scale Uniformity and Production

The large-area uniformity of perovskite films significantly impacts the future development of scaled manufacturing, as variations in film quality directly affect the sensitivity of PDs and the accuracy of region of interest (ROI) signal readout. Achieving wafer-level uniformity and enabling hetero-integration with existing semiconductors represent critical challenges for metal halide perovskites in optoelectronic applications. Spin coating, the most prevalent method for perovskite film preparation, suffers from coffee-ring effects and crystallization inhomogeneities, while the detrimental impact of organic solvents on surface flatness represents an unavoidable limitation, confining its application to small-area device performance studies. Techniques such as squeegee coating and inkjet printing demonstrate significant potential for producing uniform, large-area films. It is important to integrate these methods with additive engineering and interface engineering to enhance the crystallinity and optoelectronic properties of perovskite films fabricated via these approaches. The entirely dry preparation of perovskite devices, such as vacuum evaporation and chemical vapor deposition, also represents a promising pathway. Vacuum evaporation technology holds considerable promise for repeatable and reliable experimentation due to its substrate-independent nature, high controllability of film thickness, and advantages in multi-layer stacking of different materials. More importantly, it has proved to be an effective method for industrial-scale mass production [41]. Although vacuum deposition facilitates the preparation of fully-covered, uniform and large-area perovskite films, complex vacuum chamber configurations, low deposition rates required for uniformity, lengthy reaction times, and challenging crystallinity control could hinder the low-cost manufacturing of scalable perovskite PDs. The space-confined methods offer a unique evolutionary path due to its size tunability. By guiding perovskite crystallization using physical templates, it is not only possible to obtain single-crystal films with higher intrinsic mobility, but more importantly, the physical isolation of pixel units is achieved through the geometric confinement provided by the templates. This pixel-level confinement fundamentally resolves the noise and inhomogeneity caused by random grain boundaries in large-area polycrystalline films, offering a promising solution for commercial imaging chips with ultra-low dark current and high uniformity. However, this approach shifts the challenge of direct perovskite film deposition to the fabrication of high-precision, large-area templates and the control of single-crystal growth kinetics. Ultimately, large-scale fabrication and industrial mass production depend on the reproducibility of the process and its compatibility with micro- and nanofabrication. For different scale factors, performance requirements, and application scenarios, the appropriate strategy must be selected from among those outlined above.

### Commercialization Application Pathways and Standardized Testing

PDs are approaching practical applications in many fields, such as imaging, optical communications, X-ray monitoring, and biomedical sensing. With the rapid improvement in the performance of perovskite PDs, simply comparing device performance metrics like responsivity and detectivity is losing its academic significance. Integrating perovskite PDs into specific application systems and further combining them with commercial systems is of greater value and should be prioritized. Optimizing the key performance indicators for specific application scenarios (e.g., medical diagnostics, biosensing, wearable devices) and addressing the core challenges (e.g., stability, large-area fabrication) in those scenarios are of more critical significance for development. For example, the integration of perovskite PDs with readout integrated circuits (ROICs) holds significant promise for high-resolution imaging, multispectral sensing, and artificial vision applications, yet the complex device architecture and challenging integration process present bottlenecks to their adoption in commercial imaging sensors. The intricate surface topography of ROIC substrates severely prevents the deposition of high-quality and uniform perovskite films. Furthermore, compatibility with conventional photolithography processes must be addressed, since it is an essential technique for integrating PDs in multispectral imaging systems. This incompatibility stems from the irreversible dissolution and degradation of perovskites in polar solvents. In recent studies, representative solutions have included employing orthogonal photolithography using fluoride-based solvents or utilizing protective layers (such as parylene) to prevent damage to the perovskite layer during photolithography, enabling subsequent patterning and etching operations [106]. These promising approaches are driving the large-scale integration of perovskite PDs.

After addressing the large-scale fabrication of perovskite PDs, it is strongly recommended that future research adheres to benchmarking protocols when reporting performance metrics to promote standardization and comparability in this field and to facilitate commercialization. Specifically, the following standards should be followed as much as possible [261]: (i) Clearly specify the precise wavelength of the test light source and the calibrated range of absolute light power density; (ii) when reporting extremely high detectivity values, provide measured dark-field noise spectral density (NSD) curves rather than relying on simple formulaic calculations; (iii) clearly define the effective photosensitive area (rather than merely the electrode overlap area) and provide detailed specifications of the bias conditions; (iv) conduct fair comparisons under the same bandwidth and similar bias conditions.

In addition to these traditional imaging technologies, perovskite materials offer unique physical advantages for achieving intrinsic polarization-sensitive detection, such as the fabrication of sub-micron and nanoscale structures and chiral crystals. This polarization detection provides information on polarization states in addition to light intensity and wavelength, applicable to industrial non-destructive testing in complex environments, high-contrast machine vision, and three-dimensional depth reconstruction, thereby establishing a differentiated competitive path compared to commercial silicon-based detectors. Beyond perception, visual processing in the age of artificial intelligence is gradually evolving toward in-sensor computing and biomimetic architectures. Benefiting from the tunable charge carrier dynamics, perovskite PDs show application potential in the field of biomimetic retinal imaging, offering an alternative to traditional physically decoupled visual processing architectures and meeting the stringent demands for extreme energy efficiency in future edge computing [262]. However, it must be noted that current advancements still remain at the stage of laboratory demonstrations, as it has focused solely on functional simulation and lacks unified evaluation criteria, such as circuit crosstalk, hardware integration scale, large-area array yield, and peripheral circuit energy efficiency. Moving toward standardization and compatibility with silicon-based integrated circuits, while adapting and drawing upon their rigorous and mature evaluation standards, represents the direction that should be strongly encouraged for future research on biomimetic neuromorphic vision based on perovskite PDs.

For health monitoring, perovskite PDs must not only focus on their detection capabilities but also ensure reliability, environmental-friendliness, human body compatibility, and excellent flexibility and extensibility to address the shortcomings of traditional inorganic photodetection systems in the commercial industry. The same issue arises in perovskite-based X-ray detection applications. Perovskite polycrystalline films can also be synthesized with large surface areas, which would meet the requirements for commercializing devices. However, the required thickness of perovskite films is ideally within the range of several hundred micrometers to ensure comprehensive absorption of X-ray radiation. How to balance flexibility, thickness, and surface area to accommodate practical applications warrants more emphasis. Novel approaches, such as employing halide perovskite materials containing high-Z atoms, advanced thin-film fabrication techniques, and utilizing template-assisted growth to achieve halide perovskite layers with high thickness characteristics, are worthy of further investigation, potentially representing significant breakthroughs in achieving large-scale, high-efficiency perovskite X-ray detectors.

Moreover, it should be pointed out that, regarding perovskite PDs, authoritative and standardized testing and evaluation standards remain lacking for certain critical parameters, such as device stability and reliability, which leads to an absence of comparative analysis between different devices and hinders the identification of precise optimization directions. Particularly in the field of cutting-edge neuromorphic/bionic PDs, current research remains focused on demonstrating functionality without establishing key metrics to evaluate the completeness of these functions. This approach risks diverting research efforts and hindering genuine commercialization progress. To meet the demands of large-scale commercial applications, standardized testing protocols and evaluation metrics ought to be established in the future.

### Conclusion

In summary, the development of perovskite PDs over the past decade has integrated interdisciplinary efforts spanning chemistry, materials science, physics, and electronic information, representing consistently sustained innovation in optoelectronics from materials to heterostructure architectures. Chemists are dedicated to enhancing the stability of perovskites and identifying lead-free alternatives, while physicists focus on device microstructure and interface design to maximize the utilization of absorbed photons. Engineers, meanwhile, develop hetero-integration architectures that enable perovskite systems to be compatible with existing silicon-based systems.

Looking ahead, the advancements in perovskite PDs should strike a balance between non-toxicity, stability, performance enhancement, functional expansion, and large-scale integration. The superior optoelectronic properties of perovskite materials stem from their unique physical structure, which inherently carries instability and lead-free difficulty. To that end, the solutions should be sought while acknowledging this challenge. Researches on performance and stability that is repetitive, mechanistically similar, and lacks significant breakthroughs should be reduced, focusing instead on device innovations with underlying mechanism advancements and practical application requirements. Efforts to advance the mass production and commercial integration for perovskite PDs should be encouraged, accompanied by exploring advanced functionalities. It is reasonable to believe that deeper exploration and the research focus adjustments would enable perovskite PDs to be truly applied in commercial consumer electronics to break through existing photodetection performance barriers, establishing a more sensitive, efficient, and intelligent optoelectronic sensing paradigm.

## References

[1] S. Mauthe, Y. Baumgartner, M. Sousa, Q. Ding, M.D. Rossell et al., High-speed III-V nanowire photodetector monolithically integrated on Si. Nat. Commun. **11**(1), 4565 (2020). 10.1038/s41467-020-18374-z PMC7486389 32917898

[2] J. Yang, W. Wang, C. Bao, W. Huang, J. Wang, Toward practical applications of perovskite photodetectors: advantages and challenges. Matter **8**(7), 102207 (2025). 10.1016/j.matt.2025.102207

[3] Y. Ma, X. Xu, T. Li, Z. Wang, N. Li et al., Amplified narrowband perovskite photodetectors enabled by independent multiplication layers for anti-interference light detection. Sci. Adv. **11**(22), eadq1127 (2025). 10.1126/sciadv.adq1127 PMC12124361 40446029

[4] A. Kojima, K. Teshima, Y. Shirai, T. Miyasaka, Organometal halide perovskites as visible-light sensitizers for photovoltaic cells. J. Am. Chem. Soc. **131**(17), 6050–6051 (2009). 10.1021/ja809598r 19366264

[5] Y. Li, Z. Shi, W. Liang, J. Ma, X. Chen et al., Recent advances toward environment-friendly photodetectors based on lead-free metal halide perovskites and perovskite derivatives. Mater. Horiz. **8**(5), 1367–1389 (2021). 10.1039/d0mh01567a 34846447

[6] Y. Wang, R. Lin, C. Liu, X. Wang, C. Chosy et al., Homogenized contact in all-perovskite tandems using tailored 2D perovskite. Nature **635**(8040), 867–873 (2024). 10.1038/s41586-024-08158-6 39401514

[7] C. Liu, Y. Yang, H. Chen, I. Spanopoulos, A.S.R. Bati et al., Two-dimensional perovskitoids enhance stability in perovskite solar cells. Nature **633**(8029), 359–364 (2024). 10.1038/s41586-024-07764-8 38977018

[8] L. Wang, Y. Xue, M. Cui, Y. Huang, H. Xu et al., A chiral reduced-dimension perovskite for an efficient flexible circularly polarized light photodetector. Angew. Chem. Int. Ed. **59**(16), 6442–6450 (2020). 10.1002/anie.201915912 31994286

[9] Z. Li, X. Liu, C. Zuo, W. Yang, X. Fang, Supersaturation-controlled growth of monolithically integrated lead-free halide perovskite single-crystalline thin film for high-sensitivity photodetectors. Adv. Mater. **33**(41), 2170324 (2021). 10.1002/adma.202170324 34431141

[10] X. Hu, X. Zhang, L. Liang, J. Bao, S. Li et al., High-performance flexible broadband photodetector based on organolead halide perovskite. Adv. Funct. Mater. **24**(46), 7373–7380 (2014). 10.1002/adfm.201402020

[11] C. Xie, C.-K. Liu, H.-L. Loi, F. Yan, Perovskite-based phototransistors and hybrid photodetectors. Adv. Funct. Mater. **30**(20), 1903907 (2020). 10.1002/adfm.201903907

[12] X. Geng, F. Wang, H. Tian, Q. Feng, H. Zhang et al., Ultrafast photodetector by integrating perovskite directly on silicon wafer. ACS Nano **14**(3), 2860–2868 (2020). 10.1021/acsnano.9b06345 32027117

[13] Q. Fan, G.V. Biesold-McGee, J. Ma, Q. Xu, S. Pan et al., Lead-free halide perovskite nanocrystals: crystal structures, synthesis, stabilities, and optical properties. Angew. Chem. Int. Ed. **59**(3), 1030–1046 (2020). 10.1002/anie.201904862 31087754

[14] L. Chu, W. Ahmad, W. Liu, J. Yang, R. Zhang et al., Lead-free halide double perovskite materials: a new superstar toward green and stable optoelectronic applications. Nano-Micro Lett. **11**(1), 16 (2019). 10.1007/s40820-019-0244-6 PMC7770810 34137969

[15] M. Gedda, H. Song, A.R. Pininti, O. Alkhazragi, H. Faber et al., High-speed, self-powered 2D-perovskite photodetectors with exceptional ambient stability enabled by planar nanocavity engineering. Mater. Sci. Eng. R. Rep. **162**, 100885 (2025). 10.1016/j.mser.2024.100885

[16] S. Han, S. Zhu, G. Fan, X. Huang, G. Zhao et al., Halide tunable perovskite single crystals as efficient photodetector. Adv. Funct. Mater. **35**(17), 2421770 (2025). 10.1002/adfm.202421770

[17] S. Zhang, Y. Ge, X. Qin, X. Wang, T. Tao et al., Vacuum-assisted growth of single crystal perovskite arrays toward ultralow dark current photodetectors. Adv. Funct. Mater. **35**(14), 2418968 (2025). 10.1002/adfm.202418968

[18] Z. Lv, G. Pan, S. Wang, Y. Xie, H. Tang et al., High-performance near-infrared photodetector enabled by tin-based perovskite inverse opal towards secure information transmission. Adv. Funct. Mater. **36**(47), e75511 (2026). 10.1002/adfm.75511

[19] Y.-C. Fang, Y.-H. Huang, W. Wei, S.-Y. Zou, C.-Y. Sheng et al., Constructing reverse electric field by buried interfacial heterojunction engineering enables high-performance perovskite X-ray detectors. Matter **9**(5), 102677 (2026). 10.1016/j.matt.2026.102677

[20] Y. Duan, R. Yu, H. Zhang, W. Yang, W. Xie et al., Programmable, high-resolution printing of spatially graded perovskites for multispectral photodetectors. Adv. Mater. **36**(24), e2313946 (2024). 10.1002/adma.202313946 38582876

[21] L. Li, A. Gao, C. Sun et al., *In situ* ionic liquid oligomer encapsulation enabled robust perovskite single crystals toward high-efficiency X-ray detection. ACS Energy Lett. **11**(3), 2993–3002 (2026). 10.1021/acsenergylett.6c00267

[22] C. Ge, C. Deng, J. Zhu, Y. Zhu, Q. Xu et al., A near-infrared Sn-Pb perovskite imager with monolithic integration. Light Sci. Appl. **15**(1), 73 (2026). 10.1038/s41377-025-02127-y PMC12815914 41554699

[23] X. Pan, L. Ding, Application of metal halide perovskite photodetectors. J. Semicond. **43**(2), 020203 (2022). 10.1088/1674-4926/43/2/020203

[24] H. Wang, Y. Sun, J. Chen, F. Wang, R. Han et al., A review of perovskite-based photodetectors and their applications. Nanomaterials **12**(24), 4390 (2022). 10.3390/nano12244390 PMC9784743 36558241

[25] L. Liu, S.-Y. Liu, Y. Shi, C.-L. Fang, S. Zhao et al., Anti-perovskites with long carrier lifetime for ultralow dose and stable X-ray detection. Nat. Photonics **18**(9), 990–997 (2024). 10.1038/s41566-024-01482-3

[26] Y. Liang, Q. Lu, W. Wu, Z. Xu, H. Lu et al., A universal fabrication strategy for high-resolution perovskite-based photodetector arrays. Small Meth. **7**(9), 2300339 (2023). 10.1002/smtd.202300339 37199230

[27] R. Ollearo, J. Wang, M.J. Dyson, C.H.L. Weijtens, M. Fattori et al., Ultralow dark current in near-infrared perovskite photodiodes by reducing charge injection and interfacial charge generation. Nat. Commun. **12**(1), 7277 (2021). 10.1038/s41467-021-27565-1 PMC8671406 34907190

[28] T. Maschwitz, L. Merten, F. Ünlü, M. Majewski, F. Haddadi Barzoki et al., How crystallization additives govern halide perovskite grain growth. Nat. Commun. **16**, 9894 (2025). 10.1038/s41467-025-65484-7 PMC12603034 41213992

[29] Z. He, B. Sun, H. Lu, X. Sun, Z. Xu et al., Focus-tunable real-time imaging system based on ultrathin perovskite curved image sensor with hierarchical mesh design. Sci. Adv. **11**(36), eadw7826 (2025). 10.1126/sciadv.adw7826 PMC12407062 40901939

[30] C. Bao, W. Xu, J. Yang, S. Bai, P. Teng et al., Bidirectional optical signal transmission between two identical devices using perovskite diodes. Nat. Electron. **3**(3), 156–164 (2020). 10.1038/s41928-020-0382-3 PMC7100905 32226921

[31] H. Li, Y. Ma, Z. Liang, Z. Wang, Y. Cao et al., Wearable skin-like optoelectronic systems with suppression of motion artifacts for cuff-less continuous blood pressure monitor. Natl. Sci. Rev. **7**(5), 849–862 (2020). 10.1093/nsr/nwaa022 PMC8288864 34692108

[32] X. Hu, S. Luo, J. Leng, C. Wang, Y. Chen et al., Density-discriminating chromatic X-ray imaging based on metal halide nanocrystal scintillators. Sci. Adv. **9**(37), eadh5081 (2023). 10.1126/sciadv.adh5081 PMC10881070 37713492

[33] S. Tsarev, D. Proniakova, X. Liu, E. Wu, G.J. Matt et al., Vertically stacked monolithic perovskite colour photodetectors. Nature **642**(8068), 592–598 (2025). 10.1038/s41586-025-09062-3 PMC12176651 40533540

[34] Y. Li, Z. Shi, W. Liang, L. Wang, S. Li et al., Highly stable and spectrum-selective ultraviolet photodetectors based on lead-free copper-based perovskites. Mater. Horiz. **7**(2), 530–540 (2020). 10.1039/c9mh01371g

[35] W. Cheng, W. Tian, F. Cao, L. Li, Self-powered bifunctional perovskite photodetectors with both broadband and narrowband photoresponse. InfoMat **4**(11), e12348 (2022). 10.1002/inf2.12348

[36] Z. Guo, J. Zhang, X. Liu, L. Wang, L. Xiong et al., Optoelectronic synapses and photodetectors based on organic semiconductor/halide perovskite heterojunctions: materials, devices, and applications. Adv. Funct. Mater. **33**(46), 2305508 (2023). 10.1002/adfm.202305508

[37] Y. Cheng, H. Wan, E.H. Sargent, D. Ma, Reduced-dimensional perovskites: quantum well thickness distribution and optoelectronic properties. Adv. Mater. **37**(25), e2410633 (2025). 10.1002/adma.202410633 39295466

[38] A. Biswas, R. Bakthavatsalam, S.R. Shaikh, A. Shinde, A. Lohar et al., Efficient broad-band emission from contorted purely corner-shared one dimensional (1D) organic lead halide perovskite. Chem. Mater. **31**(7), 2253–2257 (2019). 10.1021/acs.chemmater.9b00069

[39] Y. Li, Z. Shi, L. Wang, Y. Chen, W. Liang et al., Solution-processed one-dimensional CsCu_2_I_3_nanowires for polarization-sensitive and flexible ultraviolet photodetectors. Mater. Horiz. **7**(6), 1613–1622 (2020). 10.1039/d0mh00250j

[40] W.-F. Zhang, W.-J. Pan, T. Xu, R.-Y. Song, Y.-Y. Zhao et al., One-dimensional face-shared perovskites with broad-band bluish white-light emissions. Inorg. Chem. **59**(19), 14085–14092 (2020). 10.1021/acs.inorgchem.0c01861 32926625

[41] N. Jiang, H. Chu, Z. Pan, H. Pan, S. Zhao et al., One-step fabrication of 0D Cs_4_PbBr_6_ perovskite with nonlinear optical properties for ultrafast pulse generation. Adv. Sci. **11**(35), 2404465 (2024). 10.1002/advs.202404465 PMC11425289 38995100

[42] Y. Zhang, Y. Liu, Z. Xu, H. Ye, Z. Yang et al., Nucleation-controlled growth of superior lead-free perovskite Cs_3_Bi_2_I_9_ single-crystals for high-performance X-ray detection. Nat. Commun. **11**, 2304 (2020). 10.1038/s41467-020-16034-w PMC7210296 32385231

[43] C. Zhang, J. Chen, S. Wang, L. Kong, S.W. Lewis et al., Metal halide perovskite nanorods: shape matters. Adv. Mater. **32**(46), 2002736 (2020). 10.1002/adma.202002736 32985008

[44] J. Shamsi, A.S. Urban, M. Imran, L. De Trizio, L. Manna, Metal halide perovskite nanocrystals: synthesis, post-synthesis modifications, and their optical properties. Chem. Rev. **119**(5), 3296–3348 (2019). 10.1021/acs.chemrev.8b00644 PMC6418875 30758194

[45] M. Saliba, T. Matsui, K. Domanski, J.-Y. Seo, A. Ummadisingu et al., Incorporation of rubidium cations into perovskite solar cells improves photovoltaic performance. Science **354**(6309), 206–209 (2016). 10.1126/science.aah5557 27708053

[46] M.M. Lee, J. Teuscher, T. Miyasaka, T.N. Murakami, H.J. Snaith, Efficient hybrid solar cells based on meso-superstructured organometal halide perovskites. Science **338**(6107), 643–647 (2012). 10.1126/science.1228604 23042296

[47] M. Abdi-Jalebi, Z. Andaji-Garmaroudi, S. Cacovich, C. Stavrakas, B. Philippe et al., Maximizing and stabilizing luminescence from halide perovskites with potassium passivation. Nature **555**(7697), 497–501 (2018). 10.1038/nature25989 29565365

[48] D.W. DeQuilettes, S.M. Vorpahl, S.D. Stranks, H. Nagaoka, G.E. Eperon et al., Impact of microstructure on local carrier lifetime in perovskite solar cells. Science **348**(6235), 683–686 (2015). 10.1126/science.aaa5333 25931446

[49] Q. Dong, Y. Fang, Y. Shao, P. Mulligan, J. Qiu et al., Electron-hole diffusion lengths > 175 μm in solution-grown CH_3_NH_3_PbI_3_ single crystals. Science **347**(6225), 967–970 (2015). 10.1126/science.aaa5760 25636799

[50] C. Bi, X. Zheng, B. Chen, H. Wei, J. Huang, Spontaneous passivation of hybrid perovskite by sodium ions from glass substrates: mysterious enhancement of device efficiency revealed. ACS Energy Lett. **2**(6), 1400–1406 (2017). 10.1021/acsenergylett.7b00356

[51] Z. Xiao, Z. Song, Y. Yan, From lead halide perovskites to lead-free metal halide perovskites and perovskite derivatives. Adv. Mater. **31**(47), 1803792 (2019). 10.1002/adma.201803792 30680809

[52] Y. Zhang, Y. Ma, Y. Wang, X. Zhang, C. Zuo et al., Lead-free perovskite photodetectors: progress, challenges, and opportunities. Adv. Mater. **33**(26), e2006691 (2021). 10.1002/adma.202006691 34028107

[53] E. Jokar, L. Cai, J. Han, E.J.C. Nacpil, I. Jeon, Emerging opportunities in lead-free and lead–tin perovskites for environmentally viable photodetector applications. Chem. Mater. **35**(9), 3404–3426 (2023). 10.1021/acs.chemmater.3c00345

[54] B.-W. Park, B. Philippe, X. Zhang, H. Rensmo, G. Boschloo et al., Bismuth based hybrid perovskites A_3_Bi_2_I_9_ (A: methylammonium or cesium) for solar cell application. Adv. Mater. **27**(43), 6806–6813 (2015). 10.1002/adma.201501978 26418187

[55] C. Wu, Q. Zhang, G. Liu, Z. Zhang, D. Wang et al., From Pb to Bi: a promising family of Pb-free optoelectronic materials and devices. Adv. Energy Mater. **10**(13), 1902496 (2020). 10.1002/aenm.201902496

[56] W.-J. Yin, J.-H. Yang, J. Kang, Y. Yan, S.-H. Wei, Halide perovskite materials for solar cells: a theoretical review. J. Mater. Chem. A **3**(17), 8926–8942 (2015). 10.1039/c4ta05033a

[57] W. Kim, H. Kim, T.J. Yoo, J.Y. Lee, J.Y. Jo et al., Perovskite multifunctional logic gates *via* bipolar photoresponse of single photodetector. Nat. Commun. **13**(1), 720 (2022). 10.1038/s41467-022-28374-w PMC8821588 35132055

[58] Y. Wei, Z. Cheng, J. Lin, An overview on enhancing the stability of lead halide perovskite quantum dots and their applications in phosphor-converted LEDs. Chem. Soc. Rev. **48**(1), 310–350 (2019). 10.1039/c8cs00740c 30465675

[59] J.A. Christians, P.A. Miranda Herrera, P.V. Kamat, Transformation of the excited state and photovoltaic efficiency of CH_3_NH_3_PbI_3_ perovskite upon controlled exposure to humidified air. J. Am. Chem. Soc. **137**(4), 1530–1538 (2015). 10.1021/ja511132a 25590693

[60] A.M.A. Leguy, Y. Hu, M. Campoy-Quiles, M.I. Alonso, O.J. Weber et al., Reversible hydration of CH_3_NH_3_PbI_3_ in films, single crystals, and solar cells. Chem. Mater. **27**(9), 3397–3407 (2015). 10.1021/acs.chemmater.5b00660

[61] M. Kulbak, S. Gupta, N. Kedem, I. Levine, T. Bendikov et al., Cesium enhances long-term stability of lead bromide perovskite-based solar cells. J. Phys. Chem. Lett. **7**(1), 167–172 (2016). 10.1021/acs.jpclett.5b02597 26700466

[62] N. Aristidou, C. Eames, I. Sanchez-Molina, X. Bu, J. Kosco et al., Fast oxygen diffusion and iodide defects mediate oxygen-induced degradation of perovskite solar cells. Nat. Commun. **8**, 15218 (2017). 10.1038/ncomms15218 PMC5437277 28492235

[63] K. Chen, S. Schünemann, S. Song, H. Tüysüz, Structural effects on optoelectronic properties of halide perovskites. Chem. Soc. Rev. **47**(18), 7045–7077 (2018). 10.1039/c8cs00212f 30101254

[64] Y.H. Lee, W.-J. Lee, G.S. Lee, J.Y. Park, B. Yuan et al., Large-scale 2D perovskite nanocrystals photodetector array *via* ultrasonic spray synthesis. Adv. Mater. **37**(12), 2417761 (2025). 10.1002/adma.202417761 PMC11938026 39967441

[65] J.-W. Lee, Z. Dai, C. Lee, H.M. Lee, T.-H. Han et al., Tuning molecular interactions for highly reproducible and efficient formamidinium perovskite solar cells *via* adduct approach. J. Am. Chem. Soc. **140**(20), 6317–6324 (2018). 10.1021/jacs.8b01037 29723475

[66] J.G. Tait, S. Manghooli, W. Qiu, L. Rakocevic, L. Kootstra et al., Rapid composition screening for perovskite photovoltaics *via* concurrently pumped ultrasonic spray coating. J. Mater. Chem. A **4**(10), 3792–3797 (2016). 10.1039/c6ta00739b

[67] T.A. Cohen, D. Sharp, K.T. Kluherz, Y. Chen, C. Munley et al., Direct patterning of perovskite nanocrystals on nanophotonic cavities with electrohydrodynamic inkjet printing. Nano Lett. **22**(14), 5681–5688 (2022). 10.1021/acs.nanolett.2c00473 35819950

[68] L. Zhou, Y.-F. Xu, B.-X. Chen, D.-B. Kuang, C.-Y. Su, Synthesis and photocatalytic application of stable lead-free Cs_2_AgBiBr_6_ perovskite nanocrystals. Small **14**(11), 1703762 (2018). 10.1002/smll.201703762 29380522

[69] F. Yao, J. Peng, R. Li, W. Li, P. Gui et al., Room-temperature liquid diffused separation induced crystallization for high-quality perovskite single crystals. Nat. Commun. **11**, 1194 (2020). 10.1038/s41467-020-15037-x PMC7055282 32132533

[70] P. Fan, D. Gu, G.-X. Liang, J.-T. Luo, J.-L. Chen et al., High-performance perovskite CH_3_NH_3_PbI_3_ thin films for solar cells prepared by single-source physical vapour deposition. Sci. Rep. **6**, 29910 (2016). 10.1038/srep29910 PMC4947921 27426686

[71] H. Li, L. Tan, C. Jiang, M. Li, J. Zhou et al., Molten salt strategy for reproducible evaporation of efficient perovskite solar cells. Adv. Funct. Mater. **33**(10), 2211232 (2023). 10.1002/adfm.202211232

[72] Y. Li, Z. Shi, L. Lei, F. Zhang, Z. Ma et al., Highly stable perovskite photodetector based on vapor-processed micrometer-scale CsPbBr_3_ microplatelets. Chem. Mater. **30**(19), 6744–6755 (2018). 10.1021/acs.chemmater.8b02435

[73] B. Gao, J. Hu, S. Tang, X. Xiao, H. Chen et al., Organic-inorganic perovskite films and efficient planar heterojunction solar cells by magnetron sputtering. Adv. Sci. **8**(22), 2102081 (2021). 10.1002/advs.202102081 PMC8596124 34528412

[74] J.-K. Hwang, S.-H. Jeong, D. Kim, H.-S. Lee, Y. Kang, A review on dry deposition techniques: pathways to enhanced perovskite solar cells. Energies **16**(16), 5977 (2023). 10.3390/en16165977

[75] L. Li, J. Liu, M. Zeng, L. Fu, Space-confined growth of metal halide perovskite crystal films. Nano Res. **14**(6), 1609–1624 (2021). 10.1007/s12274-020-3050-z

[76] P. Moazzezi, V. Yeddu, S. Dayneko, Y. Haruta, M.R. Kokaba et al., Kinetics of space-confined inverse temperature crystallization: gradients enlarge thin perovskite single crystals. ACS Mater. Lett. **6**(8), 3557–3563 (2024). 10.1021/acsmaterialslett.4c00740

[77] S. Lan, Y. Peng, H. Shen, S. Wang, J. Ren et al., Seeds-assisted space-confined growth of all-inorganic perovskite arrays for ultralow-threshold single-mode lasing. Laser Photonics Rev. **15**(3), 2000428 (2021). 10.1002/lpor.202000428

[78] S. Pan, H. Zou, A.C. Wang, Z. Wang, J. Yu et al., Rapid capillary-assisted solution printing of perovskite nanowire arrays enables scalable production of photodetectors. Angew. Chem. Int. Ed. **59**(35), 14942–14949 (2020). 10.1002/anie.202004912 32394517

[79] H.-S. Rao, W.-G. Li, B.-X. Chen, D.-B. Kuang, C.-Y. Su, *In situ* growth of 120 Cm2 CH_3_NH_3_PbBr_3_ perovskite crystal film on FTO glass for narrowband-photodetectors. Adv. Mater. **29**(16), 1602639 (2017). 10.1002/adma.201602639 28220970

[80] Z. Chen, Q. Dong, Y. Liu, C. Bao, Y. Fang et al., Thin single crystal perovskite solar cells to harvest below-bandgap light absorption. Nat. Commun. **8**, 1890 (2017). 10.1038/s41467-017-02039-5 PMC5709415 29192232

[81] X. Fu, N. Dong, G. Lian, S. Lv, T. Zhao et al., High-quality CH_3_NH_3_PbI_3_ films obtained *via* a pressure-assisted space-confined solvent-engineering strategy for ultrasensitive photodetectors. Nano Lett. **18**(2), 1213–1220 (2018). 10.1021/acs.nanolett.7b04809 29389136

[82] G. Hu, J. Guo, J. Jiang, L. Wang, J. Zhang et al., Capillary condensation-driven growth of perovskite nanowire arrays for multi-functional photodetector. Light Sci. Appl. **14**(1), 61 (2025). 10.1038/s41377-024-01680-2 PMC11761479 39856055

[83] Z. Gu, Z. Huang, C. Li, M. Li, Y. Song, A general printing approach for scalable growth of perovskite single-crystal films. Sci. Adv. **4**(6), eaat2390 (2018). 10.1126/sciadv.aat2390 PMC6025903 29963635

[84] W. Shao, J.H. Kim, J. Simon, Z. Nian, S.-D. Baek et al., Molecular templating of layered halide perovskite nanowires. Science **384**(6699), 1000–1006 (2024). 10.1126/science.adl0920 38815024

[85] Y. Zhang, D. Kim, J.-H. Yun, J. Lim, M.-C. Jung et al., Self-assembled perovskite nanoislands on CH_3_NH_3_PbI_3_ cuboid single crystals by energetic surface engineering. Adv. Funct. Mater. **31**(50), 2105542 (2021). 10.1002/adfm.202105542

[86] K. Chen, H. Tüysüz, Morphology-controlled synthesis of organometal halide perovskite inverse opals. Angew. Chem. Int. Ed. **54**(46), 13806–13810 (2015). 10.1002/anie.201506367 26376773

[87] J. Sun, L. Ding, Linearly polarization-sensitive perovskite photodetectors. Nano-Micro Lett. **15**(1), 90 (2023). 10.1007/s40820-023-01048-y PMC10082144 37029229

[88] Z. Yu, A. Raman, S. Fan, Fundamental limit of nanophotonic light trapping in solar cells. Proc. Natl. Acad. Sci. U.S.A. **107**(41), 17491–17496 (2010). 10.1073/pnas.1008296107 PMC2955111 20876131

[89] M. Yang, X. Li, R. Wang, Y. Zhao, H. Wang, Plasmon-mediated wavelength-selective response enhancement in narrowband perovskite photodetectors. ACS Nano **19**(48), 40817–40827 (2025). 10.1021/acsnano.5c09195 41263531

[90] Z. Zhou, Q. Xue, Z. Yuan, B. Liu, D. Luo et al., 1-D linear photonic crystals enable narrow-band perovskite photodetectors by high-throughput optical model. IEEE Trans. Electron Devices **71**(3), 2005–2011 (2024). 10.1109/TED.2024.3362919

[91] Y. Wang, Y. Meng, S.-X. Li, B. Zou, High-performance and stable dual-nanograting-perovskite-based near-infrared photodetector. Adv. Mater. **38**(5), e15848 (2026). 10.1002/adma.202515848 41194322

[92] M.-Y. Li, K. Shen, H. Xu, A. Ren, J. Lee et al., Enhanced spatial light confinement of all inorganic perovskite photodetectors based on hybrid plasmonic nanostructures. Small **16**(46), 2004234 (2020). 10.1002/smll.202004234 33107207

[93] Y. Zhan, C. Li, Z. Che, H.C. Shum, X. Hu et al., Light management using photonic structures towards high-index perovskite optoelectronics: fundamentals, designing, and applications. Energy Environ. Sci. **16**(10), 4135–4163 (2023). 10.1039/d3ee00646h

[94] H. Kwon, J.W. Lim, D.H. Kim, Plasmonic perovskite photodetector with high photocurrent and low dark current mediated by Au NR/PEIE hybrid layer. J. Mater. Sci. Technol. **218**, 45–53 (2025). 10.1016/j.jmst.2024.08.033

[95] T. Wang, D. Zheng, J. Zhang, J. Qiao, C. Min et al., High-performance and stable plasmonic-functionalized formamidinium-based quasi-2D perovskite photodetector for potential application in optical communication. Adv. Funct. Mater. **32**(48), 2208694 (2022). 10.1002/adfm.202208694

[96] Y. Zhan, Y. Wang, Q. Cheng, C. Li, K. Li et al., A butterfly-inspired hierarchical light-trapping structure towards a high-performance polarization-sensitive perovskite photodetector. Angew. Chem. Int. Ed. **58**(46), 16456–16462 (2019). 10.1002/anie.201908743 31441973

[97] Y. Sun, J. Wang, J. Liu, H. Bao, J. Xu et al., Buried grating enables fast response self-powered polarization-sensitive perovskite photodetectors for high-speed optical communication and polarization imaging. Small **21**(17), 2411610 (2025). 10.1002/smll.202411610 40103493

[98] M. Li, S.K. Cushing, N. Wu, Plasmon-enhanced optical sensors: a review. Analyst **140**(2), 386–406 (2015). 10.1039/c4an01079e PMC4274271 25365823

[99] Y. Dong, Y. Gu, Y. Zou, J. Song, L. Xu et al., Improving all-inorganic perovskite photodetectors by preferred orientation and plasmonic effect. Small **12**(40), 5622–5632 (2016). 10.1002/smll.201602366 27552525

[100] Y.H. Lee, S.H. Lee, Y. Won, H. Kim, S.J. Yang et al., Boosting the performance of flexible perovskite photodetectors using hierarchical plasmonic nanostructures. Small Struct. **5**(7), 2300546 (2024). 10.1002/sstr.202300546

[101] Y. Ji, W. Xu, I.L. Rasskazov, H. Liu, J. Hu et al., Perovskite photonic crystal photoelectric devices. Appl. Phys. Rev. **9**(4), 041319 (2022). 10.1063/5.0106118

[102] S. Dou, H. Xu, J. Zhao, K. Zhang, N. Li et al., Bioinspired microstructured materials for optical and thermal regulation. Adv. Mater. **33**(6), 2000697 (2021). 10.1002/adma.202000697 32686250

[103] H. Oh, H.J. Kim, S. Kim, J.A. Kim, G. Kang et al., Highly flexible and stable perovskite/microbead hybrid photodetectors with improved interfacial light trapping. Appl. Surf. Sci. **544**, 148850 (2021). 10.1016/j.apsusc.2020.148850

[104] Y. Yue, N. Chai, M. Li, Z. Zeng, S. Li et al., Ultrafast photoexcitation induced passivation for quasi-2D perovskite photodetectors. Adv. Mater. **36**(33), e2407347 (2024). 10.1002/adma.202407347 38857569

[105] Y. Wu, S. Li, J. Ahmed, W. Tian, L. Li, Synergistic treatments of bulk and buried surface in tin-lead binary perovskite for efficient near-infrared photodetectors. Adv. Funct. Mater. **35**(33), 2501020 (2025). 10.1002/adfm.202501020

[106] Y. Mo, X. Huang, Q. Hu, C. Hu, P.K. Chu et al., Recent progress in perovskite photodetectors: from carrier dynamics to device structures and applications. Mater. Horiz. **12**(18), 7221–7243 (2025). 10.1039/d5mh00032g 40504227

[107] S. Xu, H. Zhang, H. Cui, H. Tao, L. Cheng et al., Enhanced performance in quasi-two-dimensional perovskite photodetectors by controlling the formation of a large N-phase. ACS Appl. Mater. Interfaces **17**(48), 65902–65910 (2025). 10.1021/acsami.5c07943 41261903

[108] X. Yin, Y. Zhao, C. Pei, Z. Wang, Y. Sun et al., Stress release of single crystal arrays bridged by SAM interface toward highly mechanically durable flexible perovskite NIR photodetector. Adv. Mater. **37**(12), 2413721 (2025). 10.1002/adma.202413721 39945064

[109] Y. Xi, G. Li, T. Ji, Y. Hao, S. Liu et al., Mitigating noise current in 2D perovskite single crystal photodetectors for imaging under black-light condition. Adv. Funct. Mater. **34**(45), 2407525 (2024). 10.1002/adfm.202407525

[110] H. Liu, L. Zhu, H. Zhang, X. He, F. Yan et al., Realizing high-detectivity near-infrared photodetectors in tin–lead perovskites by double-sided surface-preferred distribution of multifunctional tin thiocyanate additive. ACS Energy Lett. **8**(1), 577–589 (2023). 10.1021/acsenergylett.2c02055

[111] B. Bhardwaj, U. Bothra, S. Singh, S. Mills, T.J. Ronningen et al., Suppressing leakage current by interfacial engineering for highly sensitive, broadband, self-powered FACsPbI3 perovskite photodetectors. Appl. Phys. Rev. **10**(2), 021419 (2023). 10.1063/5.0153593

[112] C.-Y. Chang, Y.-L. Chin, C.-Y. Chang, J. Holovský, Facile interface engineering enabled efficient and stable self-powered perovskite photodetectors for versatile photosensing applications. Adv. Funct. Mater. **35**(20), 2422732 (2025). 10.1002/adfm.202422732

[113] F. Cao, L. Meng, M. Wang, W. Tian, L. Li, Gradient energy band driven high-performance self-powered perovskite/CdS photodetector. Adv. Mater. **31**(12), e1806725 (2019). 10.1002/adma.201806725 30697825

[114] L. Liu, F. Cao, L. Bian, M. Wang, H. Sun et al., Temperature-induced gradient tin–lead-mixed halide perovskite photodetector. Sci. China Mater. **66**(12), 4697–4703 (2023). 10.1007/s40843-023-2614-8

[115] Z. Zhan, D. Lin, J. Cai, Y. Lu, A. Chen et al., A perovskite photodetector crossbar array by vapor deposition for dynamic imaging. Adv. Mater. **34**(51), 2207106 (2022). 10.1002/adma.202207106 36193774

[116] J.Y. Chun, B.G. Kim, W. Jang, D.H. Wang, Loosening effect of perovskite intermolecular exchanger with strong steric hindrance for highly sensitive photodetector. Appl. Surf. Sci. **591**, 153207 (2022). 10.1016/j.apsusc.2022.153207

[117] L. Wu, Q. Cao, H. Li, X. Liu, W. Li et al., A 2D perovskite photodetector for NIR range and weak-light imaging applications *via* thermal regulation. Adv. Funct. Mater. **35**(48), 2505180 (2025). 10.1002/adfm.202505180

[118] Y.H. Lee, I. Song, S.H. Kim, J.H. Park, S.O. Park et al., Perovskite granular wire photodetectors with ultrahigh photodetectivity. Adv. Mater. **32**(32), e2002357 (2020). 10.1002/adma.202002357 32588494

[119] B. Wang, Y. Zou, H. Lu, W. Kong, S.C. Singh et al., Boosting perovskite photodetector performance in NIR using plasmonic bowtie nanoantenna arrays. Small **16**(24), 2001417 (2020). 10.1002/smll.202001417 32407005

[120] H.-L. Loi, J. Cao, X. Guo, C.-K. Liu, N. Wang et al., Gradient 2D/3D perovskite films prepared by hot-casting for sensitive photodetectors. Adv. Sci. **7**(14), 2000776 (2020). 10.1002/advs.202000776 PMC7375231 32714769

[121] W.-G. Li, X.-D. Wang, J.-F. Liao, Z.-F. Wei, Y.-F. Xu et al., A laminar MAPbBr_3_/MAPbBr_3–__*x*_I_*x*_ graded heterojunction single crystal for enhancing charge extraction and optoelectronic performance. J. Mater. Chem. C **7**(19), 5670–5676 (2019). 10.1039/c9tc00972h

[122] W. Ahmad, M.U. Rehman, Q. Zhuang, J. Zhang, W. Lei et al., Engineering in 2D violet phosphorus/PdSe_2_ van der Waals heterostructures for advanced optoelectronics. Commun. Mater. **7**, 102 (2026). 10.1038/s43246-026-01114-z

[123] W. Ahmad, J. Kazmi, M.Z. Nawaz, A. El Moutaouakil, J. Zhang et al., Interface engineering in van der Waals heterostructures: enhancing photodetector efficiency through structural and functional modifications. Adv. Funct. Mater. **36**(24), e16893 (2026). 10.1002/adfm.202516893

[124] W. Ahmad, U. Younis, M.Z. Nawaz, V. Laxmi, J. Zhang et al., Interface engineering with graphene contacts in VP/MoS_2_ heterostructures for high-performance, durable, and multispectral photodetection. ACS Appl. Mater. Interfaces **18**(11), 16767–16777 (2026). 10.1021/acsami.6c03721 41807358

[125] W. Ahmad, M.U. Rehman, M.Z. Nawaz, V. Laxmi, J. Huang et al., Type-I band engineering of van der Waals InSe/TiS_3_ heterostructures for broadband and polarization-sensitive photodetection with a large dichroic ratio. ACS Appl. Mater. Interfaces **17**(40), 56308–56319 (2025). 10.1021/acsami.5c13903 41021769

[126] W. Ahmad, M.U. Rehman, L. Pan, W. Li, J. Yi et al., Ultrasensitive near-infrared polarization photodetectors with violet phosphorus/InSe van der Waals heterostructures. ACS Appl. Mater. Interfaces **16**(15), 19214–19224 (2024). 10.1021/acsami.4c01396 38581080

[127] Q. Lin, A. Armin, D.M. Lyons, P.L. Burn, P. Meredith, Low noise, IR-blind organohalide perovskite photodiodes for visible light detection and imaging. Adv. Mater. **27**(12), 2060–2064 (2015). 10.1002/adma.201405171 25677496

[128] H. Zai, Y. Ma, Q. Chen, H. Zhou, Ion migration in halide perovskite solar cells: mechanism, characterization, impact and suppression. J. Energy Chem. **63**, 528–549 (2021). 10.1016/j.jechem.2021.08.006

[129] W. Fu, A.G. Ricciardulli, Q.A. Akkerman, R.A. John, M.M. Tavakoli et al., Stability of perovskite materials and devices. Mater. Today **58**, 275–296 (2022). 10.1016/j.mattod.2022.06.020

[130] J. Hidalgo, W. Kaiser, Y. An, R. Li, Z. Oh et al., Synergistic role of water and oxygen leads to degradation in formamidinium-based halide perovskites. J. Am. Chem. Soc. **145**(45), 24549–24557 (2023). 10.1021/jacs.3c05657 PMC10655111 37917967

[131] L. He, G. Hu, J. Jiang, W. Wei, X. Xue et al., Highly sensitive tin-lead perovskite photodetectors with over 450 days stability enabled by synergistic engineering for pulse oximetry system. Adv. Mater. **35**(10), 2210016 (2023). 10.1002/adma.202210016 36512669

[132] Y. Hong, S. Choi, J. Park, J.M. Kim, E. Kim et al., Metal halide perovskite photodetectors with hierarchical hybrid architectures: interface design for stability and optoelectric property enhancement. Chem. Eng. J. **507**, 160584 (2025). 10.1016/j.cej.2025.160584

[133] Y. Li, J. Xing, R. Zhou, Y. Li, Z. Huang et al., A (111) facet-dominated carbon dots-perovskite composite crystal matrix for highly stable photodetector. Small **21**(34), 2501181 (2025). 10.1002/smll.202501181 40574377

[134] D.H. Chun, S. Kim, J. Park, S. Lee, W. Kim et al., Nanopatterning on mixed halide perovskites for promoting photocurrent generation of flexible photodetector. Adv. Funct. Mater. **32**(43), 2206995 (2022). 10.1002/adfm.202206995

[135] Z. Yang, Y. Deng, X. Zhang, S. Wang, H. Chen et al., High-performance single-crystalline perovskite thin-film photodetector. Adv. Mater. **30**(8), 1704333 (2018). 10.1002/adma.201704333 29315842

[136] T. Liu, J. Wang, Y. Liu, L. Min, L. Wang et al., Cyano-coordinated tin halide perovskites for wearable health monitoring and weak light imaging. Adv. Mater. **36**(24), 2400090 (2024). 10.1002/adma.202400090 38433566

[137] Q. Ye, J. Zhang, P. Guo, H. Fan, D. Shchukin et al., Wet-chemical synthesis of surface-passivated halide perovskite microwires for improved optoelectronic performance and stability. ACS Appl. Mater. Interfaces **10**(50), 43850–43856 (2018). 10.1021/acsami.8b07428 30525368

[138] J. Wu, J. Lan, R. Wang, C. Cheng, W. Wang et al., Low-temperature encapsulation with silicone grease enhances efficiency and stability of perovskite solar cells *via* Pb0 defect passivation. Adv. Funct. Mater. **35**(40), 2425979 (2025). 10.1002/adfm.202425979

[139] Y. Shan, W. Cui, Y. Zhou, N. Li, X. Wang et al., Lead-free perovskite Cs_2_AgBiBr_6_ epitaxial thin films for high-performance and air-stable photodetectors. J. Mater. Chem. C **13**(18), 9072–9082 (2025). 10.1039/d4tc05407e

[140] Y. Zhang, E. Han, B.W. Zhang, J. Peng, J. Sun et al., 2D and quasi-2D hybrid halide perovskite single crystals: from fundamentals to functionalized photodetectors. Nano Energy **132**, 110368 (2024). 10.1016/j.nanoen.2024.110368

[141] J. Sun, R. Li, Y. Gui, X. Shao, J. Xue et al., Mechanistic insights and optimization strategies for perovskite single-crystal thin film growth. Chem. Sci. **16**(15), 6188–6202 (2025). 10.1039/d4sc08145e PMC11931432 40134661

[142] Y. Ma, X. Zhao, H. Chen, W. Wei, L. Shen, Progress and perspective of perovskite thin single crystal photodetectors. Acta Phys. Chim. Sin. **41**(4), 100030 (2025). 10.3866/PKU.WHXB202309045

[143] X. Li, C. Liu, F. Ding, Z. Lu, P. Gao et al., Ultra-stable and sensitive ultraviolet photodetectors based on monocrystalline perovskite thin films. Adv. Funct. Mater. **33**(15), 2213360 (2023). 10.1002/adfm.202213360

[144] S.-X. Li, Y.-S. Xu, C.-L. Li, Q. Guo, G. Wang et al., Perovskite single-crystal microwire-array photodetectors with performance stability beyond 1 year. Adv. Mater. **32**(28), e2001998 (2020). 10.1002/adma.202001998 32500553

[145] J. Yu, M. Wang, S. Lin, Probing the soft and nanoductile mechanical nature of single and polycrystalline organic–inorganic hybrid perovskites for flexible functional devices. ACS Nano **10**(12), 11044–11057 (2016). 10.1021/acsnano.6b05913 27935297

[146] N. Ma, J. Jiang, Y. Zhao, L. He, Y. Ma et al., Stable and sensitive tin-lead perovskite photodetectors enabled by azobenzene derivative for near-infrared acousto-optic conversion communications. Nano Energy **86**, 106113 (2021). 10.1016/j.nanoen.2021.106113

[147] Z. Zhang, W. Zhai, G. Li, W. Zheng, X. Li et al., Performance enhancement of tin-based perovskite photodetectors through bifunctional cesium fluoride engineering. ACS Appl. Mater. Interfaces **16**(10), 12773–12780 (2024). 10.1021/acsami.3c17687 38437709

[148] D. Wu, Y. Xu, H. Zhou, X. Feng, J. Zhang et al., Ultrasensitive, flexible perovskite nanowire photodetectors with long-term stability exceeding 5000 h. InfoMat **4**(9), e12320 (2022). 10.1002/inf2.12320

[149] T. Liu, Z. Yuan, L. Wang, C. Shan, Q. Zhang et al., Chelated tin halide perovskite for near-infrared neuromorphic imaging array enabling object recognition and motion perception. Nat. Commun. **16**(1), 4261 (2025). 10.1038/s41467-025-59624-2 PMC12059062 40335551

[150] K.-R. Yun, T.-J. Lee, S.-K. Kim, J.-H. Kim, T.-Y. Seong, Fast and highly sensitive photodetectors based on Pb-free Sn-based perovskite with additive engineering. Adv. Opt. Mater. **11**(1), 2201974 (2023). 10.1002/adom.202201974

[151] X. Yang, X.-D. Wang, W.-G. Li, Y.-H. Huang, L.-B. Wang et al., Conjugated diamine cation based halide perovskitoid enables robust stability and high photodetector performance. Sci. Bull. **69**(24), 3849–3859 (2024). 10.1016/j.scib.2024.08.041 39289049

[152] M. Wang, H. Sun, F. Cao, W. Tian, L. Li, Moisture-triggered self-healing flexible perovskite photodetectors with excellent mechanical stability. Adv. Mater. **33**(16), e2100625 (2021). 10.1002/adma.202100625 33734512

[153] L. Xiang, F. Gao, Y. Cao, D. Li, Q. Liu et al., Progress on the stability and encapsulation techniques of perovskite solar cells. Org. Electron. **106**, 106515 (2022). 10.1016/j.orgel.2022.106515

[154] L.J. Sutherland, H.C. Weerasinghe, G.P. Simon, A review on emerging barrier materials and encapsulation strategies for flexible perovskite and organic photovoltaics. Adv. Energy Mater. **11**(34), 2101383 (2021). 10.1002/aenm.202101383

[155] E.A. Gaulding, A.E. Louks, M. Yang, R. Tirawat, M.J. Wilson et al., Package development for reliability testing of perovskites. ACS Energy Lett. **7**(8), 2641–2645 (2022). 10.1021/acsenergylett.2c01168

[156] B.A.H. Huisman, C. Bordoni, A. Ciavatti, M. Sessolo, B. Fraboni et al., Laminated polymer-encapsulated halide perovskite photoconductors. Adv. Funct. Mater. **34**(1), 2308844 (2024). 10.1002/adfm.202308844

[157] H. Asgarimoghaddam, Q. Chen, F. Ye, A. Shahin, O.A.C. Marchione et al., Spatial atomic layer deposition of nitrogen-doped alumina thin films for high-performance perovskite solar cell encapsulation. Nano Energy **127**, 109782 (2024). 10.1016/j.nanoen.2024.109782

[158] Y. Zhan, Q. Cheng, J. Peng, Y. Zhao, F. Vogelbacher et al., Nacre inspired robust self-encapsulating flexible perovskite photodetector. Nano Energy **98**, 107254 (2022). 10.1016/j.nanoen.2022.107254

[159] L. Dou, Y. Yang, J. You, Z. Hong, W.-H. Chang et al., Solution-processed hybrid perovskite photodetectors with high detectivity. Nat. Commun. **5**, 5404 (2014). 10.1038/ncomms6404 25410021

[160] C. Liu, H. Peng, K. Wang, C. Wei, Z. Wang et al., PbS quantum dots-induced trap-assisted charge injection in perovskite photodetectors. Nano Energy **30**, 27–35 (2016). 10.1016/j.nanoen.2016.09.035

[161] C. Bao, J. Yang, S. Bai, W. Xu, Z. Yan et al., High performance and stable all-inorganic metal halide perovskite-based photodetectors for optical communication applications. Adv. Mater. **30**(38), 1803422 (2018). 10.1002/adma.201803422 30066403

[162] W. Ren, Q. Tan, Q. Wang, Y. Liu, Hybrid organolead halide perovskite microwire arrays/single CdSe nanobelt for a high-performance photodetector. Chem. Eng. J. **406**, 126779 (2021). 10.1016/j.cej.2020.126779

[163] Y. Zhao, Y. Qiu, H. Gao, J. Feng, G. Chen et al., Layered-perovskite nanowires with long-range orientational order for ultrasensitive photodetectors. Adv. Mater. **32**(9), e1905298 (2020). 10.1002/adma.201905298 31967709

[164] W. Cheng, X. He, J.-G. Wang, W. Tian, L. Li, N-(2-aminoethyl) acetamide additive enables phase-pure and stable α-FAPbI_3_ for efficient self-powered photodetectors. Adv. Mater. **34**(51), 2208325 (2022). 10.1002/adma.202208325 36245323

[165] Z.-L. Xiang, Q.-H. Tan, T. Zhu, P.-Z. Yang, Y.-P. Liu et al., High-performance 1D CsPbBr_3_/CdS photodetectors. Rare Met. **43**(11), 5932–5942 (2024). 10.1007/s12598-024-02741-6

[166] M. Jeong, S.G. Han, W. Sung, S. Kim, J. Min et al., Photomultiplication-type organic photodetectors with high EQE-bandwidth product by introducing a perovskite quantum dot interlayer. Adv. Funct. Mater. **33**(27), 2300695 (2023). 10.1002/adfm.202300695

[167] R. Liu, S. Pan, D. Wu, B. Hu, Z.-W. Zheng et al., Solvent-kinetics-regulated anti-solvent crystallization enables tunable-bandgap CsPbBr_*x*_Cl_3−__*x*_ perovskite microcrystal films for high-performance photodetectors. Adv. Opt. Mater. **14**(13), e02361 (2026). 10.1002/adom.202502361

[168] Y. Zhao, Y. Sun, C. Pei, X. Yin, X. Li et al., Low-temperature fabrication of stable black-phase CsPbI_3_ perovskite flexible photodetectors toward wearable health monitoring. Nano-Micro Lett. **17**(1), 63 (2024). 10.1007/s40820-024-01565-4 PMC11568160 39546239

[169] B.M. S, E. Klein, R. Lesyuk, C. Klinke, Toward high-performance photodetectors based on quasi-2D ruddlesden-popper mixed-n perovskite nanomaterials. Adv. Funct. Mater. **35**(9), 2416027 (2025). 10.1002/adfm.202416027

[170] Y. Shen, L. Luo, Y. Zhang, Y. Meng, Y. Yan et al., High-performance nanogap photodetectors based on 2D halide perovskites with a novel spacer cation. Adv. Funct. Mater. **34**(41), 2403746 (2024). 10.1002/adfm.202403746

[171] X. Zhang, R. Cheng, Y. Deng, Z. Liu, X. Liu et al., Tailoring performance of perovskite-based tunneling photodetector for portable monitoring of ultraviolet radiation risk. Nano Energy **122**, 109282 (2024). 10.1016/j.nanoen.2024.109282

[172] Q. Lv, X. Shen, X. Li, Y. Meng, K.M. Yu et al., On-wire design of axial periodic halide perovskite superlattices for high-performance photodetection. ACS Nano **18**(27), 18022–18035 (2024). 10.1021/acsnano.4c05205 38934514

[173] J. Ma, M. Zhang, C. Dun, H. Ji, Z. Ma et al., Highly polarization-sensitive solar-blind ultraviolet photodetection based on 1D Rb_2_CuCl_3_ microwires. Adv. Funct. Mater. **35**(10), 2414943 (2025). 10.1002/adfm.202414943

[174] M.A. Sadath, B. Guo, J. Li, D. Lin, E. Scalf et al., High-performance perovskite photodetector through plasmonic enhancement of carrier dynamics. ACS Nano **19**(48), 41147–41157 (2025). 10.1021/acsnano.5c14108 41314695

[175] K. Feng, Y. Li, Y. Chen, Z. Wei, G. Shen, High-performance 2D perovskite-based flexible photodetectors for optical communication and information encryption. Matter **8**(5), 101998 (2025). 10.1016/j.matt.2025.101998

[176] H. Hu, L. Ma, J. Zhao, C. Qi, W. Dai et al., Controlled growth of vertical-standing bandgap-tunable perovskite nanoplates for multicolor photodetectors. Adv. Funct. Mater. **36**(15), e19572 (2026). 10.1002/adfm.202519572

[177] L. Min, H. Sun, L. Guo, M. Wang, F. Cao et al., Frequency-selective perovskite photodetector for anti-interference optical communications. Nat. Commun. **15**(1), 2066 (2024). 10.1038/s41467-024-46468-5 PMC10920912 38453948

[178] W. Wang, W. Tian, F. Chen, J. Wang, W. Zhai et al., Filter-less color-selective photodetector derived from integration of parallel perovskite photoelectric response units. Adv. Mater. **36**(33), 2404968 (2024). 10.1002/adma.202404968 38897182

[179] G. Li, S. Li, J. Ahmed, W. Tian, L. Li, Flexible perovskite photodetector with room-temperature self-healing capability without external trigger. InfoMat **6**(11), e12594 (2024). 10.1002/inf2.12594

[180] L. Min, Y. Zhou, H. Sun, L. Guo, M. Wang et al., Carrier dynamic identification enables wavelength and intensity sensitivity in perovskite photodetectors. Light Sci. Appl. **13**(1), 280 (2024). 10.1038/s41377-024-01636-6 PMC11439907 39343785

[181] D. Nodari, L.J.F. Hart, O.J. Sandberg, F. Furlan, E. Angela et al., Dark current in broadband perovskite–organic heterojunction photodetectors controlled by interfacial energy band offset. Adv. Mater. **36**(33), 2401206 (2024). 10.1002/adma.202401206 38888509

[182] Y. Ma, L. Shan, Y. Ying, L. Shen, Y. Fu et al., Day-night imaging without infrared cutfilter removal based on metal-gradient perovskite single crystal photodetector. Nat. Commun. **15**(1), 7516 (2024). 10.1038/s41467-024-51762-3 PMC11362523 39209845

[183] M. Qiu, W. Li, Y. Luo, C. Zhao, Z. Zhao et al., Dual-sided field effect passivation for efficient and stable quasi-2D ruddlesden-popper perovskite photodetector. Adv. Funct. Mater. **35**(13), 2418064 (2025). 10.1002/adfm.202418064

[184] Y. Bao, M. Li, H. Jin, X. Wang, J. Zeng et al., Directional charge carrier management enabled by orderly arranged perovskite heterodomain with defined size for self-powered photodetectors. Adv. Funct. Mater. **34**(44), 2404697 (2024). 10.1002/adfm.202404697

[185] H. Zhu, H. Chen, J. Fei, Y. Deng, T. Yang et al., Solution-processed CsPbBr_3_ perovskite films* vi*a CsBr intercalated PbB_r_2 intermediate for high-performance photodetectors towards underwater wireless optical communication. Nano Energy **125**, 109513 (2024). 10.1016/j.nanoen.2024.109513

[186] J.-S. Nam, W. Jang, J. Han, B.G. Kim, J.H. Lim et al., Enhanced photodetection and air stability of lead-free tin perovskite photodiodes *via* germanium incorporation and organic cation-mediated dimensionality control. Adv. Funct. Mater. **34**(44), 2407299 (2024). 10.1002/adfm.202407299

[187] H. Li, Y. Gao, X. Hong, K. Ke, Z. Ye et al., Rational composition engineering for high-quality Pb–Sn photodetector toward sensitive near-infrared digital imaging arrays. InfoMat **7**(1), e12615 (2025). 10.1002/inf2.12615

[188] W. Gao, X. Liu, H. Jin, W. Li, X. Wang et al., Seed-crystal-assisted space-confined growth of FASnI3 quasi-single-crystal thick films and their photodetection characteristics. ACS Energy Lett. **9**(10), 5045–5055 (2024). 10.1021/acsenergylett.4c01596

[189] J. Zhou, Y. Lin, F. Wan, X. Peng, J. Sun et al., Low-noise electrically modulated dual-mode photodetectors with perovskite/organic dual absorption layers. ACS Nano **19**(48), 41298–41307 (2025). 10.1021/acsnano.5c15681 41264882

[190] Y. Hu, X. Zhang, X. Mo, J. Chu, X. Fang et al., High-purity photoactive α-phase for flexible perovskite photodetectors with modified electron transport layer. Adv. Funct. Mater. **35**(8), 2412015 (2025). 10.1002/adfm.202412015

[191] Y. Wang, B.-T. Xu, Z.-Y. Song, W.-Z. Liu, W.-S. Shen et al., Dipole regulation enables efficient near-infrared perovskite quantum dots photodetector. Adv. Funct. Mater. **36**(4), e14588 (2026). 10.1002/adfm.202514588

[192] H. Chen, Y. Wang, H. Huang, Z. Ye, B. Deng et al., Viologen phosphoric acid salt modified PEDOT: PSS for high-performance 3D perovskite photodetectors. InfoMat **7**(12), e70046 (2025). 10.1002/inf2.70046

[193] C. Bai, Z. Peng, W. Li, B. Chen, Y. Fu et al., Bidirectional-sensitive dual-narrowband self-powered single perovskite photodetector for fast computational imaging. Adv. Funct. Mater. **35**(25), 2419958 (2025). 10.1002/adfm.202419958

[194] Y. Xi, G. Li, J. Jiao, T. Ji, Y. Hao et al., 2D perovskite/organic heterojunction black-light level photodetector with record-high detectivity. Adv. Funct. Mater. **35**(26), 2418614 (2025). 10.1002/adfm.202418614

[195] E. Hong, W.D.J. Tremlett, L. Hart, B. Hu, Z. Qiao et al., Ferrocene derivatives enable ultrasensitive perovskite photodetectors with enhanced reverse bias stability. Adv. Funct. Mater. **36**(21), 2424556 (2026). 10.1002/adfm.202424556

[196] K. Ke, Y. Gao, J. Meng, Y. He, B. Deng et al., High-performance quasi-2D Sn-Pb perovskite photodetectors for high-fidelity image sensing and optical communication. Adv. Funct. Mater. **35**(27), 2424340 (2025). 10.1002/adfm.202424340

[197] H. Ren, Y. Li, Y. Chen, R. Wei, D. Kuang et al., Wearable health monitoring system based on self-powered bi-based perovskite photodetector. Nano Energy **151**, 111835 (2026). 10.1016/j.nanoen.2026.111835

[198] M.F. Al Fattah, A.A. Khan, S. Khamgaonkar, M.H. Bagheri, D. Khan et al., Photoconductivity-driven quantum efficiency gain in inorganic ruddlesden-popper layered Cs_2_PbBr_2_I_2_ perovskite photodetector for visible light detection. Adv. Funct. Mater. **36**(33), e22928 (2026). 10.1002/adfm.202522928

[199] H. Bao, Y. Sun, L. Chu, J. Liu, Z. Chen et al., Ultrasensitive Sn-Pb perovskite photodetector for monolithic near-infrared imaging. Nano Energy **149**, 111705 (2026). 10.1016/j.nanoen.2026.111705

[200] R.K. Gunasekaran, J. Nam, M.-G. Choi, W.C. Choi, S. Kim et al., Monolithic integration of redox-stable Sn-Pb halide perovskite single-crystalline films for durable near-infrared photodetection. Nano-Micro Lett. **18**(1), 141 (2026). 10.1007/s40820-025-01991-y PMC12791097 41521265

[201] R. Mastria, K.J. Riisnaes, A. Bacon, I. Leontis, H.T. Lam et al., Real time and highly sensitive sub-wavelength 2D hybrid perovskite photodetectors. Adv. Funct. Mater. **34**(36), 2401903 (2024). 10.1002/adfm.202401903

[202] X. He, S. Hao, D. Ouyang, S. Liu, N. Zhang et al., Universal vapor-phase synthesis of large-scale ultrathin perovskites with superior stability for photodetectors and image sensors. Adv. Funct. Mater. **34**(26), 2313163 (2024). 10.1002/adfm.202313163

[203] F. Huang, C. Liu, Q. Yang, Y. Chao, G. Hu et al., Lead-based perovskites significantly suppress dark currents toward high-performance multilayer SnS_2_/perovskite photodetectors. ACS Nano **19**(11), 10989–11000 (2025). 10.1021/acsnano.4c16539 40088454

[204] D. Chu, N. Liu, S. Xie, Y. Li, J. Chen et al., Stable and ultrasensitive X-ray detectors based on oriented single-crystal perovskite rods. Adv. Mater. **37**(27), 2500101 (2025). 10.1002/adma.202500101 40269573

[205] Z. Xu, H. Xi, X. Sun, H. Liu, J. Liu et al., Highly stable and sensitive (PEA)_2_PbBr4/CsPbBr_3_ single-crystal heterojunction X-ra_*y*_ detector with ultra-low detection limit. Adv. Funct. Mater. **34**(34), 2400817 (2024). 10.1002/adfm.202400817

[206] Y.-H. Huang, S.-Y. Zou, C.-Y. Sheng, Y.-C. Fang, X.-D. Wang et al., Lattice anchoring stabilizes α-FAPbI_3_ perovskite for high-performance X-ray detectors. Nano-Micro Lett. **18**(1), 14 (2025). 10.1007/s40820-025-01856-4 PMC12307842 40728710

[207] J. Zhang, W. Li, Y. Yang, Z. Tang, H. Wei et al., Integrated underwater X-ray detector arrays fabricated by perovskite single-crystal arrays. Adv. Funct. Mater. **36**(21), 2422003 (2026). 10.1002/adfm.202422003

[208] Y. Chen, Y. Li, Y. Yang, X. Yang, C. Shan et al., High-performance flexible deep ultraviolet photodetector based on crossed microwires heterojunction for real-time target trajectory tracking system. Nano Energy **138**, 110833 (2025). 10.1016/j.nanoen.2025.110833

[209] Z. He, H. Duan, J. Zeng, J. Zhou, X. Zhong et al., Perovskite retinomorphic image sensor for embodied intelligent vision. Sci. Adv. **11**(1), eads2834 (2025). 10.1126/sciadv.ads2834 PMC11698084 39752496

[210] C. Li, W. Li, W. Qu, H. Hu, J. Zong et al., Stable and lead-free perovskite hemispherical photodetector for vivid Fourier imaging. Adv. Sci. **12**(7), 2414430 (2025). 10.1002/advs.202414430 PMC11831509 39717012

[211] Z. Zhang, P. Zheng, S.-S. Yan, B.-S. Qiao, K.W. Ng et al., Ultrasensitive perovskite photodetector for filter-free color single-pixel imaging. Adv. Opt. Mater. **11**(2), 2201847 (2023). 10.1002/adom.202201847

[212] Z. Ji, Y. Liu, M. Yao, Z. Zhang, J. Zhong et al., Achieving 256 × 256-pixel color images by perovskite-based photodetectors coupled with algorithms. Adv. Funct. Mater. **31**(37), 2104320 (2021). 10.1002/adfm.202104320

[213] Y. Liu, Y. Gao, J. Zhi, R. Huang, W. Li et al., All-inorganic lead-free NiO_*x*_/Cs_3_Bi_2_Br_9_ perovskite heterojunction photodetectors for ultraviolet multispectral imaging. Nano Res. **15**(2), 1094–1101 (2022). 10.1007/s12274-021-3608-4

[214] Y. Liu, Z. Ji, G. Cen, H. Sun, H. Wang et al., Perovskite-based color camera inspired by human visual cells. Light Sci. Appl. **12**(1), 43 (2023). 10.1038/s41377-023-01072-y PMC9929324 36788229

[215] Q. Liu, Q. Wei, H. Ren, L. Zhou, Y. Zhou et al., Circular polarization-resolved ultraviolet photonic artificial synapse based on chiral perovskite. Nat. Commun. **14**(1), 7179 (2023). 10.1038/s41467-023-43034-3 PMC10630371 37935714

[216] A.J.J.M. van Breemen, R. Ollearo, S. Shanmugam, B. Peeters, L.C.J.M. Peters et al., A thin and flexible scanner for fingerprints and documents based on metal halide perovskites. Nat. Electron. **4**(11), 818–826 (2021). 10.1038/s41928-021-00662-1

[217] Y. Li, Z. Shi, L. Lei, S. Li, D. Yang et al., Ultrastable lead-free double perovskite photodetectors with imaging capability. Adv. Mater. Interfaces **6**(10), 1900188 (2019). 10.1002/admi.201900188

[218] Q. Lu, Y. Zhang, G. Yang, M. Xiong, W. Wu et al., Large-scale, uniform-patterned CsCu_2_I_3_ films for flexible solar-blind photodetectors array with ultraweak light sensing. Small **19**(27), 2300364 (2023). 10.1002/smll.202300364 36987976

[219] Z. Ji, Y. Liu, W. Li, C. Zhao, W. Mai, Reducing current fluctuation of Cs_3_Bi_2_Br_9_ perovskite photodetectors for diffuse reflection imaging with wide dynamic range. Sci. Bull. **65**(16), 1371–1379 (2020). 10.1016/j.scib.2020.04.018 36659216

[220] X. Song, C. Li, X. Zhong, R. Zhang, T. Ji et al., Gate and bias voltage-modulation photodetector based on Cs_3_Bi_2_Br_9_ for multifunctional optoelectronic logic gate. Adv. Funct. Mater. **35**(35), 2502415 (2025). 10.1002/adfm.202502415

[221] H. Sun, W. Tian, X. Wang, K. Deng, J. Xiong et al., *In situ* formed gradient bandgap-tunable perovskite for ultrahigh-speed color/spectrum-sensitive photodetectors *via* electron-donor control. Adv. Mater. **32**(14), e1908108 (2020). 10.1002/adma.201908108 32080927

[222] L. Guo, H. Sun, M. Wang, L. Min, Y. Zhou et al., Polarization and spectrum multi-dimensional sensitive perovskite photodetectors. Adv. Opt. Mater. **12**(7), 2301998 (2024). 10.1002/adom.202301998

[223] W.-H. Gao, C. Chen, Perovskites and their constructed near-infrared photodetectors. Nano Energy **128**, 109904 (2024). 10.1016/j.nanoen.2024.109904

[224] H. Park, C.B. Lee, S.-J. Lim, H. Kim, K. Kim et al., Near-infrared photodetection in tin halide perovskites *via* compositional engineering. Mater. Sci. Eng. R. Rep. **165**, 101013 (2025). 10.1016/j.mser.2025.101013

[225] Y. Li, J. Wang, Q. Yang, G. Shen, Flexible artificial optoelectronic synapse based on lead-free metal halide nanocrystals for neuromorphic computing and color recognition. Adv. Sci. **9**(22), 2202123 (2022). 10.1002/advs.202202123 PMC9353487 35661449

[226] Y. Chen, Y. Li, S. Niu, X. Yang, W. Dou et al., High temperature resistant solar-blind ultraviolet photosensor for neuromorphic computing and cryptography. Adv. Funct. Mater. **34**(24), 2315383 (2024). 10.1002/adfm.202315383

[227] Y. Hu, Q. Zhang, J. Han, X. Lian, H. Lv et al., Recent progress on stability and applications of flexible perovskite photodetectors. J. Semicond. **46**(1), 011601 (2025). 10.1088/1674-4926/24080019

[228] L. Gu, S. Poddar, Y. Lin, Z. Long, D. Zhang et al., A biomimetic eye with a hemispherical perovskite nanowire array retina. Nature **581**(7808), 278–282 (2020). 10.1038/s41586-020-2285-x 32433619

[229] J. Park, M.S. Kim, J. Kim, S. Chang, M. Lee et al., Avian eye-inspired perovskite artificial vision system for foveated and multispectral imaging. Sci. Robot. **9**(90), eadk6903 (2024). 10.1126/scirobotics.adk6903 38809996

[230] Y. Zhou, Z. Sun, Y. Ding, Z. Yuan, X. Qiu et al., An ultrawide field-of-view pinhole compound eye using hemispherical nanowire array for robot vision. Sci. Robot. **9**(90), eadi8666 (2024). 10.1126/scirobotics.adi8666 38748782

[231] Y. Yang, W. Ran, Y. Li, Y. Chen, D. Chen et al., Multi-dimensional visual information processing under complex light environments using time-evolved polarization-sensitive synaptic electronics. Nat. Commun. **16**(1), 5665 (2025). 10.1038/s41467-025-61361-5 PMC12216041 40593904

[232] H. Kim, R.M. Kim, S.D. Namgung, N.H. Cho, J.B. Son et al., Ultrasensitive near-infrared circularly polarized light detection using 3D perovskite embedded with chiral plasmonic nanoparticles. Adv. Sci. **9**(5), 2104598 (2022). 10.1002/advs.202104598 PMC8844506 34978155

[233] J. Ren, B. Wan, Q. Cheng, R. Geng, X. Zhao et al., Progress on perovskite photodetectors for optical communications. Nanoscale **18**(4), 1693–1739 (2026). 10.1039/d5nr03957f 41358450

[234] H.-Y. Hou, S. Tian, H.-R. Ge, J.-D. Chen, Y.-Q. Li et al., Recent progress of polarization-sensitive perovskite photodetectors. Adv. Funct. Mater. **32**(48), 2209324 (2022). 10.1002/adfm.202209324

[235] L. Bian, F. Cao, H. Zhao, F. Xiang, H. Sun et al., Self-powered perovskite/Si bipolar response photodetector for visible and near-infrared dual-band imaging and secure optical communication. Laser Photonics Rev. **19**(2), 2401331 (2025). 10.1002/lpor.202401331

[236] J. Ma, F. Zhang, Y. Xing, H. Jiang, Y. Tian et al., Self-powered UV dual-band photodetector based on Cs_3_BiCl_6_/GaN heterojunction for logical operation and encrypted photo-communication. Adv. Sci. **12**(28), 2503498 (2025). 10.1002/advs.202503498 PMC12302572 40349180

[237] Z. Lv, H. Gao, Y. Hu, Y. Fan, G. Pan et al., Ultraviolet–visible-near-infrared broadband photodetector enabled by Cs_2_AgBiBr 6: Sn/conjugated polymer heterojunction. ACS Appl. Mater. Interfaces **16**(38), 51055–51064 (2024). 10.1021/acsami.4c09281 39276066

[238] W. Hu, R. Yan, Y. Ma, R. Cong, X. Ning et al., Vertical pyro-phototronic effect and lateral photothermoelectric effect in perovskite single crystals-based photodetector for narrowband and broadband dual-modal optical communications. Laser Photonics Rev. **19**(15), 2401990 (2025). 10.1002/lpor.202401990

[239] Y. Pan, X. Wang, D. Zhang, Z. Wei, Y. Xu et al., Visible elimination, ultraviolet and near-infrared dual-band photodetector based on single-crystal perovskite heterojunctions toward secure optical communication. ACS Photonics **11**(3), 1252–1263 (2024). 10.1021/acsphotonics.3c01768

[240] C.H. Kang, I. Dursun, G. Liu, L. Sinatra, X. Sun et al., High-speed colour-converting photodetector with all-inorganic CsPbBr_3_ perovskite nanocrystals for ultraviolet light communication. Light. Sci. Appl. **8**, 94 (2019). 10.1038/s41377-019-0204-4 PMC6804731 31645937

[241] R. Zhang, N. Hu, H. Zhou, K. Zou, X. Su et al., Turbulence-resilient pilot-assisted self-coherent free-space optical communications using automatic optoelectronic mixing of many modes. Nat. Photon. **15**(10), 743–750 (2021). 10.1038/s41566-021-00877-w

[242] J. Zhang, J. Zhang, H. Wei, Perovskite photodetectors on skin: current advances and commercialization challenges. Sci. China Mater. **68**(12), 4364–4370 (2025). 10.1007/s40843-025-3570-4

[243] Y. Zheng, Y. Wang, Z. Li, Z. Yuan, S. Guo et al., MXene quantum dots/perovskite heterostructure enabling highly specific ultraviolet detection for skin prevention. Matter **6**(2), 506–520 (2023). 10.1016/j.matt.2022.11.020

[244] Y. Zhou, X. Qiu, Z. Wan, Z. Long, S. Poddar et al., Halide-exchanged perovskite photodetectors for wearable visible-blind ultraviolet monitoring. Nano Energy **100**, 107516 (2022). 10.1016/j.nanoen.2022.107516

[245] Y. Tang, P. Jin, Y. Wang, D. Li, Y. Chen et al., Enabling low-drift flexible perovskite photodetectors by electrical modulation for wearable health monitoring and weak light imaging. Nat. Commun. **14**(1), 4961 (2023). 10.1038/s41467-023-40711-1 PMC10432415 37587158

[246] G. Hu, L. Zhang, X. Xu, Z. Kang, H. Chen et al., Highly sensitive perovskite nanowire photodetectors enabled by surface state engineering for non-contact heart rate monitoring. Small **21**(38), e03711 (2025). 10.1002/smll.202503711 40755035

[247] M. Zayat, P. Garcia-Parejo, D. Levy, Preventing UV-light damage of light sensitive materials using a highly protective UV-absorbing coating. Chem. Soc. Rev. **36**(8), 1270–1281 (2007). 10.1039/b608888k 17619687

[248] J. Kim, G.A. Salvatore, H. Araki, A.M. Chiarelli, Z. Xie et al., Battery-free, stretchable optoelectronic systems for wireless optical characterization of the skin. Sci. Adv. **2**(8), e1600418 (2016). 10.1126/sciadv.1600418 PMC4972468 27493994

[249] R. Liu, F. Li, F. Zeng, R. Zhao, R. Zheng, Halide perovskite X-ray detectors: fundamentals, progress, and outlook. Appl. Phys. Rev. **11**(2), 021327 (2024). 10.1063/5.0198695

[250] X. Ou, F. Gao, Blossoms in perovskite planar X-ray detectors. Nat. Commun. **15**(1), 5754 (2024). 10.1038/s41467-024-50179-2 PMC11233596 38982081

[251] X. Wang, W. Wei, S. Zhang, Z. Li, J. Wang et al., Self-powered ferroelectric bi-based perovskite X-ray detector for radiation monitoring and intelligent imaging recognition. Laser Photonics Rev. **20**(2), e01217 (2026). 10.1002/lpor.202501217

[252] H. Wu, X. Chen, Z. Song, A. Zhang, X. Du et al., Mechanochemical synthesis of high-entropy perovskite toward highly sensitive and stable X-ray flat-panel detectors. Adv. Mater. **35**(29), e2301406 (2023). 10.1002/adma.202301406 37022336

[253] Y. Liu, C. Gao, D. Li, X. Zhang, J. Zhu et al., Dynamic X-ray imaging with screen-printed perovskite CMOS array. Nat. Commun. **15**(1), 1588 (2024). 10.1038/s41467-024-45871-2 PMC10881555 38383659

[254] J. Zhao, L. Zhao, Y. Deng, X. Xiao, Z. Ni et al., Perovskite-filled membranes for flexible and large-area direct-conversion X-ray detector arrays. Nat. Photonics **14**(10), 612–617 (2020). 10.1038/s41566-020-0678-x

[255] P. Tan, T. Liu, Y. Yang, Y. Chen, Y. Guan et al., Flexible soft X-ray image sensors based on metal halide perovskites with high quantum efficiency. Adv. Mater. **36**(48), 2470386 (2024). 10.1002/adma.202470386 39363637

[256] D. Zhao, Y.-W. Choi, T. Yokota, T. Someya, N.-G. Park, Halide perovskite radiation detectors: conventional imaging applications and new opportunities. Adv. Funct. Mater. **36**(6), e13676 (2026). 10.1002/adfm.202513676

[257] H. Sun, C. Li, L. Li, X. Zhang, J. Luo et al., The development of customized perovskite photodetectors. Nat. Electron. **8**(12), 1170–1181 (2025). 10.1038/s41928-025-01517-9

[258] M. Krishnaiah, S. Kim, A. Kumar, D. Mishra, S.G. Seo et al., Physically detachable and operationally stable Cs_2_SnI_6_ photodetector arrays integrated with µ-LEDs for broadband flexible optical systems. Adv. Mater. **34**(17), 2109673 (2022). 10.1002/adma.202109673 35246891

[259] X. Li, Y. Mai, C. Lan, F. Yang, P. Zhang et al., Machine learning-assisted design of high-performance perovskite photodetectors: a review. Adv. Compos. Hybrid Mater. **8**(1), 27 (2024). 10.1007/s42114-024-01113-z

[260] Q.-Q. Chu, Z. Sun, D. Wang, B. Cheng, H. Wang et al., Encapsulation: the path to commercialization of stable perovskite solar cells. Matter **6**(11), 3838–3863 (2023). 10.1016/j.matt.2023.08.016

[261] V. Pecunia, T.D. Anthopoulos, A. Armin, B. Bouthinon, M. Caironi et al., Guidelines for accurate evaluation of photodetectors based on emerging semiconductor technologies. Nat. Photonics **19**(11), 1178–1188 (2025). 10.1038/s41566-025-01759-1

[262] Y. Li, X. He, S.-C. Chen, N. Zhao, Intelligent photodetectors: postmanufacturing tunability toward enhanced performance and advanced functions. Chem. Rev. **125**(15), 6977–7022 (2025). 10.1021/acs.chemrev.4c00763 PMC12355702 40686380

